# Supplementary material for: The genome of the crustacean Parhyale hawaiensis, a model for animal development, regeneration, immunity and lignocellulose digestion
Source: eLife. 2016 Nov 16;5:e20062. doi: 10.7554/eLife.20062 (PMC5111886; doi:10.7554/eLife.20062)
Supplement: Source code 1. — Includes bioinformatic processsing of raw read data, k-mer analysis, contig assembly, scaffolding and CEGMA cased representation analyis. DOI: http://dx.doi.org/10.7554/eLife.20062.046 [file elife-20062-code1.htm]

Notebook


In [2]:

```
import gzip, os, sys
from IPython.display import HTML
import pandas as pd
import seaborn as sns
import matplotlib.pyplot as plt
import scipy.stats as ss
import scipy as sp
from collections import defaultdict

#import custom functions for displaying tables, bash commands
sys.path.append(os.path.abspath("/home/damian/"))
from dk_ipython import *
%matplotlib inline
HTML(addToggle())
```

Out[2]:

The raw code for this IPython notebook is by default hidden for easier reading.
To toggle on/off the raw code, click here.

# Parhyale hawaiensis genome assembly

1. Raw data processing

   - split/rename raw files
   - Trimmomatic on SGL
   - NextClip on MPL
2. K-mer analysis

   - K-mer spectra on Short-SGL, Long-SGL MPLs
   - K-mer content intersection of libraries
3. Contig assembly

   - Abyss setup on AWS
   - Assembly Length stats
   - Assembly k-mer coverage stats
   - MPL fragment size estimation
   - Contig coverage
4. Scaffolding

   - SSPACE scaffolding
   - Scaffolding stats
5. CEGMA

   - CEGMA of transcriptome
   - CEGMA of genome

In [7]:

```
prefix = '/home/share/projects/phaw_genome/draft_2.3/'
```

### Raw data processing

#### Shotgun libraries

8 shotgun libraries (SGL) were made from a single male animal (sample 4). The 500bp insert libraries (short-sgl) are actually 646bp inserts. The 800bp (long-sgl) are actually 1100bp inserts.

A total of 10 files for P. Hawaiensis genome was downloaded from Anastasios's dropbox account:

```
Sample_4_AP_500bp_Lane1_1
Sample_4_AP_500bp_Lane1_2
Sample_4_AP_500bp_Lanes2-3_1
Sample_4_AP_500bp_Lanes2-3_2 
Sample_4_AP_800bp_Lane1_1
Sample_4_AP_800bp_Lane1_2
Sample_4_AP_500bp_Lanes4-5_1
Sample_4_AP_500bp_Lanes4-5_2 
Sample_4_AP_800bp_Lane2-3_1 
Sample_4_AP_800bp_Lane2-3_2
```

`Sample_4_AP_500bp_Lanes2-3, Sample_4_AP_500bp_Lanes4-5, Sample_4_AP_800bp_Lane2-3` consist of sequences from two lanes.

Illumina headers are in the format:

```
MachineID : Run ID : Flow cell ID : Lane ID : Tile ID : X Location : Y Location Pair

Ex. HWI-ST651:295:HAE2KADXX:1:1101:1157:2016
```

`Sample_4_AP_500bp_Lanes2-3` contains two lanes:

```
@HWI-ST651:295:HAE2KADXX:1:
@HWI-ST651:295:HAE2KADXX:2:
```

#### Mate-pair libraries

From Anastasios' e-mail:

```
Sample_4_AP_6kb_Lanes1-2_1 (reads from 5-7kb MPL lanes 1 and 2 from one end)
Sample_4_AP_6kb_Lanes1-2_2 (reads from 5-7kb MPL lanes 1 and 2 from other end)
Sample_4_AP_6kb_Lanes3-4_1 (reads from 5-7kb MPL lanes 3 and 4 from one end)
Sample_4_AP_6kb_Lanes3-4_2 (reads from 5-7kb MPL lanes 3 and 4 from other end)
Sample_4_AP_8kb_Lanes1-2_1 (reads from 7-9kb MPL lanes 1 and 2 from one end)
Sample_4_AP_8kb_Lanes1-2_2 (reads from 7-9kb MPL lanes 1 and 2 from other end)
Sample_4_AP_8kb_Lanes3-4_1 (reads from 7-9kb MPL lanes 3 and 4 from one end)
Sample_4_AP_8kb_Lanes3-4_2 (reads from 7-9kb MPL lanes 3 and 4 from other end)
Sample_4_AP_9kb_Lanes1-2_1 (reads from 9-12kb MPL lanes 1 and 2 from one end)
Sample_4_AP_9kb_Lanes1-2_2 (reads from 9-12kb MPL lanes 1 and 2 from other end)
Sample_4_AP_9kb_Lanes3-4_1 (reads from 9-12kb MPL lanes 3 and 4 from one end)
Sample_4_AP_9kb_Lanes3-4_2 (reads from 9-12kb MPL lanes 3 and 4 from other end)
Sample_4_AP_20kb_Lanes1-2_1 (reads from ?kb MPL lanes 1 and 2 from one end)
Sample_4_AP_20kb_Lanes1-2_2 (reads from ?kb MPL lanes 1 and 2 from other end)
Sample_4_AP_20kb_Lanes3-4_1 (reads from ?kb MPL lanes 3 and 4 from one end)
Sample_4_AP_20kb_Lanes3-4_2 (reads from ?kb MPL lanes 3 and 4 from other end)
```

A total of 16 mate pair libraries for download:

```
Sample_4_AP_20kb_Lanes1-2_1.fastq.gz
Sample_4_AP_20kb_Lanes1-2_2.fastq.gz
Sample_4_AP_20kb_Lanes3-4_1.fastq.gz
Sample_4_AP_20kb_Lanes3-4_2.fastq.gz
Sample_4_AP_6kb_Lanes1-2_1.fastq.gz
Sample_4_AP_6kb_Lanes1-2_2.fastq.gz
Sample_4_AP_6kb_Lanes3-4_1.fastq.gz
Sample_4_AP_6kb_Lanes3-4_2.fastq.gz
Sample_4_AP_8kb_Lanes1-2_1.fastq.gz
Sample_4_AP_8kb_Lanes1-2_2.fastq.gz
Sample_4_AP_8kb_Lanes3-4_1.fastq.gz
Sample_4_AP_8kb_Lanes3-4_2.fastq.gz
Sample_4_AP_9kb_Lanes1-2_1.fastq.gz
Sample_4_AP_9kb_Lanes1-2_2.fastq.gz
Sample_4_AP_9kb_Lanes3-4_1.fastq.gz
Sample_4_AP_9kb_Lanes3-4_2.fastq.gz
```

Samples came from a single male animal (sample 4).

All MPL samples contain two libraries.

Files containing multiple lanes are splitted into individual files by lane. This was done by first finding the two library header names and then distributing the fastq entries according to the header names.

#### Rename libraries

The files were renamed in the format of:

In [127]:

```
renameCmds = '''ln -s raw/pilot/Sample_4_AP_500bp_Lane1_1.fastq.gz AP_phaw_S4_shortsgl_01_A.fastq.gz
ln -s raw/pilot/Sample_4_AP_500bp_Lane1_2.fastq.gz AP_phaw_S4_shortsgl_01_B.fastq.gz
ln -s raw/pilot/Sample_4_AP_500bp_Lanes2-3_1_A.fastq.gz AP_phaw_S4_shortsgl_02_A.fastq.gz
ln -s raw/pilot/Sample_4_AP_500bp_Lanes2-3_1_B.fastq.gz AP_phaw_S4_shortsgl_03_A.fastq.gz
ln -s raw/pilot/Sample_4_AP_500bp_Lanes2-3_2_A.fastq.gz AP_phaw_S4_shortsgl_02_B.fastq.gz
ln -s raw/pilot/Sample_4_AP_500bp_Lanes2-3_2_B.fastq.gz AP_phaw_S4_shortsgl_03_B.fastq.gz
ln -s raw/pilot/Sample_4_AP_500bp_Lanes4-5_1_A.fastq.gz AP_phaw_S4_shortsgl_04_A.fastq.gz
ln -s raw/pilot/Sample_4_AP_500bp_Lanes4-5_1_B.fastq.gz AP_phaw_S4_shortsgl_05_A.fastq.gz
ln -s raw/pilot/Sample_4_AP_500bp_Lanes4-5_2_A.fastq.gz AP_phaw_S4_shortsgl_04_B.fastq.gz
ln -s raw/pilot/Sample_4_AP_500bp_Lanes4-5_2_B.fastq.gz AP_phaw_S4_shortsgl_05_B.fastq.gz
ln -s raw/Sample_4_AP_500bp_Lanes6-7_1_Asplit.fastq.gz AP_phaw_S4_shortsgl_06_A.fastq.gz
ln -s raw/Sample_4_AP_500bp_Lanes6-7_2_Asplit.fastq.gz AP_phaw_S4_shortsgl_06_B.fastq.gz
ln -s raw/Sample_4_AP_500bp_Lanes6-7_1_Bsplit.fastq.gz AP_phaw_S4_shortsgl_07_A.fastq.gz
ln -s raw/Sample_4_AP_500bp_Lanes6-7_2_Bsplit.fastq.gz AP_phaw_S4_shortsgl_07_B.fastq.gz
ln -s raw/Sample_4_AP_500bp_Lanes8-9_1_Asplit.fastq.gz AP_phaw_S4_shortsgl_08_A.fastq.gz
ln -s raw/Sample_4_AP_500bp_Lanes8-9_2_Asplit.fastq.gz AP_phaw_S4_shortsgl_08_B.fastq.gz
ln -s raw/Sample_4_AP_500bp_Lanes8-9_1_Bsplit.fastq.gz AP_phaw_S4_shortsgl_09_A.fastq.gz
ln -s raw/Sample_4_AP_500bp_Lanes8-9_2_Bsplit.fastq.gz AP_phaw_S4_shortsgl_09_B.fastq.gz
ln -s raw/pilot/Sample_4_AP_800bp_Lane1_1.fastq.gz AP_phaw_S4_longsgl_01_A.fastq.gz
ln -s raw/pilot/Sample_4_AP_800bp_Lane1_2.fastq.gz AP_phaw_S4_longsgl_01_B.fastq.gz
ln -s raw/pilot/Sample_4_AP_800bp_Lanes2-3_1_A.fastq.gz AP_phaw_S4_longsgl_02_A.fastq.gz
ln -s raw/pilot/Sample_4_AP_800bp_Lanes2-3_1_B.fastq.gz AP_phaw_S4_longsgl_03_A.fastq.gz
ln -s raw/pilot/Sample_4_AP_800bp_Lanes2-3_2_A.fastq.gz AP_phaw_S4_longsgl_02_B.fastq.gz
ln -s raw/pilot/Sample_4_AP_800bp_Lanes2-3_2_B.fastq.gz AP_phaw_S4_longsgl_03_B.fastq.gz
ln -s raw/Sample_4_AP_20kb_Lanes1-2_1_Asplit.fastq.gz AP_phaw_S4_20kbmpl_01_A.fastq.gz
ln -s raw/Sample_4_AP_20kb_Lanes1-2_1_Bsplit.fastq.gz AP_phaw_S4_20kbmpl_02_A.fastq.gz
ln -s raw/Sample_4_AP_20kb_Lanes1-2_2_Asplit.fastq.gz AP_phaw_S4_20kbmpl_01_B.fastq.gz
ln -s raw/Sample_4_AP_20kb_Lanes1-2_2_Bsplit.fastq.gz AP_phaw_S4_20kbmpl_02_B.fastq.gz
ln -s raw/Sample_4_AP_20kb_Lanes3-4_1_Asplit.fastq.gz AP_phaw_S4_20kbmpl_03_A.fastq.gz
ln -s raw/Sample_4_AP_20kb_Lanes3-4_1_Bsplit.fastq.gz AP_phaw_S4_20kbmpl_04_A.fastq.gz
ln -s raw/Sample_4_AP_20kb_Lanes3-4_2_Asplit.fastq.gz AP_phaw_S4_20kbmpl_03_B.fastq.gz
ln -s raw/Sample_4_AP_20kb_Lanes3-4_2_Bsplit.fastq.gz AP_phaw_S4_20kbmpl_04_B.fastq.gz
ln -s raw/Sample_4_AP_6kb_Lanes1-2_1_Asplit.fastq.gz AP_phaw_S4_6kbmpl_01_A.fastq.gz
ln -s raw/Sample_4_AP_6kb_Lanes1-2_1_Bsplit.fastq.gz AP_phaw_S4_6kbmpl_02_A.fastq.gz
ln -s raw/Sample_4_AP_6kb_Lanes1-2_2_Asplit.fastq.gz AP_phaw_S4_6kbmpl_01_B.fastq.gz
ln -s raw/Sample_4_AP_6kb_Lanes1-2_2_Bsplit.fastq.gz AP_phaw_S4_6kbmpl_02_B.fastq.gz
ln -s raw/Sample_4_AP_6kb_Lanes3-4_1_Asplit.fastq.gz AP_phaw_S4_6kbmpl_03_A.fastq.gz
ln -s raw/Sample_4_AP_6kb_Lanes3-4_1_Bsplit.fastq.gz AP_phaw_S4_6kbmpl_04_A.fastq.gz
ln -s raw/Sample_4_AP_6kb_Lanes3-4_2_Asplit.fastq.gz AP_phaw_S4_6kbmpl_03_B.fastq.gz
ln -s raw/Sample_4_AP_6kb_Lanes3-4_2_Bsplit.fastq.gz AP_phaw_S4_6kbmpl_04_B.fastq.gz
ln -s raw/Sample_4_AP_8kb_Lanes1-2_1_Asplit.fastq.gz AP_phaw_S4_8kbmpl_01_A.fastq.gz
ln -s raw/Sample_4_AP_8kb_Lanes1-2_1_Bsplit.fastq.gz AP_phaw_S4_8kbmpl_02_A.fastq.gz
ln -s raw/Sample_4_AP_8kb_Lanes1-2_2_Asplit.fastq.gz AP_phaw_S4_8kbmpl_01_B.fastq.gz
ln -s raw/Sample_4_AP_8kb_Lanes1-2_2_Bsplit.fastq.gz AP_phaw_S4_8kbmpl_02_B.fastq.gz
ln -s raw/Sample_4_AP_8kb_Lanes3-4_1_Asplit.fastq.gz AP_phaw_S4_8kbmpl_03_A.fastq.gz
ln -s raw/Sample_4_AP_8kb_Lanes3-4_1_Bsplit.fastq.gz AP_phaw_S4_8kbmpl_04_A.fastq.gz
ln -s raw/Sample_4_AP_8kb_Lanes3-4_2_Asplit.fastq.gz AP_phaw_S4_8kbmpl_03_B.fastq.gz
ln -s raw/Sample_4_AP_8kb_Lanes3-4_2_Bsplit.fastq.gz AP_phaw_S4_8kbmpl_04_B.fastq.gz
ln -s raw/Sample_4_AP_9kb_Lanes1-2_1_Asplit.fastq.gz AP_phaw_S4_9kbmpl_01_A.fastq.gz
ln -s raw/Sample_4_AP_9kb_Lanes1-2_1_Bsplit.fastq.gz AP_phaw_S4_9kbmpl_02_A.fastq.gz
ln -s raw/Sample_4_AP_9kb_Lanes1-2_2_Asplit.fastq.gz AP_phaw_S4_9kbmpl_01_B.fastq.gz
ln -s raw/Sample_4_AP_9kb_Lanes1-2_2_Bsplit.fastq.gz AP_phaw_S4_9kbmpl_02_B.fastq.gz
ln -s raw/Sample_4_AP_9kb_Lanes3-4_1_Asplit.fastq.gz AP_phaw_S4_9kbmpl_03_A.fastq.gz
ln -s raw/Sample_4_AP_9kb_Lanes3-4_1_Bsplit.fastq.gz AP_phaw_S4_9kbmpl_04_A.fastq.gz
ln -s raw/Sample_4_AP_9kb_Lanes3-4_2_Asplit.fastq.gz AP_phaw_S4_9kbmpl_03_B.fastq.gz
ln -s raw/Sample_4_AP_9kb_Lanes3-4_2_Bsplit.fastq.gz AP_phaw_S4_9kbmpl_04_B.fastq.gz'''

HTML(textarea('Commands for renamed symbolic links',renameCmds))
```

Out[127]:

**Commands for renamed symbolic links**:  

ln -s raw/pilot/Sample\_4\_AP\_500bp\_Lane1\_1.fastq.gz AP\_phaw\_S4\_shortsgl\_01\_A.fastq.gz
ln -s raw/pilot/Sample\_4\_AP\_500bp\_Lane1\_2.fastq.gz AP\_phaw\_S4\_shortsgl\_01\_B.fastq.gz
ln -s raw/pilot/Sample\_4\_AP\_500bp\_Lanes2-3\_1\_A.fastq.gz AP\_phaw\_S4\_shortsgl\_02\_A.fastq.gz
ln -s raw/pilot/Sample\_4\_AP\_500bp\_Lanes2-3\_1\_B.fastq.gz AP\_phaw\_S4\_shortsgl\_03\_A.fastq.gz
ln -s raw/pilot/Sample\_4\_AP\_500bp\_Lanes2-3\_2\_A.fastq.gz AP\_phaw\_S4\_shortsgl\_02\_B.fastq.gz
ln -s raw/pilot/Sample\_4\_AP\_500bp\_Lanes2-3\_2\_B.fastq.gz AP\_phaw\_S4\_shortsgl\_03\_B.fastq.gz
ln -s raw/pilot/Sample\_4\_AP\_500bp\_Lanes4-5\_1\_A.fastq.gz AP\_phaw\_S4\_shortsgl\_04\_A.fastq.gz
ln -s raw/pilot/Sample\_4\_AP\_500bp\_Lanes4-5\_1\_B.fastq.gz AP\_phaw\_S4\_shortsgl\_05\_A.fastq.gz
ln -s raw/pilot/Sample\_4\_AP\_500bp\_Lanes4-5\_2\_A.fastq.gz AP\_phaw\_S4\_shortsgl\_04\_B.fastq.gz
ln -s raw/pilot/Sample\_4\_AP\_500bp\_Lanes4-5\_2\_B.fastq.gz AP\_phaw\_S4\_shortsgl\_05\_B.fastq.gz
ln -s raw/Sample\_4\_AP\_500bp\_Lanes6-7\_1\_Asplit.fastq.gz AP\_phaw\_S4\_shortsgl\_06\_A.fastq.gz
ln -s raw/Sample\_4\_AP\_500bp\_Lanes6-7\_2\_Asplit.fastq.gz AP\_phaw\_S4\_shortsgl\_06\_B.fastq.gz
ln -s raw/Sample\_4\_AP\_500bp\_Lanes6-7\_1\_Bsplit.fastq.gz AP\_phaw\_S4\_shortsgl\_07\_A.fastq.gz
ln -s raw/Sample\_4\_AP\_500bp\_Lanes6-7\_2\_Bsplit.fastq.gz AP\_phaw\_S4\_shortsgl\_07\_B.fastq.gz
ln -s raw/Sample\_4\_AP\_500bp\_Lanes8-9\_1\_Asplit.fastq.gz AP\_phaw\_S4\_shortsgl\_08\_A.fastq.gz
ln -s raw/Sample\_4\_AP\_500bp\_Lanes8-9\_2\_Asplit.fastq.gz AP\_phaw\_S4\_shortsgl\_08\_B.fastq.gz
ln -s raw/Sample\_4\_AP\_500bp\_Lanes8-9\_1\_Bsplit.fastq.gz AP\_phaw\_S4\_shortsgl\_09\_A.fastq.gz
ln -s raw/Sample\_4\_AP\_500bp\_Lanes8-9\_2\_Bsplit.fastq.gz AP\_phaw\_S4\_shortsgl\_09\_B.fastq.gz
ln -s raw/pilot/Sample\_4\_AP\_800bp\_Lane1\_1.fastq.gz AP\_phaw\_S4\_longsgl\_01\_A.fastq.gz
ln -s raw/pilot/Sample\_4\_AP\_800bp\_Lane1\_2.fastq.gz AP\_phaw\_S4\_longsgl\_01\_B.fastq.gz
ln -s raw/pilot/Sample\_4\_AP\_800bp\_Lanes2-3\_1\_A.fastq.gz AP\_phaw\_S4\_longsgl\_02\_A.fastq.gz
ln -s raw/pilot/Sample\_4\_AP\_800bp\_Lanes2-3\_1\_B.fastq.gz AP\_phaw\_S4\_longsgl\_03\_A.fastq.gz
ln -s raw/pilot/Sample\_4\_AP\_800bp\_Lanes2-3\_2\_A.fastq.gz AP\_phaw\_S4\_longsgl\_02\_B.fastq.gz
ln -s raw/pilot/Sample\_4\_AP\_800bp\_Lanes2-3\_2\_B.fastq.gz AP\_phaw\_S4\_longsgl\_03\_B.fastq.gz
ln -s raw/Sample\_4\_AP\_20kb\_Lanes1-2\_1\_Asplit.fastq.gz AP\_phaw\_S4\_20kbmpl\_01\_A.fastq.gz
ln -s raw/Sample\_4\_AP\_20kb\_Lanes1-2\_1\_Bsplit.fastq.gz AP\_phaw\_S4\_20kbmpl\_02\_A.fastq.gz
ln -s raw/Sample\_4\_AP\_20kb\_Lanes1-2\_2\_Asplit.fastq.gz AP\_phaw\_S4\_20kbmpl\_01\_B.fastq.gz
ln -s raw/Sample\_4\_AP\_20kb\_Lanes1-2\_2\_Bsplit.fastq.gz AP\_phaw\_S4\_20kbmpl\_02\_B.fastq.gz
ln -s raw/Sample\_4\_AP\_20kb\_Lanes3-4\_1\_Asplit.fastq.gz AP\_phaw\_S4\_20kbmpl\_03\_A.fastq.gz
ln -s raw/Sample\_4\_AP\_20kb\_Lanes3-4\_1\_Bsplit.fastq.gz AP\_phaw\_S4\_20kbmpl\_04\_A.fastq.gz
ln -s raw/Sample\_4\_AP\_20kb\_Lanes3-4\_2\_Asplit.fastq.gz AP\_phaw\_S4\_20kbmpl\_03\_B.fastq.gz
ln -s raw/Sample\_4\_AP\_20kb\_Lanes3-4\_2\_Bsplit.fastq.gz AP\_phaw\_S4\_20kbmpl\_04\_B.fastq.gz
ln -s raw/Sample\_4\_AP\_6kb\_Lanes1-2\_1\_Asplit.fastq.gz AP\_phaw\_S4\_6kbmpl\_01\_A.fastq.gz
ln -s raw/Sample\_4\_AP\_6kb\_Lanes1-2\_1\_Bsplit.fastq.gz AP\_phaw\_S4\_6kbmpl\_02\_A.fastq.gz
ln -s raw/Sample\_4\_AP\_6kb\_Lanes1-2\_2\_Asplit.fastq.gz AP\_phaw\_S4\_6kbmpl\_01\_B.fastq.gz
ln -s raw/Sample\_4\_AP\_6kb\_Lanes1-2\_2\_Bsplit.fastq.gz AP\_phaw\_S4\_6kbmpl\_02\_B.fastq.gz
ln -s raw/Sample\_4\_AP\_6kb\_Lanes3-4\_1\_Asplit.fastq.gz AP\_phaw\_S4\_6kbmpl\_03\_A.fastq.gz
ln -s raw/Sample\_4\_AP\_6kb\_Lanes3-4\_1\_Bsplit.fastq.gz AP\_phaw\_S4\_6kbmpl\_04\_A.fastq.gz
ln -s raw/Sample\_4\_AP\_6kb\_Lanes3-4\_2\_Asplit.fastq.gz AP\_phaw\_S4\_6kbmpl\_03\_B.fastq.gz
ln -s raw/Sample\_4\_AP\_6kb\_Lanes3-4\_2\_Bsplit.fastq.gz AP\_phaw\_S4\_6kbmpl\_04\_B.fastq.gz
ln -s raw/Sample\_4\_AP\_8kb\_Lanes1-2\_1\_Asplit.fastq.gz AP\_phaw\_S4\_8kbmpl\_01\_A.fastq.gz
ln -s raw/Sample\_4\_AP\_8kb\_Lanes1-2\_1\_Bsplit.fastq.gz AP\_phaw\_S4\_8kbmpl\_02\_A.fastq.gz
ln -s raw/Sample\_4\_AP\_8kb\_Lanes1-2\_2\_Asplit.fastq.gz AP\_phaw\_S4\_8kbmpl\_01\_B.fastq.gz
ln -s raw/Sample\_4\_AP\_8kb\_Lanes1-2\_2\_Bsplit.fastq.gz AP\_phaw\_S4\_8kbmpl\_02\_B.fastq.gz
ln -s raw/Sample\_4\_AP\_8kb\_Lanes3-4\_1\_Asplit.fastq.gz AP\_phaw\_S4\_8kbmpl\_03\_A.fastq.gz
ln -s raw/Sample\_4\_AP\_8kb\_Lanes3-4\_1\_Bsplit.fastq.gz AP\_phaw\_S4\_8kbmpl\_04\_A.fastq.gz
ln -s raw/Sample\_4\_AP\_8kb\_Lanes3-4\_2\_Asplit.fastq.gz AP\_phaw\_S4\_8kbmpl\_03\_B.fastq.gz
ln -s raw/Sample\_4\_AP\_8kb\_Lanes3-4\_2\_Bsplit.fastq.gz AP\_phaw\_S4\_8kbmpl\_04\_B.fastq.gz
ln -s raw/Sample\_4\_AP\_9kb\_Lanes1-2\_1\_Asplit.fastq.gz AP\_phaw\_S4\_9kbmpl\_01\_A.fastq.gz
ln -s raw/Sample\_4\_AP\_9kb\_Lanes1-2\_1\_Bsplit.fastq.gz AP\_phaw\_S4\_9kbmpl\_02\_A.fastq.gz
ln -s raw/Sample\_4\_AP\_9kb\_Lanes1-2\_2\_Asplit.fastq.gz AP\_phaw\_S4\_9kbmpl\_01\_B.fastq.gz
ln -s raw/Sample\_4\_AP\_9kb\_Lanes1-2\_2\_Bsplit.fastq.gz AP\_phaw\_S4\_9kbmpl\_02\_B.fastq.gz
ln -s raw/Sample\_4\_AP\_9kb\_Lanes3-4\_1\_Asplit.fastq.gz AP\_phaw\_S4\_9kbmpl\_03\_A.fastq.gz
ln -s raw/Sample\_4\_AP\_9kb\_Lanes3-4\_1\_Bsplit.fastq.gz AP\_phaw\_S4\_9kbmpl\_04\_A.fastq.gz
ln -s raw/Sample\_4\_AP\_9kb\_Lanes3-4\_2\_Asplit.fastq.gz AP\_phaw\_S4\_9kbmpl\_03\_B.fastq.gz
ln -s raw/Sample\_4\_AP\_9kb\_Lanes3-4\_2\_Bsplit.fastq.gz AP\_phaw\_S4\_9kbmpl\_04\_B.fastq.gz

In [38]:

```
print 'Table for old and new files'
nameTable = ListTable()
nameTable.append(['Old Name','New Name'])
for line in renameCmds.split('\n'):
    nameTable.append([line.strip().split()[2].split('/')[-1], line.strip().split()[-1]])
nameTable
```

```
Table for old and new files
```

Out[38]:

|  |  |
| --- | --- |
| Old Name | New Name |
| Sample\_4\_AP\_500bp\_Lane1\_1.fastq.gz | AP\_phaw\_S4\_shortsgl\_01\_A.fastq.gz |
| Sample\_4\_AP\_500bp\_Lane1\_2.fastq.gz | AP\_phaw\_S4\_shortsgl\_01\_B.fastq.gz |
| Sample\_4\_AP\_500bp\_Lanes2-3\_1\_A.fastq.gz | AP\_phaw\_S4\_shortsgl\_02\_A.fastq.gz |
| Sample\_4\_AP\_500bp\_Lanes2-3\_1\_B.fastq.gz | AP\_phaw\_S4\_shortsgl\_03\_A.fastq.gz |
| Sample\_4\_AP\_500bp\_Lanes2-3\_2\_A.fastq.gz | AP\_phaw\_S4\_shortsgl\_02\_B.fastq.gz |
| Sample\_4\_AP\_500bp\_Lanes2-3\_2\_B.fastq.gz | AP\_phaw\_S4\_shortsgl\_03\_B.fastq.gz |
| Sample\_4\_AP\_800bp\_Lane1\_1.fastq.gz | AP\_phaw\_S4\_longsgl\_01\_A.fastq.gz |
| Sample\_4\_AP\_800bp\_Lane1\_2.fastq.gz | AP\_phaw\_S4\_longsgl\_01\_B.fastq.gz |
| Sample\_4\_AP\_500bp\_Lanes4-5\_1\_A.fastq.gz | AP\_phaw\_S4\_shortsgl\_04\_A.fastq.gz |
| Sample\_4\_AP\_500bp\_Lanes4-5\_1\_B.fastq.gz | AP\_phaw\_S4\_shortsgl\_05\_A.fastq.gz |
| Sample\_4\_AP\_500bp\_Lanes4-5\_2\_A.fastq.gz | AP\_phaw\_S4\_shortsgl\_04\_B.fastq.gz |
| Sample\_4\_AP\_500bp\_Lanes4-5\_2\_B.fastq.gz | AP\_phaw\_S4\_shortsgl\_05\_B.fastq.gz |
| Sample\_4\_AP\_800bp\_Lanes2-3\_1\_A.fastq.gz | AP\_phaw\_S4\_longsgl\_02\_A.fastq.gz |
| Sample\_4\_AP\_800bp\_Lanes2-3\_1\_B.fastq.gz | AP\_phaw\_S4\_longsgl\_03\_A.fastq.gz |
| Sample\_4\_AP\_800bp\_Lanes2-3\_2\_A.fastq.gz | AP\_phaw\_S4\_longsgl\_02\_B.fastq.gz |
| Sample\_4\_AP\_800bp\_Lanes2-3\_2\_B.fastq.gz | AP\_phaw\_S4\_longsgl\_03\_B.fastq.gz |
| Sample\_4\_AP\_20kb\_Lanes1-2\_1\_Asplit.fastq.gz | AP\_phaw\_S4\_20kbmpl\_01\_A.fastq.gz |
| Sample\_4\_AP\_20kb\_Lanes1-2\_1\_Bsplit.fastq.gz | AP\_phaw\_S4\_20kbmpl\_02\_A.fastq.gz |
| Sample\_4\_AP\_20kb\_Lanes1-2\_2\_Asplit.fastq.gz | AP\_phaw\_S4\_20kbmpl\_01\_B.fastq.gz |
| Sample\_4\_AP\_20kb\_Lanes1-2\_2\_Bsplit.fastq.gz | AP\_phaw\_S4\_20kbmpl\_02\_B.fastq.gz |
| Sample\_4\_AP\_20kb\_Lanes3-4\_1\_Asplit.fastq.gz | AP\_phaw\_S4\_20kbmpl\_03\_A.fastq.gz |
| Sample\_4\_AP\_20kb\_Lanes3-4\_1\_Bsplit.fastq.gz | AP\_phaw\_S4\_20kbmpl\_04\_A.fastq.gz |
| Sample\_4\_AP\_20kb\_Lanes3-4\_2\_Asplit.fastq.gz | AP\_phaw\_S4\_20kbmpl\_03\_B.fastq.gz |
| Sample\_4\_AP\_20kb\_Lanes3-4\_2\_Bsplit.fastq.gz | AP\_phaw\_S4\_20kbmpl\_04\_B.fastq.gz |
| Sample\_4\_AP\_6kb\_Lanes1-2\_1\_Asplit.fastq.gz | AP\_phaw\_S4\_6kbmpl\_01\_A.fastq.gz |
| Sample\_4\_AP\_6kb\_Lanes1-2\_1\_Bsplit.fastq.gz | AP\_phaw\_S4\_6kbmpl\_02\_A.fastq.gz |
| Sample\_4\_AP\_6kb\_Lanes1-2\_2\_Asplit.fastq.gz | AP\_phaw\_S4\_6kbmpl\_01\_B.fastq.gz |
| Sample\_4\_AP\_6kb\_Lanes1-2\_2\_Bsplit.fastq.gz | AP\_phaw\_S4\_6kbmpl\_02\_B.fastq.gz |
| Sample\_4\_AP\_6kb\_Lanes3-4\_1\_Asplit.fastq.gz | AP\_phaw\_S4\_6kbmpl\_03\_A.fastq.gz |
| Sample\_4\_AP\_6kb\_Lanes3-4\_1\_Bsplit.fastq.gz | AP\_phaw\_S4\_6kbmpl\_04\_A.fastq.gz |
| Sample\_4\_AP\_6kb\_Lanes3-4\_2\_Asplit.fastq.gz | AP\_phaw\_S4\_6kbmpl\_03\_B.fastq.gz |
| Sample\_4\_AP\_6kb\_Lanes3-4\_2\_Bsplit.fastq.gz | AP\_phaw\_S4\_6kbmpl\_04\_B.fastq.gz |
| Sample\_4\_AP\_8kb\_Lanes1-2\_1\_Asplit.fastq.gz | AP\_phaw\_S4\_8kbmpl\_01\_A.fastq.gz |
| Sample\_4\_AP\_8kb\_Lanes1-2\_1\_Bsplit.fastq.gz | AP\_phaw\_S4\_8kbmpl\_02\_A.fastq.gz |
| Sample\_4\_AP\_8kb\_Lanes1-2\_2\_Asplit.fastq.gz | AP\_phaw\_S4\_8kbmpl\_01\_B.fastq.gz |
| Sample\_4\_AP\_8kb\_Lanes1-2\_2\_Bsplit.fastq.gz | AP\_phaw\_S4\_8kbmpl\_02\_B.fastq.gz |
| Sample\_4\_AP\_8kb\_Lanes3-4\_1\_Asplit.fastq.gz | AP\_phaw\_S4\_8kbmpl\_03\_A.fastq.gz |
| Sample\_4\_AP\_8kb\_Lanes3-4\_1\_Bsplit.fastq.gz | AP\_phaw\_S4\_8kbmpl\_04\_A.fastq.gz |
| Sample\_4\_AP\_8kb\_Lanes3-4\_2\_Asplit.fastq.gz | AP\_phaw\_S4\_8kbmpl\_03\_B.fastq.gz |
| Sample\_4\_AP\_8kb\_Lanes3-4\_2\_Bsplit.fastq.gz | AP\_phaw\_S4\_8kbmpl\_04\_B.fastq.gz |
| Sample\_4\_AP\_9kb\_Lanes1-2\_1\_Asplit.fastq.gz | AP\_phaw\_S4\_9kbmpl\_01\_A.fastq.gz |
| Sample\_4\_AP\_9kb\_Lanes1-2\_1\_Bsplit.fastq.gz | AP\_phaw\_S4\_9kbmpl\_02\_A.fastq.gz |
| Sample\_4\_AP\_9kb\_Lanes1-2\_2\_Asplit.fastq.gz | AP\_phaw\_S4\_9kbmpl\_01\_B.fastq.gz |
| Sample\_4\_AP\_9kb\_Lanes1-2\_2\_Bsplit.fastq.gz | AP\_phaw\_S4\_9kbmpl\_02\_B.fastq.gz |
| Sample\_4\_AP\_9kb\_Lanes3-4\_1\_Asplit.fastq.gz | AP\_phaw\_S4\_9kbmpl\_03\_A.fastq.gz |
| Sample\_4\_AP\_9kb\_Lanes3-4\_1\_Bsplit.fastq.gz | AP\_phaw\_S4\_9kbmpl\_04\_A.fastq.gz |
| Sample\_4\_AP\_9kb\_Lanes3-4\_2\_Asplit.fastq.gz | AP\_phaw\_S4\_9kbmpl\_03\_B.fastq.gz |
| Sample\_4\_AP\_9kb\_Lanes3-4\_2\_Bsplit.fastq.gz | AP\_phaw\_S4\_9kbmpl\_04\_B.fastq.gz |

#### Trimming SGL reads

Trimming at minimum length of 100 and quality of 30 with Trimmomatic

```
parallel -j 4 :::: trim.sh > trim.out 2> trim.err &
```

In [39]:

```
fs = [x for x in os.listdir('/home/share/ngsData/phaw/') if x.split('.')[-1] == 'gz' and x.find('sgl') != -1]

trimCmdPEA = 'java -jar /home/share/software/Trimmomatic-0.32/trimmomatic-0.32.jar PE -threads 6 '
trimCmdB = ' LEADING:__QUAL__ TRAILING:__QUAL__ MINLEN:100 ' + \
'ILLUMINACLIP:/home/share/software/Trimmomatic-0.32/adapters/adapters.fa:2:30:12:1:false TOPHRED33'
trimCmds = ''
inPrefix = '/home/share/ngsData/phaw/'
outPrefix = '/home/share/projects/phaw_genome/trim/'
for lib in list(set(['_'.join(f.split('_')[:-1]) for f in fs])):
    a = lib + '_A'
    b = lib + '_B'
    phred = detectPhred(inPrefix + a + '.fastq.gz', 5000)
    
    if phred == 'Sanger':
        phred = '-phred33 '
    else:
        phred = '-phred64 '
        
    #paired end
    trimCmds += trimCmdPEA + phred + inPrefix + a + '.fastq.gz ' + inPrefix + b + '.fastq.gz ' + \
    outPrefix + a + '.paired.trimmed.fastq.gz ' + outPrefix + a + '.unpaired.trimmed.fastq.gz ' + \
    outPrefix + b + '.paired.trimmed.fastq.gz ' + outPrefix + b + '.unpaired.trimmed.fastq.gz' + trimCmdB + '\n'
qTrimCmds = trimCmds.replace('__QUAL__','30')
HTML(textarea('Trimmomatic bash commands at quality 30 filter',qTrimCmds))
```

Out[39]:

**Trimmomatic bash commands at quality 30 filter**:  

java -jar /home/share/software/Trimmomatic-0.32/trimmomatic-0.32.jar PE -threads 6 -phred33 /home/share/ngsData/phaw/AP\_phaw\_S4\_longsgl\_01\_A.fastq.gz /home/share/ngsData/phaw/AP\_phaw\_S4\_longsgl\_01\_B.fastq.gz /home/share/projects/phaw\_genome/trim/AP\_phaw\_S4\_longsgl\_01\_A.paired.trimmed.fastq.gz /home/share/projects/phaw\_genome/trim/AP\_phaw\_S4\_longsgl\_01\_A.unpaired.trimmed.fastq.gz /home/share/projects/phaw\_genome/trim/AP\_phaw\_S4\_longsgl\_01\_B.paired.trimmed.fastq.gz /home/share/projects/phaw\_genome/trim/AP\_phaw\_S4\_longsgl\_01\_B.unpaired.trimmed.fastq.gz LEADING:30 TRAILING:30 MINLEN:100 ILLUMINACLIP:/home/share/software/Trimmomatic-0.32/adapters/adapters.fa:2:30:12:1:false TOPHRED33
java -jar /home/share/software/Trimmomatic-0.32/trimmomatic-0.32.jar PE -threads 6 -phred33 /home/share/ngsData/phaw/AP\_phaw\_S4\_longsgl\_03\_A.fastq.gz /home/share/ngsData/phaw/AP\_phaw\_S4\_longsgl\_03\_B.fastq.gz /home/share/projects/phaw\_genome/trim/AP\_phaw\_S4\_longsgl\_03\_A.paired.trimmed.fastq.gz /home/share/projects/phaw\_genome/trim/AP\_phaw\_S4\_longsgl\_03\_A.unpaired.trimmed.fastq.gz /home/share/projects/phaw\_genome/trim/AP\_phaw\_S4\_longsgl\_03\_B.paired.trimmed.fastq.gz /home/share/projects/phaw\_genome/trim/AP\_phaw\_S4\_longsgl\_03\_B.unpaired.trimmed.fastq.gz LEADING:30 TRAILING:30 MINLEN:100 ILLUMINACLIP:/home/share/software/Trimmomatic-0.32/adapters/adapters.fa:2:30:12:1:false TOPHRED33
java -jar /home/share/software/Trimmomatic-0.32/trimmomatic-0.32.jar PE -threads 6 -phred33 /home/share/ngsData/phaw/AP\_phaw\_S4\_longsgl\_02\_A.fastq.gz /home/share/ngsData/phaw/AP\_phaw\_S4\_longsgl\_02\_B.fastq.gz /home/share/projects/phaw\_genome/trim/AP\_phaw\_S4\_longsgl\_02\_A.paired.trimmed.fastq.gz /home/share/projects/phaw\_genome/trim/AP\_phaw\_S4\_longsgl\_02\_A.unpaired.trimmed.fastq.gz /home/share/projects/phaw\_genome/trim/AP\_phaw\_S4\_longsgl\_02\_B.paired.trimmed.fastq.gz /home/share/projects/phaw\_genome/trim/AP\_phaw\_S4\_longsgl\_02\_B.unpaired.trimmed.fastq.gz LEADING:30 TRAILING:30 MINLEN:100 ILLUMINACLIP:/home/share/software/Trimmomatic-0.32/adapters/adapters.fa:2:30:12:1:false TOPHRED33
java -jar /home/share/software/Trimmomatic-0.32/trimmomatic-0.32.jar PE -threads 6 -phred33 /home/share/ngsData/phaw/AP\_phaw\_S4\_shortsgl\_01\_A.fastq.gz /home/share/ngsData/phaw/AP\_phaw\_S4\_shortsgl\_01\_B.fastq.gz /home/share/projects/phaw\_genome/trim/AP\_phaw\_S4\_shortsgl\_01\_A.paired.trimmed.fastq.gz /home/share/projects/phaw\_genome/trim/AP\_phaw\_S4\_shortsgl\_01\_A.unpaired.trimmed.fastq.gz /home/share/projects/phaw\_genome/trim/AP\_phaw\_S4\_shortsgl\_01\_B.paired.trimmed.fastq.gz /home/share/projects/phaw\_genome/trim/AP\_phaw\_S4\_shortsgl\_01\_B.unpaired.trimmed.fastq.gz LEADING:30 TRAILING:30 MINLEN:100 ILLUMINACLIP:/home/share/software/Trimmomatic-0.32/adapters/adapters.fa:2:30:12:1:false TOPHRED33
java -jar /home/share/software/Trimmomatic-0.32/trimmomatic-0.32.jar PE -threads 6 -phred33 /home/share/ngsData/phaw/AP\_phaw\_S4\_shortsgl\_02\_A.fastq.gz /home/share/ngsData/phaw/AP\_phaw\_S4\_shortsgl\_02\_B.fastq.gz /home/share/projects/phaw\_genome/trim/AP\_phaw\_S4\_shortsgl\_02\_A.paired.trimmed.fastq.gz /home/share/projects/phaw\_genome/trim/AP\_phaw\_S4\_shortsgl\_02\_A.unpaired.trimmed.fastq.gz /home/share/projects/phaw\_genome/trim/AP\_phaw\_S4\_shortsgl\_02\_B.paired.trimmed.fastq.gz /home/share/projects/phaw\_genome/trim/AP\_phaw\_S4\_shortsgl\_02\_B.unpaired.trimmed.fastq.gz LEADING:30 TRAILING:30 MINLEN:100 ILLUMINACLIP:/home/share/software/Trimmomatic-0.32/adapters/adapters.fa:2:30:12:1:false TOPHRED33
java -jar /home/share/software/Trimmomatic-0.32/trimmomatic-0.32.jar PE -threads 6 -phred33 /home/share/ngsData/phaw/AP\_phaw\_S4\_shortsgl\_03\_A.fastq.gz /home/share/ngsData/phaw/AP\_phaw\_S4\_shortsgl\_03\_B.fastq.gz /home/share/projects/phaw\_genome/trim/AP\_phaw\_S4\_shortsgl\_03\_A.paired.trimmed.fastq.gz /home/share/projects/phaw\_genome/trim/AP\_phaw\_S4\_shortsgl\_03\_A.unpaired.trimmed.fastq.gz /home/share/projects/phaw\_genome/trim/AP\_phaw\_S4\_shortsgl\_03\_B.paired.trimmed.fastq.gz /home/share/projects/phaw\_genome/trim/AP\_phaw\_S4\_shortsgl\_03\_B.unpaired.trimmed.fastq.gz LEADING:30 TRAILING:30 MINLEN:100 ILLUMINACLIP:/home/share/software/Trimmomatic-0.32/adapters/adapters.fa:2:30:12:1:false TOPHRED33
java -jar /home/share/software/Trimmomatic-0.32/trimmomatic-0.32.jar PE -threads 6 -phred33 /home/share/ngsData/phaw/AP\_phaw\_S4\_shortsgl\_04\_A.fastq.gz /home/share/ngsData/phaw/AP\_phaw\_S4\_shortsgl\_04\_B.fastq.gz /home/share/projects/phaw\_genome/trim/AP\_phaw\_S4\_shortsgl\_04\_A.paired.trimmed.fastq.gz /home/share/projects/phaw\_genome/trim/AP\_phaw\_S4\_shortsgl\_04\_A.unpaired.trimmed.fastq.gz /home/share/projects/phaw\_genome/trim/AP\_phaw\_S4\_shortsgl\_04\_B.paired.trimmed.fastq.gz /home/share/projects/phaw\_genome/trim/AP\_phaw\_S4\_shortsgl\_04\_B.unpaired.trimmed.fastq.gz LEADING:30 TRAILING:30 MINLEN:100 ILLUMINACLIP:/home/share/software/Trimmomatic-0.32/adapters/adapters.fa:2:30:12:1:false TOPHRED33
java -jar /home/share/software/Trimmomatic-0.32/trimmomatic-0.32.jar PE -threads 6 -phred33 /home/share/ngsData/phaw/AP\_phaw\_S4\_shortsgl\_05\_A.fastq.gz /home/share/ngsData/phaw/AP\_phaw\_S4\_shortsgl\_05\_B.fastq.gz /home/share/projects/phaw\_genome/trim/AP\_phaw\_S4\_shortsgl\_05\_A.paired.trimmed.fastq.gz /home/share/projects/phaw\_genome/trim/AP\_phaw\_S4\_shortsgl\_05\_A.unpaired.trimmed.fastq.gz /home/share/projects/phaw\_genome/trim/AP\_phaw\_S4\_shortsgl\_05\_B.paired.trimmed.fastq.gz /home/share/projects/phaw\_genome/trim/AP\_phaw\_S4\_shortsgl\_05\_B.unpaired.trimmed.fastq.gz LEADING:30 TRAILING:30 MINLEN:100 ILLUMINACLIP:/home/share/software/Trimmomatic-0.32/adapters/adapters.fa:2:30:12:1:false TOPHRED33
java -jar /home/share/software/Trimmomatic-0.32/trimmomatic-0.32.jar PE -threads 6 -phred33 /home/share/ngsData/phaw/AP\_phaw\_S4\_shortsgl\_06\_A.fastq.gz /home/share/ngsData/phaw/AP\_phaw\_S4\_shortsgl\_06\_B.fastq.gz /home/share/projects/phaw\_genome/trim/AP\_phaw\_S4\_shortsgl\_06\_A.paired.trimmed.fastq.gz /home/share/projects/phaw\_genome/trim/AP\_phaw\_S4\_shortsgl\_06\_A.unpaired.trimmed.fastq.gz /home/share/projects/phaw\_genome/trim/AP\_phaw\_S4\_shortsgl\_06\_B.paired.trimmed.fastq.gz /home/share/projects/phaw\_genome/trim/AP\_phaw\_S4\_shortsgl\_06\_B.unpaired.trimmed.fastq.gz LEADING:30 TRAILING:30 MINLEN:100 ILLUMINACLIP:/home/share/software/Trimmomatic-0.32/adapters/adapters.fa:2:30:12:1:false TOPHRED33
java -jar /home/share/software/Trimmomatic-0.32/trimmomatic-0.32.jar PE -threads 6 -phred33 /home/share/ngsData/phaw/AP\_phaw\_S4\_shortsgl\_07\_A.fastq.gz /home/share/ngsData/phaw/AP\_phaw\_S4\_shortsgl\_07\_B.fastq.gz /home/share/projects/phaw\_genome/trim/AP\_phaw\_S4\_shortsgl\_07\_A.paired.trimmed.fastq.gz /home/share/projects/phaw\_genome/trim/AP\_phaw\_S4\_shortsgl\_07\_A.unpaired.trimmed.fastq.gz /home/share/projects/phaw\_genome/trim/AP\_phaw\_S4\_shortsgl\_07\_B.paired.trimmed.fastq.gz /home/share/projects/phaw\_genome/trim/AP\_phaw\_S4\_shortsgl\_07\_B.unpaired.trimmed.fastq.gz LEADING:30 TRAILING:30 MINLEN:100 ILLUMINACLIP:/home/share/software/Trimmomatic-0.32/adapters/adapters.fa:2:30:12:1:false TOPHRED33
java -jar /home/share/software/Trimmomatic-0.32/trimmomatic-0.32.jar PE -threads 6 -phred33 /home/share/ngsData/phaw/AP\_phaw\_S4\_shortsgl\_08\_A.fastq.gz /home/share/ngsData/phaw/AP\_phaw\_S4\_shortsgl\_08\_B.fastq.gz /home/share/projects/phaw\_genome/trim/AP\_phaw\_S4\_shortsgl\_08\_A.paired.trimmed.fastq.gz /home/share/projects/phaw\_genome/trim/AP\_phaw\_S4\_shortsgl\_08\_A.unpaired.trimmed.fastq.gz /home/share/projects/phaw\_genome/trim/AP\_phaw\_S4\_shortsgl\_08\_B.paired.trimmed.fastq.gz /home/share/projects/phaw\_genome/trim/AP\_phaw\_S4\_shortsgl\_08\_B.unpaired.trimmed.fastq.gz LEADING:30 TRAILING:30 MINLEN:100 ILLUMINACLIP:/home/share/software/Trimmomatic-0.32/adapters/adapters.fa:2:30:12:1:false TOPHRED33
java -jar /home/share/software/Trimmomatic-0.32/trimmomatic-0.32.jar PE -threads 6 -phred33 /home/share/ngsData/phaw/AP\_phaw\_S4\_shortsgl\_09\_A.fastq.gz /home/share/ngsData/phaw/AP\_phaw\_S4\_shortsgl\_09\_B.fastq.gz /home/share/projects/phaw\_genome/trim/AP\_phaw\_S4\_shortsgl\_09\_A.paired.trimmed.fastq.gz /home/share/projects/phaw\_genome/trim/AP\_phaw\_S4\_shortsgl\_09\_A.unpaired.trimmed.fastq.gz /home/share/projects/phaw\_genome/trim/AP\_phaw\_S4\_shortsgl\_09\_B.paired.trimmed.fastq.gz /home/share/projects/phaw\_genome/trim/AP\_phaw\_S4\_shortsgl\_09\_B.unpaired.trimmed.fastq.gz LEADING:30 TRAILING:30 MINLEN:100 ILLUMINACLIP:/home/share/software/Trimmomatic-0.32/adapters/adapters.fa:2:30:12:1:false TOPHRED33

#### Trimming MPL reads with Nextclip

The MPL libraries were made with the Nextera mate-pair library protocol. Here is how the protocol works briefly:

1. DNA is fragmented and a fragment size is selected.
2. Adapters are added to the ends of the fragments

   ```
    -adapter-|-----fragment-----|-adapter-
   ```
3. The fragment is then circularized resulting in the two adapters ligating together.
4. The circularized fragment is fragmented again. The fragment with the merged adapters is selected out.

   ```
    --circularized fragment--|-adapter--adapter-|--circularized fragment--
   ```
5. This fragment with an adapter in the middle is then pair-end sequenced. The resulting pair is in reverse/forward orientation.
6. The adapter in the middle is called a junction adapter. The result of the sequencing can possibly produce an output where:

   ```
    - Both read pairs contain adapter sequences
    - Only one of the read pair contain adapter sequence
    - Both read pairs contain no adapter sequences
   ```

Trimming Nextera adapters can be more complicated than normal trimming because it is possible for the pair-end sequencing to un-evenly sequence both ends resulting in one longer end that contains the entire junction adapter and also the other partial fragment:

```
-----------------------reverse read------------->
--circularized fragment--|-adapter--adapter-|--circularized fragment--
                                                 <--forward read------
```

This would result in a sequence where there is a junction adapter sequence inserted in the read.

The Nextclip program is used to clip Nextera MPL libraries. For cases where the read contains the entire junction adapter and partial fragment, the read will be clipped to the beginning of the junction adapter. Nextclip outputs 4 classes of reads:

```
    A. Both read pairs contain adapter
    B. Read 1 contain adapter
    C. read 2 contain adapter
    D. Both read pairs don't contain adapter
```

The Nextclip manual suggest to discard class D reads as they could come from circularized fragments without adapters, in which case the insert-size would be unknown or really short.

For contig assembly all 4 classes of clipped reads will be used as we don't care about the fragment size in contig assembly. For scaffolding, only class A, B, C, and D will be used.

The command used (minimum read length of 50 and remove PCR duplicates):

```
nextclip -d -m 50 -i A.fastq -j B.fastq -o libName
```

The following table presents stats on the Nextclip output:

In [40]:

```
clipStat = open(prefix + 'reads/clip.stat').read().strip()
blocks = clipStat.split('NextClip v1.3.1')[1:]
clipTable = ListTable()
clipTable.append(['Lib',\
                  'Total Pairs',\
                  'Duplicate Pairs',\
                  'Too Short',\
                  'Reads with N',\
                  'Cat. A','Cat. B',\
                  'Cat. C','Cat. D',\
                  'A + B + C','A + B + C + D'])
for block in blocks:
    lines = [line for line in block.strip().split('\n') if line != '']
    lib = ''
    for line in lines:
        if line != line.split()[0] == "Opening":
            lib = line.strip().split()[-1].split('/')[-1].split('.')[0][:-2]
            break
    d = block.strip().split('SUMMARY')
    if len(d) == 2:
        data = dict([x.strip().split(": ") for x in d[1].split('\n')[:-1] if x != ''])
        clipTable.append([lib,data['Number of read pairs']\
                          ,data['Number of duplicate pairs'].split()[1]\
                          ,data['All categories too short'].split()[1]\
                          ,data['Number of pairs containing N'].split()[1]\
                          ,data['A pairs long enough'].split()[1]\
                          ,data['B pairs long enough'].split()[1]\
                          ,data['C pairs long enough'].split()[1]\
                          ,data['D pairs long enough'].split()[1]\
                          ,data['Total usable pairs'].split()[1]\
                          ,data['All long enough'].split()[1]])
clipTable
```

Out[40]:

|  |  |  |  |  |  |  |  |  |  |  |
| --- | --- | --- | --- | --- | --- | --- | --- | --- | --- | --- |
| Lib | Total Pairs | Duplicate Pairs | Too Short | Reads with N | Cat. A | Cat. B | Cat. C | Cat. D | A + B + C | A + B + C + D |
| AP\_phaw\_S4\_20kbmpl\_01 | 20680714 | 19.66 | 34.54 | 0.94 | 6.09 | 13.55 | 13.84 | 12.32 | 33.47 | 45.80 |
| AP\_phaw\_S4\_20kbmpl\_03 | 21446730 | 21.27 | 34.15 | 0.29 | 6.12 | 13.33 | 13.51 | 11.62 | 32.96 | 44.58 |
| AP\_phaw\_S4\_20kbmpl\_02 | 21616040 | 21.30 | 33.98 | 0.71 | 6.06 | 13.29 | 13.53 | 11.84 | 32.89 | 44.72 |
| AP\_phaw\_S4\_20kbmpl\_04 | 21860749 | 21.62 | 34.02 | 0.24 | 6.12 | 13.26 | 13.45 | 11.53 | 32.83 | 44.36 |
| AP\_phaw\_S4\_9kbmpl\_01 | 21086124 | 3.78 | 35.15 | 0.94 | 3.92 | 17.03 | 17.24 | 22.88 | 38.19 | 61.07 |
| AP\_phaw\_S4\_9kbmpl\_02 | 22118658 | 4.12 | 35.08 | 0.71 | 3.92 | 17.02 | 17.20 | 22.66 | 38.13 | 60.79 |
| AP\_phaw\_S4\_9kbmpl\_03 | 22079636 | 4.15 | 35.30 | 0.28 | 3.98 | 17.12 | 17.24 | 22.22 | 38.33 | 60.55 |
| AP\_phaw\_S4\_9kbmpl\_04 | 22575306 | 4.26 | 35.28 | 0.23 | 3.99 | 17.12 | 17.22 | 22.13 | 38.33 | 60.46 |
| AP\_phaw\_S4\_8kbmpl\_01 | 24581417 | 3.91 | 37.35 | 0.93 | 4.95 | 16.70 | 16.98 | 20.11 | 38.64 | 58.75 |
| AP\_phaw\_S4\_8kbmpl\_02 | 25676712 | 4.27 | 37.27 | 0.70 | 4.97 | 16.69 | 16.96 | 19.84 | 38.62 | 58.46 |
| AP\_phaw\_S4\_8kbmpl\_03 | 26227593 | 4.35 | 37.43 | 0.29 | 5.02 | 16.77 | 16.96 | 19.46 | 38.75 | 58.21 |
| AP\_phaw\_S4\_8kbmpl\_04 | 26965558 | 4.49 | 37.39 | 0.24 | 5.03 | 16.75 | 16.95 | 19.39 | 38.73 | 58.12 |
| AP\_phaw\_S4\_6kbmpl\_01 | 32936129 | 3.31 | 46.19 | 0.94 | 8.54 | 14.65 | 14.93 | 12.38 | 38.11 | 50.50 |
| AP\_phaw\_S4\_6kbmpl\_02 | 34325108 | 3.64 | 46.12 | 0.71 | 8.58 | 14.66 | 14.90 | 12.10 | 38.15 | 50.25 |
| AP\_phaw\_S4\_6kbmpl\_03 | 34791180 | 3.66 | 46.27 | 0.30 | 8.65 | 14.72 | 14.87 | 11.82 | 38.24 | 50.07 |
| AP\_phaw\_S4\_6kbmpl\_04 | 35547676 | 3.75 | 46.25 | 0.24 | 8.65 | 14.71 | 14.85 | 11.79 | 38.21 | 50.00 |

In [41]:

```
readStat = open(prefix + 'reads/reads.count')
rawShortBases = sum([int(x.split('\t')[1]) for x in open(prefix + 'reads/raw.baseCount').read().strip().split('\n')\
            if x.split('\t')[0].find('shortsgl') != -1])
rawLongBases = sum([int(x.split('\t')[1]) for x in open(prefix + 'reads/raw.baseCount').read().strip().split('\n')\
            if x.split('\t')[0].find('longsgl') != -1])
rawMPLBases = sum([int(x.split('\t')[1]) for x in open(prefix + 'reads/raw.baseCount').read().strip().split('\n')\
            if x.split('\t')[0].find('kbmpl') != -1])

shortCount = [0,0]
longCount = [0,0]
mplCount = [0,0]
for line in readStat:
    meta = line.strip().split()
    if meta[0].find('mpl') != -1:
        mplCount[0] += int(meta[1])
        mplCount[1] += int(meta[2])
    elif meta[0].find('shortsgl') != -1:
        shortCount[0] += int(meta[1])
        shortCount[1] += int(meta[2])
    elif meta[0].find('longsgl') != -1:
        longCount[0] += int(meta[1])
        longCount[1] += int(meta[2])
print 'Read stats after trimming/clipping'
totalTable = ListTable()
totalTable.append(['Library Type',\
                   '# bases before trimming',\
                   '# bases after trimming/clipping',\
                   'Average trimmed/clipped read length'])
totalTable.append(['Short-SGL',\
                   commas(rawShortBases),\
                   commas(shortCount[0]),\
                   float(shortCount[0] / float(shortCount[1]))])
totalTable.append(['Long-SGL',\
                   commas(rawLongBases),\
                   commas(longCount[0]),\
                   float(longCount[0] / float(longCount[1]))])
totalTable.append(['MPL',\
                   commas(rawMPLBases),\
                   commas(mplCount[0]),\
                   float(mplCount[0] / float(mplCount[1]))])
totalTable
```

```
Read stats after trimming/clipping
```

Out[41]:

|  |  |  |  |
| --- | --- | --- | --- |
| Library Type | # bases before trimming | # bases after trimming/clipping | Average trimmed/clipped read length |
| Short-SGL | 286,840,902,720 | 266,007,897,423 | 148.604036204 |
| Long-SGL | 112,763,372,370 | 98,629,114,244 | 147.37445096 |
| MPL | 124,354,599,000 | 50,270,735,451 | 113.631330157 |

In [42]:

```
print 'Total bases:', commas(shortCount[0] + longCount[0] + mplCount[0])
print 'Estimated genome size: 3.6gb'
print 'Raw coverage:', (shortCount[0] + longCount[0] + mplCount[0]) / 3600000000
```

```
Total bases: 414,907,747,118
Estimated genome size: 3.6gb
Raw coverage: 115
```

### K-mer analysis

The following figures show k-mer spectra for short-sgl, long-sgl, mpls and all data together. "Good" k-mers are defined here as k-mers above the first coverage minima. K-mers below the first coverage minima are most likely sequencing errors which will probably be discarded by the assembler.

The intersection of the unique good k-mers of the three datasets shows high overlap, meaning the sequence information content among the three library types are consistent with each other. However, this does not show potential coverage bias among the three library types.

#### K-mer spectra of Short-SGL, Long-SGL, and MPL

In [8]:

```
shortData = [(int(line.strip().split()[0]), int(line.strip().split()[1])) \
             for line in open(prefix + 'spectra/shortsgl.30.histo')]
longData = [(int(line.strip().split()[0]), int(line.strip().split()[1])) \
            for line in open(prefix + 'spectra/longsgl.30.histo')]
mplData = [(int(line.strip().split()[0]), int(line.strip().split()[1])) \
           for line in open(prefix + 'spectra/mpl.30.histo')]
bbnormData = [(int(line.strip().split()[0]), int(line.strip().split()[1])) \
              for line in open(prefix + 'spectra/bbnorm.30.histo')]
allData = [(int(line.strip().split()[0]), int(line.strip().split()[1])) \
           for line in open(prefix + 'spectra/all.30.histo')]

pd.Series([x[1] for x in shortData], index = [x[0] for x in shortData])[:150].plot(kind='area', alpha=0.3,\
                                                                                   figsize=[15,6], ylim=[0,2e8])
pd.Series([x[1] for x in longData], index = [x[0] for x in longData])[:150].plot(kind='area', alpha=0.3,\
                                                                                 figsize=[15,6], ylim=[0,2e8])
pd.Series([x[1] for x in mplData], index = [x[0] for x in mplData])[:150].plot(kind='area', alpha=0.3,\
                                                                               figsize=[15,6], ylim=[0,2e8])
pd.Series([x[1] for x in allData], index = [x[0] for x in allData])[:150].plot(kind='area', alpha=0.3,\
                                                                                     figsize=[15,6], ylim=[0,2e8])

plt.legend(['shortSGL','longSGL','MPL','All data'])
```

Out[8]:

```
<matplotlib.legend.Legend at 0x18f58c50>
```

#### K-mer spectra of all reads

In [10]:

```
pd.Series([x[1] for x in allData], index = [x[0] for x in allData])[:200].plot(kind='area', alpha=0.3,\
                                                                                     figsize=[15,6], ylim=[0,0.35e8])
```

Out[10]:

```
<matplotlib.axes.AxesSubplot at 0x6b3e810>
```

#### Genome statistics based on k-mer analysis

In [157]:

```
def getMetric(path):
    histoData = [(int(line.strip().split()[0]), int(line.strip().split()[1])) for line in open(path)]
    s = pd.Series([x[1] for x in histoData], index = [x[0] for x in histoData])
    lowCov = 0
    avgCov = 0
    for i, v in enumerate(histoData):
        if histoData[i + 1][1] > histoData[i][1]:
            lowCov = histoData[i][0]
            break
            
    for i, v in enumerate(histoData[lowCov:]):
        if histoData[lowCov + i + 1][1] < histoData[lowCov + i][1]:
            avgCov = histoData[lowCov + i][0]
            break

    k = s * s.index
    
    #mean read length - k-length + 1 / mean read length to normalize coverage
    #assume mean read length is 145.
    #145 - 30 + 1 / 145 = 0.8
    return {'mincov':lowCov,\
            'peakcov':avgCov,\
            'uniqk':commas(int(s.sum())),\
            'allk':commas(int(k.sum())),\
            'erroruk':commas(int(s[:lowCov].sum())),\
            'realuk':commas(int(s[lowCov:].sum())),\
            'errork':commas(int(k[:lowCov].sum())),\
            'realk':commas(int(k[lowCov:].sum())),\
            'error':float(sum(k[:lowCov])) / sum(k) / 30,\
            'size':int(k[lowCov:].sum() / avgCov)}
```

In [273]:

```
shortData = getMetric(prefix + 'spectra/shortsgl.30.histo')
longData = getMetric(prefix + 'spectra/longsgl.30.histo')
mplData = getMetric(prefix + 'spectra/mpl.30.histo')
allData = getMetric(prefix + 'spectra/all.30.histo')
allData['peakcov'] = 57
allData['size'] = int(allData['realk'].replace(',','')) / 57
bbData = getMetric(prefix + 'spectra/bbnorm.30.histo')


metricTable = ListTable()
metricTable.append(['Library','error coverage','peak coverage','all k-mers',\
                    'unique k-mers','error k-mers','good k-mers','error unique k-mers',\
                    'good unique k-mers'])
names =['shortSGL','longSGL','MPL','all','bbNorm']

for i,data in enumerate([(shortData,(147 - 30 + 1) / 147.0), \
                         (longData,(147 - 30 + 1) / 147.0), \
                         (mplData,(114 - 30 + 1) / 114.0), \
                         (allData,(130 - 30 + 1) / 130.0)]):

    d = data[0]
    s = commas(int(data[1] * d['size']))
    metricTable.append([names[i],\
                        d['mincov'],\
                        d['peakcov'],\
                        d['allk'],\
                        d['uniqk'],\
                        d['errork'],\
                        d['realk'],\
                        d['erroruk'],\
                        d['realuk']])
metricTable
```

Out[273]:

|  |  |  |  |  |  |  |  |  |
| --- | --- | --- | --- | --- | --- | --- | --- | --- |
| Library | error coverage | peak coverage | all k-mers | unique k-mers | error k-mers | good k-mers | error unique k-mers | good unique k-mers |
| shortSGL | 9 | 37 | 180,742,689,259 | 3,689,894,516 | 1,881,378,027 | 178,861,311,232 | 1,438,248,827 | 2,251,645,689 |
| longSGL | 3 | 10 | 67,729,982,093 | 2,755,129,156 | 830,621,908 | 66,899,360,185 | 617,342,497 | 2,137,786,659 |
| MPL | 2 | 6 | 34,868,732,907 | 3,450,297,218 | 1,452,330,509 | 33,416,402,398 | 1,331,483,022 | 2,118,814,196 |
| all | 15 | 57 | 270,933,539,388 | 5,230,408,736 | 3,886,617,726 | 267,046,921,662 | 2,974,163,059 | 2,256,245,677 |

In [276]:

```
histoData = [(int(line.strip().split()[0]), int(line.strip().split()[1])) for line in open(prefix + 'spectra/all.30.histo')]
s = pd.Series([x[1] for x in histoData], index = [x[0] for x in histoData])
k = s * s.index
print 'estimated genome size not taking into account heterozygosity:',\
commas(int(k[15:101].sum() / 57  + k[100:].sum() / 114))
print 'estimated genome size taking into account heterozygosity:', \
commas(int(k[15:101].sum() / 57 / 2 + k[100:].sum() / 114))
```

```
estimated genome size not taking into account heterozygosity: 3,186,681,201
estimated genome size taking into account heterozygosity: 2,353,869,063
```

#### Intersection of good k-mers among the three library types

In [54]:

```
from matplotlib_venn import venn3, venn2
'''
A = shortsgl
B = longsgl
C = mpl

Abc = 9142176
aBc = 35819919
ABc = 59846436
abC = 43811369
AbC = 88180851
aBC = 8740025
ABC = 2098929438
'''
plt.figure(figsize=(6,6))
venn3(subsets = (9142176, 35819919, 59846436, 43811369, 88180851, 8740025, 2098929438), \
      set_labels = ('Short-SGL', 'Long-SGL', 'MPL'))
```

Out[54]:

```
<matplotlib_venn._common.VennDiagram instance at 0x228907a0>
```

In [55]:

```
setTable = ListTable()
setTable.append(['library','good unique k-mers','percent in intersection'])
setTable.append(['Short-SGL',2256098901,100 * float(2098929438) / 2256098901])
setTable.append(['Long-SGL',2203335818,100 * float(2098929438) / 2203335818])
setTable.append(['MPL',2239661683,100 * float(2098929438) / 2239661683])
setTable
```

Out[55]:

|  |  |  |
| --- | --- | --- |
| library | good unique k-mers | percent in intersection |
| Short-SGL | 2256098901 | 93.0335738859 |
| Long-SGL | 2203335818 | 95.2614404419 |
| MPL | 2239661683 | 93.7163614456 |

### Contig assembly

Contig assembly was performed using Abyss on Amazon cloud instances. StarCluster was used to automatically setup a cluster environment (Sun-grid engine).

#### AWS and Abyss setup

The following are setup instructions for setting a cluster of 21 nodes (small head node + 20 high CPU nodes) and running Abyss.

Install StarCluster by:

```
sudo pip install starcluster
```

The following config was used (~/.starcluster/config) to start up 20 c3.8xlarge (32 CPUs, 60 gb RAM each) instances. Master node is a m3.xlarge.

```
[global]
DEFAULT_TEMPLATE=abyss

[aws info]
AWS_ACCESS_KEY_ID = KEY
AWS_SECRET_ACCESS_KEY = KEY
AWS_USER_ID= ID
AWS_REGION_NAME = eu-west-1
AWS_REGION_HOST = ec2.eu-west-1.amazonaws.com

[key dk]
KEY_LOCATION=~/.ssh/dk.pem

[cluster abyss]
KEYNAME = dk
CLUSTER_SIZE = 21
CLUSTER_USER = sgeadmin
CLUSTER_SHELL = bash
NODE_IMAGE_ID = ami-ca4abfbd
NODE_INSTANCE_TYPE = c3.8xlarge
MASTER_INSTANCE_TYPE = m3.xlarge
MASTER_IMAGE_ID = ami-ca4abfbd
AVAILABILITY_ZONE = eu-west-1a
VOLUMES = data
SPOT_BID = 3.0

[volume data]
VOLUME_ID = vol-f0daa6f7
MOUNT_PATH = /working
```

Important things to note about StarCluster:

- The bid price can't go above the maximum average price of the instance. StarCluster will throw an error.
- Can't use the standard Amazon Ubuntu AMI. The NFS setup doesn't seem to work. Find a StarCluster AMI in your zone and use that.
- The key-pair name has to be the same as the name in AWS.
- There is a maximum limit on AWS spot instances. 20 is the limit for c3.8xlarge instances. It is possible to raise this limit by contacting AWS.

To start the cluster:

```
starcluster start abyss
```

To ssh into cluster as root:

```
starcluster sshmaster abyss
```

To terminate cluster:

```
starcluster terminate abyss
```

To install abyss:

```
sudo apt-get install -y sparsehash
wget http://sourceforge.net/projects/boost/files/boost/1.56.0/boost_1_56_0.zip/download
mv download boost.zip
unzip boost.zip
git clone https://github.com/bcgsc/abyss.git
cd abyss
./autogen.sh
./configure --enable-maxk=128 --prefix=INSTALL_PATH --with-boost=/root/boost_1_56_0/ --with-mpi=/usr/lib/openmpi
```

Add abyss to the `PATH` variable:

```
echo 'PATH=$PATH:/working/software/bin' >> ~/.bashrc
source ~/.bashrc
```

To get the Abyss-pe run commands, use the `--dry-run` parameter:

```
/working/software/bin/abyss-pe k=70 np=640 j=32 q=30 lib='shortsgl longsgl' shortsgl='/working/reads/AP_phaw_S4_shortsgl_*' longsgl='/working/reads/AP_phaw_S4_longsgl_*' name='pilot' --dry-run > abyss.commands
```

- `k` specifies k-mer
- `np` specifies process. Number of nodes \* number of cpus per node
- `j` specifies number of cpus per node
- `q` quality trimming

Take the `mpirun` line from the dry run and make a `abyss.mpi.sh` file. Make it executable:

```
chmod a+x abyss.mpi.sh
```

Qsub the command:

```
qsub -V -cwd -pe orte 640 ./abyss.mpi.sh
```

#### Assemblies

2 different k-mer assemblies (k120 and k70) were peformed with all the reads.

In addition, merging of the two genome assemblies was also performed with GAM-NGS. GAM-NGS works by designating a master and slave assembly. The slave assembly is then used to merge master assembly's contigs. All master assembly contigs will be outputted; however slave contigs not found in the master asembly is not used and also not outputted. The k120 assembly was merged to the k70 assembly.

The GAM-NGS merging was done by first mapping short-sgl reads as PE to each contig assembly:

```
bowtie2 -X 600 --no-unal --no-mixed --no-discordant --no-dovetail --no-contain --no-overlap -p 32 -x indexFile -1 pairA -2 pairB | samtools view -Shb - > output.bam
parallel samtools sort -m 5G -O bam -T {.} -@ 32 {} \> {.}.sorted.bam ::: *.bam
parallel samtools index {} ::: *.sorted.bam
```

An info file is created for each contig assembly listing where the .bam file are and estimated insert sizes:

Then gam-create and gam-merge are used with the info files:

```
gam-create --master-bam /phaw/gam/bam/k120/k120.info --slave-bam /phaw/gam/bam/k70/k70.info --min-block-size 10 --output k120_k70
gam-merge  --master-bam /phaw/gam/bam/k120/k120.info --slave-bam /phaw/gam/bam/k70/k70.info --min-block-size 50 --blocks-file k120_k70.blocks --master-fasta abyss_k120.ctg.renamed.filtered.fa --slave-fasta abyss_k70.ctg.renamed.filtered.fa --threads 32 --output k120_k70.gam 2> merge.err
```

#### Assembly stats

##### K-mer coverage

Jellyfish was ran on k120 and k70 assemblies. Using the k-mer count output, we can calculate k-mer coverage for each contig sequence. If a contig is totally unique, we should see a constant k-mer coverage of 1 throughout the contig. Areas of high k-mer coverage represent regions that appear in many other places in the contig assembly (repetitive regions). We expect contigs with high k-mer coverage to be representative of regions of high read coverage.

The following figure plots the length of each contig vs the average coverage across the contig. K-mer coverage is capped at 100 making the y-axis bounded from 1 to 100. We notice artifacts in coverage for k-mers at k  *2 length. This is likely due to the assembler designating filtering thresholds at k*  2.

##### K120 assembly

In [55]:

```
from Bio import SeqIO
from collections import defaultdict
import math

def nX(lengths):
    bpCount = 0
    totalCount = sum(lengths)
    nx = {}

    for l in lengths:
        bpCount += l
        xPercent = math.floor(float(bpCount) / totalCount * 100)
        
        if not nx.has_key(xPercent):
            nx[xPercent] = l
            
    nx = nx.items()
    nx.sort(key = lambda x : x[0])
    return nx
```

In [69]:

```
abyss_k120_stat = pd.read_csv(open(prefix + 'kmer/abyss_k120.ctg.renamed.covStat'),sep='\t',index_col=0)
abyss_k120_stat.columns = ['min','max','avg','std','length']

import numpy as np
abyss_k120_sample = abyss_k120_stat.loc[np.random.choice(abyss_k120_stat.index, 100000, replace=False)]
```

In [72]:

```
print 'Bounded 1-100 abyss k120 assembly (subsampled 100000) length by k-mer coverage plot'
fig, axes = plt.subplots(ncols = 2, nrows = 1, figsize=[18,5])
abyss_k120_sample[abyss_k120_sample['length'] < 1000].plot(ax=axes[0],kind='scatter', x='length',y='avg',alpha=0.1, s = 2)
abyss_k120_sample['avg'].hist(bins = 100, ax = axes[1])
axes[0].set_title('length vs average coverage')
axes[1].set_title('histogram of avg coverage')
```

```
Bounded 1-100 abyss k120 assembly (subsampled 100000) length by k-mer coverage plot
```

Out[72]:

```
<matplotlib.text.Text at 0x2f9106d0>
```

Given an average k-mer coverage of ~50 across a 100bp contig. This can possibly represent a constant coverage of 50 across the entire contig. Or this can mean a coverage of 1 across half of the contig and coverage of 100 across the other half.

We can calculate the potential maximum number of bases with "good" coverage (We define good coverage as 3 or less) by:

```
max low coverage (MLC) length = length - (Length * avg coverage / 97)
```

Contigs with short MLC length can be filtered out because they are mostly repetitive. Most of the raw reads are ~140 bp in length, so we can filter out any contigs with MLC length of less than 140.

In [75]:

```
abyss_k120_sample_mlc = abyss_k120_sample['length'] - (abyss_k120_sample['length'] * abyss_k120_sample['avg'] / 97)
abyss_k120_sample_lengthFilter = abyss_k120_sample[abyss_k120_sample['length'] > 239]
abyss_k120_sample_length_mclFilter = abyss_k120_sample\
            [~abyss_k120_sample.index.isin(abyss_k120_sample_mlc[abyss_k120_sample_mlc < 140].index)]\
            [abyss_k120_sample['length'] > 239]
```

In [74]:

```
fig, axes = plt.subplots(ncols = 2, nrows = 1, figsize=[18,5])

ax = abyss_k120_sample\
        [~abyss_k120_sample.index.isin(abyss_k120_sample_length_mclFilter.index)]\
        [abyss_k120_sample['length'] < 1000]\
            .plot(ax = axes[1], color=sns.color_palette()[0], kind='scatter', x='length',y='avg',alpha=0.1, s = 2)
            
abyss_k120_sample_lengthFilter\
        [abyss_k120_sample_lengthFilter['length'] < 1000]\
            .plot(color=sns.color_palette()[1],ax = ax, kind='scatter', x='length',y='avg',alpha=0.1, s = 2)
abyss_k120_sample_length_mclFilter\
        [abyss_k120_sample_length_mclFilter['length'] < 1000]\
            .plot(color=sns.color_palette()[2],ax = ax, kind='scatter', x='length',y='avg',alpha=0.1, s = 2)

abyss_k120_sample['avg'].hist(ax = axes[0], bins = 100).set_ylim([0,9000])
abyss_k120_sample_lengthFilter['avg'].hist(ax = axes[0], bins = 100)
abyss_k120_sample_length_mclFilter['avg'].hist(ax = axes[0], bins = 100)
axes[0].legend(['all','length > 239','length > 239 and MCL length >= 140'])
```

Out[74]:

```
<matplotlib.legend.Legend at 0x27387b10>
```

In [76]:

```
abyss_k120_mlc = abyss_k120_stat['length'] - (abyss_k120_stat['length'] * abyss_k120_stat['avg'] / 97)
abyss_k120_lengths = abyss_k120_stat\
        [~abyss_k120_stat.index.isin(abyss_k120_mlc[abyss_k120_mlc < 140].index)]\
        [abyss_k120_stat['length'] > 239]['length'].copy()
abyss_k120_lengths.sort(ascending = False)
abyss_k120_nx = nX(abyss_k120_lengths)

print 'Total number of contigs:', commas(len(abyss_k120_stat))
print 'Total bases:', commas(int(sum(abyss_k120_stat['length'])))
print
print '# contigs after filtering length > 239:', \
commas(len(abyss_k120_stat[abyss_k120_stat['length'] > 239]))
print '# bases after filtering length > 239:', \
commas(int(sum((abyss_k120_stat[abyss_k120_stat['length'] > 239]['length']))))
print '# contigs after filtering MCL length >= 140:', \
commas(len(abyss_k120_stat\
           [~abyss_k120_stat.index.isin(abyss_k120_mlc[abyss_k120_mlc < 140].index)]\
           [abyss_k120_stat['length'] > 239]))
print '# bases after filtering MCL length >= 140:', \
commas(int(sum(abyss_k120_stat\
               [~abyss_k120_stat.index.isin(abyss_k120_mlc[abyss_k120_mlc < 140].index)]\
               [abyss_k120_stat['length'] > 239]['length'])))
print
for i in range(10):
    print 'N' + str(i * 10) + ':', commas(int(abyss_k120_nx[i * 10][1]))
```

```
Total number of contigs: 8,262,605
Total bases: 4,002,316,143

# contigs after filtering length > 239: 2,914,040
# bases after filtering length > 239: 3,146,121,733
# contigs after filtering MCL length >= 140: 2,362,466
# bases after filtering MCL length >= 140: 2,985,551,621

N0: 93,260
N10: 9,817
N20: 5,988
N30: 4,262
N40: 3,184
N50: 2,392
N60: 1,765
N70: 1,244
N80: 799
N90: 453
```

##### K70 assembly

In [77]:

```
abyss_k70_stat = pd.read_csv(open(prefix + 'kmer/abyss_k70.ctg.renamed.covStat'),sep='\t',index_col=0)
abyss_k70_stat.columns = ['min','max','avg','std','length']

import numpy as np
abyss_k70_sample = abyss_k70_stat.loc[np.random.choice(abyss_k70_stat.index, 100000, replace=False)]
```

In [82]:

```
print 'Bounded 1-100 abyss k70 assembly (subsampled 100000) length by k-mer coverage plot'
fig, axes = plt.subplots(ncols = 2, nrows = 1, figsize=[18,5])
abyss_k70_sample[abyss_k70_sample['length'] < 1000].plot(ax=axes[0],kind='scatter', x='length',y='avg',alpha=0.1, s = 2)
abyss_k70_sample['avg'].hist(bins = 100, ax = axes[1])
axes[0].set_title('length vs average coverage')
axes[1].set_title('histogram of avg coverage')
```

```
Bounded 1-100 abyss k70 assembly (subsampled 100000) length by k-mer coverage plot
```

Out[82]:

```
<matplotlib.text.Text at 0x5c718750>
```

In [79]:

```
abyss_k70_sample_mlc = abyss_k70_sample['length'] - (abyss_k70_sample['length'] * abyss_k70_sample['avg'] / 97)
abyss_k70_sample_lengthFilter = abyss_k70_sample[abyss_k70_sample['length'] > 139]
abyss_k70_sample_length_mclFilter = abyss_k70_sample\
    [~abyss_k70_sample.index.isin(abyss_k70_sample_mlc[abyss_k70_sample_mlc < 140].index)]\
    [abyss_k70_sample['length'] > 139]
```

In [83]:

```
fig, axes = plt.subplots(ncols = 2, nrows = 1, figsize=[18,5])

ax = abyss_k70_sample\
    [~abyss_k70_sample.index.isin(abyss_k70_sample_length_mclFilter.index)]\
    [abyss_k70_sample['length'] < 1000]\
        .plot(ax = axes[1], color=sns.color_palette()[0], kind='scatter', x='length',y='avg',alpha=0.1, s = 2)
abyss_k70_sample_lengthFilter\
    [abyss_k70_sample_lengthFilter['length'] < 1000]\
        .plot(color=sns.color_palette()[1],ax = ax, kind='scatter', x='length',y='avg',alpha=0.1, s = 2)
abyss_k70_sample_length_mclFilter\
    [abyss_k70_sample_length_mclFilter['length'] < 1000]\
        .plot(color=sns.color_palette()[2],ax = ax, kind='scatter', x='length',y='avg',alpha=0.1, s = 2)

abyss_k70_sample['avg'].hist(ax = axes[0], bins = 100).set_ylim([0,9000])
abyss_k70_sample_lengthFilter['avg'].hist(ax = axes[0], bins = 100)
abyss_k70_sample_length_mclFilter['avg'].hist(ax = axes[0], bins = 100)
axes[0].legend(['all','length > 239','length > 139 and MCL length >= 140'])
```

Out[83]:

```
<matplotlib.legend.Legend at 0x570e6e50>
```

In [81]:

```
abyss_k70_mlc = abyss_k70_stat['length'] - (abyss_k70_stat['length'] * abyss_k70_stat['avg'] / 97)
abyss_k70_lengths = abyss_k70_stat\
    [~abyss_k70_stat.index.isin(abyss_k70_mlc[abyss_k70_mlc < 140].index)]\
    [abyss_k70_stat['length'] > 139]['length'].copy()
abyss_k70_lengths.sort(ascending = False)
abyss_k70_nx = nX(abyss_k70_lengths)

print 'Total number of contigs:', commas(len(abyss_k70_stat))
print 'Total bases:', commas(int(sum(abyss_k70_stat['length'])))
print
print '# contigs after filtering length > 139:', \
commas(len(abyss_k70_stat[abyss_k70_stat['length'] > 139]))
print '# bases after filtering length > 139:', \
commas(int(sum((abyss_k70_stat[abyss_k70_stat['length'] > 139]['length']))))
print '# contigs after filtering MCL length >= 140:', \
commas(len(abyss_k70_stat\
           [~abyss_k70_stat.index.isin(abyss_k70_mlc[abyss_k70_mlc < 140].index)]\
           [abyss_k70_stat['length'] > 139]))
print '# bases after filtering MCL length >= 140:', \
commas(int(sum(abyss_k70_stat\
               [~abyss_k70_stat.index.isin(abyss_k70_mlc[abyss_k70_mlc < 140].index)]\
               [abyss_k70_stat['length'] > 139]['length'])))
print
for i in range(10):
    print 'N' + str(i * 10) + ':', commas(int(abyss_k70_nx[i * 10][1]))
```

```
Total number of contigs: 18,580,329
Total bases: 3,907,388,890

# contigs after filtering length > 139: 4,639,709
# bases after filtering length > 139: 2,633,402,885
# contigs after filtering MCL length >= 140: 3,042,142
# bases after filtering MCL length >= 140: 2,364,415,013

N0: 62,709
N10: 7,003
N20: 4,263
N30: 2,956
N40: 2,143
N50: 1,562
N60: 1,111
N70: 742
N80: 446
N90: 269
```

##### Length stats after filtering

Summary of the k120, k70 and merged K120 + k70 assemblies:

In [7]:

```
fs = [x for x in os.listdir(prefix + 'contig') if x.split('.')[-1] == 'length']
lengths = {}
nxs = {}
for f in fs:
    libLength = [int(x.split()[1]) for x in open(prefix + 'contig/' + f).read().strip().split('\n')]
    libLength.sort(reverse = True)
    libName = ' '.join(f.split('.')[0].split('_'))
    
    lengths[libName] = libLength
    nxs[libName] = nX(libLength)
libs = lengths.keys()
indLibs = ['abyss k120','abyss k70','k120 k70']
summaryTable = ListTable()
summaryTable.append([''] + indLibs)
summaryTable.append(['#contigs'] + [commas(len(lengths[lib])) for lib in indLibs])
summaryTable.append(['#max length'] + [str(lengths[lib][0]) for lib in indLibs])
summaryTable.append(['#sum bases'] + [commas(sum(lengths[lib])) for lib in indLibs])
for i in range(10):
    summaryTable.append(['N' + str(i * 10)] + [str(nxs[lib][i * 10][1]) for lib in indLibs])
summaryTable
```

Out[7]:

|  |  |  |  |
| --- | --- | --- | --- |
|  | abyss k120 | abyss k70 | k120 k70 |
| #contigs | 2,362,466 | 3,042,142 | 2,294,695 |
| #max length | 93260 | 62709 | 93260 |
| #sum bases | 2,985,551,621 | 2,364,415,013 | 2,981,518,945 |
| N0 | 93260 | 62709 | 93260 |
| N10 | 9817 | 7003 | 10625 |
| N20 | 5988 | 4263 | 6432 |
| N30 | 4262 | 2956 | 4566 |
| N40 | 3184 | 2143 | 3416 |
| N50 | 2392 | 1562 | 2573 |
| N60 | 1765 | 1111 | 1897 |
| N70 | 1244 | 742 | 1325 |
| N80 | 799 | 446 | 827 |
| N90 | 453 | 269 | 452 |

##### Fragment size estimation

The abyss k120 contig assembly was used for mapping pair-end reads against to estimate fragment size.

The term "insert size" and "fragment size" seems to be used interchangeably in most literature. Insert size should represent the length of the distance between reads. Fragment size should represent the length of the entire fragment (insert size + length of reads on both ends). With this analysis, we estimated the fragment size.

The Short-SGL/Long-SGL libraries' paired-reads are in forward/reverse orientation while the MPL libraries pair-reads are in reverse/forward orientation. Scaffolding was done with SSPACE after estimating fragment size.

The bowtie2 command used to map reads as single end was:

```
bowtie2 --very-sensitive -p 32 --no-hd --no-sq --no-unal -x db -U lib.gz > lib.sam
#parallel echo commands
parallel echo "bowtie2 --very-sensitive -p 32 --no-hd --no-sq --no-unal -x prelim -U {} \> {/.}.sam" :::: fs
```

A python script `process.py` was used to loop through the resulting .SAM files to collect insert size data.

The following figure shows histogram of insert sizes for each library. The MPLs are separated into two types. The "with adapters" are paired reads where at least one of the pair had an junction adapter sequence (category A + B + C in nextclip). "No adapters" are paired reads where no junction adapters were found for the paired reads (category D in nextclip). Reads with no junction adapters are recommended to be discarded by Nextclip because they potentially could be a random sub-fragment from the circularized fragment.

In [63]:

```
def fragmentSize(path):
    rf = []
    fr = []
    ff = []
    rr = []
    inFile = open(path)
    count = 0
    for line in inFile:
        meta = line.strip().split()
        aOrient = meta[1]
        bOrient = meta[6]
        aStart = int(meta[2])
        aLen = int(meta[3])
        bStart = int(meta[7])
        bLen = int(meta[8])

        if aLen >= 50 and bLen >= 50: #only look at reads that have >= 50bp mapping
            if aOrient == '+':
                if bOrient == '-':
                    #if read A is + and read B is -, then coordinate of read B needs to be < coordinate of read A
                    if bStart < aStart: 
                        rf.append(aStart + aLen - bStart)
                    else:
                        fr.append(bStart + bLen - aStart)
                else:
                    if bStart < aStart:
                        ff.append(aStart + aLen + bStart)
                    else:
                        ff.append(bStart + bLen + aStart)
                    
            else:
                if bOrient == '+':
                    #if read A is - and read B is +, then coordinate of read A needs to be > coordinate of read B
                    if aStart > bStart: 
                        rf.append(bStart + bLen - aStart)
                    else:
                        fr.append(aStart + aLen - bStart)
                else:
                    if bStart < aStart:
                        rr.append(aStart + aLen + bStart)
                    else:
                        rr.append(bStart + bLen + aStart)
            
        count += 1
        if count == 500000:
            break
    inFile.close()
                        
    return (fr, rf, ff, rr)
```

In [64]:

```
fs = [x for x in os.listdir(prefix + 'fragsize') if x.split('.')[-1] == 'data']
dists = {}

for f in fs:
    name = f.split('.')[0]
    orient = 0
    if len(name.split('_')) == 2:
        orient = 1
        if name.split('_')[1] == 'D':
            name = name.split('_')[0] + ', no adpt.'
        else:
            name = name.split('_')[0] + ', with adpt.'
    
    dists[name] = pd.Series(fragmentSize(prefix + 'fragsize/' + f)[orient])
```

In [65]:

```
ks = dists.keys()
ks.sort()
for k in ks:
    dists[k]
    
fig, axes = plt.subplots(ncols = 2, nrows = 5, figsize=[15,15])

for i, d in enumerate(ks):
    col = i % 2
    row = i / 2

    pd.Series(dists[d]).hist(bins=150, ax = axes[row, col])
    axes[row,col].set_title(ks[i])
    if d == 'shortsgl' or d == 'longsgl':
        axes[row,col].set_xlim(0,1000)
    else:
        axes[row,col].set_xlim(0,20000)
    
fig.tight_layout()
```

The longSGL library seems to have a spread of insert sizes. We will not use this library in the scaffolding process. But we will use it for contigging. We would expect the MPL with no adapaters to contain more erroneous fragment sizes, but that doesn't seem like the case as the no adapters and with adapters fragment sizes are very similar. There is slightly more small fragment sizes in the no adapters paired reads. For scaffolding, we will include the reads with no adapters, against the recommendations of Nextclip.

Manually bound the peaks to get an estimate of mean and standard deviation fragment size.

In [66]:

```
shortsgl = dists['shortsgl']
kb6mpl = dists['6kbmpl, with adpt.']
kb8mpl = dists['8kbmpl, with adpt.']
kb9mpl = dists['9kbmpl, with adpt.']
kb20mpl = dists['20kbmpl, with adpt.']

fig, axes = plt.subplots(ncols = 2, nrows = 3, figsize=[15,10])
shortsgl[shortsgl < 700][shortsgl > 150].hist(bins=50, ax = axes[0,0]).set_title('shortsgl')
kb6mpl[kb6mpl < 7000][kb6mpl > 4300].hist(bins=100, ax = axes[0,1]).set_title('6kbmpl')
kb8mpl[kb8mpl < 9000][kb8mpl > 6000].hist(bins=100, ax = axes[1,0]).set_title('8kbmpl')
kb9mpl[kb9mpl < 11500][kb9mpl > 7500].hist(bins=100, ax = axes[1,1]).set_title('9kbmpl')
kb20mpl[kb20mpl < 16500][kb20mpl > 11500].hist(bins=100, ax = axes[2,0]).set_title('20kbmpl')
```

Out[66]:

```
<matplotlib.text.Text at 0x37d20ed0>
```

In [67]:

```
fragTable = ListTable()
fragTable.append(['Library','Mean fragment size','Standard Dev. fragment size'])
fragTable.append(['Short-SGL',\
                  str(shortsgl[shortsgl < 700][shortsgl > 150].mean()),\
                  str(shortsgl[shortsgl < 700][shortsgl > 150].std())])
fragTable.append(['6kb-MPL',\
                  str(kb6mpl[kb6mpl < 7000][kb6mpl > 4300].mean()),\
                  str(kb6mpl[kb6mpl < 7000][kb6mpl > 4300].std())])
fragTable.append(['8kb-MPL',\
                  str(kb8mpl[kb8mpl < 9000][kb8mpl > 6000].mean()),\
                  str(kb8mpl[kb8mpl < 9000][kb8mpl > 6000].std())])
fragTable.append(['9kb-MPL',\
                  str(kb9mpl[kb9mpl < 11500][kb9mpl > 7500].mean()),\
                  str(kb9mpl[kb9mpl < 11500][kb9mpl > 7500].std())])
fragTable.append(['20kb-MPL',\
                  str(kb20mpl[kb20mpl < 16500][kb20mpl > 11500].mean()),\
                  str(kb20mpl[kb20mpl < 16500][kb20mpl > 11500].std())])
fragTable
```

Out[67]:

|  |  |  |
| --- | --- | --- |
| Library | Mean fragment size | Standard Dev. fragment size |
| Short-SGL | 431.863446003 | 98.0617683685 |
| 6kb-MPL | 5544.37341853 | 546.646019768 |
| 8kb-MPL | 7369.21186581 | 555.852634853 |
| 9kb-MPL | 9395.47109784 | 748.352502151 |
| 20kb-MPL | 13905.4697883 | 796.858368552 |

### Contig coverage

The K-mer spectra of all reads shows a strong peak at ~57 multiplicity and a large shoulder extending to double that coverage. This indicates that a portion of the k-mers that will be used in the contig assembly is covered at twice the coverage of another portion.

By mapping all the reads back to the assembled/merged contigs, we see a distinct bimodal distribution where a portion of the contigs (~100 coverage) are covered at twice the coverage of another (~50 coverage).

In [2]:

```
from Bio import SeqIO
from collections import defaultdict
import math
def nX(lengths):
    bpCount = 0
    totalCount = sum(lengths)
    nx = {}

    for l in lengths:
        bpCount += l
        xPercent = math.floor(float(bpCount) / totalCount * 100)
        
        if not nx.has_key(xPercent):
            nx[xPercent] = l
            
    nx = nx.items()
    nx.sort(key = lambda x : x[0])
    return nx
```

In [24]:

```
inFile = open('/home/share/projects/phaw_genome/draft_3.0/assembly/segmentDuplication/k120_k70.gam.format.data')

df_data = {'cov':[],'aln':[],'ident':[],'length':[]}
df_index = []

for line in inFile:
    meta = line.strip().split()
    cov = float(meta[2])
    #was calculated as the number of matches / alignment length (bigger of the query/hit, includes gaps)
    ident = float(meta[-2])
    aln = min(1.0,float(meta[3]) / int(meta[1]))
    df_index.append(meta[0])
    df_data['cov'].append(cov)
    df_data['aln'].append(aln)
    df_data['ident'].append(ident)
    df_data['length'].append(int(meta[1]))
df_data = pd.DataFrame(df_data)
df_data.index = df_index
```

In [6]:

```
leg = []
for i in [0,500,1000,5000]:
    leg.append('length > ' + str(i))
    df_data[df_data['cov'] < 200][df_data['length'] > i]['cov'].hist(bins=100, figsize=[10,6])
    
plt.legend(leg,prop={'size':13})
```

Out[6]:

```
<matplotlib.legend.Legend at 0x1f226510>
```

Here is the k-mer spectra for all data:

In [9]:

```
pd.Series([x[1] for x in allData], index = [x[0] for x in allData])[:200].plot(kind='area', alpha=0.3,\
                                                                                     figsize=[10,6], ylim=[0,0.35e8])
```

Out[9]:

```
<matplotlib.axes.AxesSubplot at 0x125a3150>
```

In [297]:

```
histoData = [(int(line.strip().split()[0]), int(line.strip().split()[1])) for line in open('/home/share/projects/phaw_genome/draft_2.3/spectra/all.30.histo')]
s = pd.Series([x[1] for x in histoData], index = [x[0] for x in histoData])
k = s * s.index
```

In [299]:

```
print 'All K-mers:', commas(int(sum(k)))
print 'All unique K-mers:', commas(int(sum(s)))
print
print 'K-mers below 15 multiplicity (sequencing errors):', commas(int(sum(k[:15])))
print 'Unique K-mers below 15 multiplicity (sequencing errors):', commas(int(sum(s[:15])))
print
print 'K-mers above 180 multiplicity (repetitive regions):', commas(int(sum(k[180:])))
print 'Unique k-mers above 180 multiplicity (repetitive regions):', commas(int(sum(s[180:])))
print
print 'K-mers between 15-180 multiplicity:', commas(int(sum(k[15:180])))
print 'Unique K-mers between 15-180 multiplicity:', commas(int(sum(s[15:180])))
print
print 'Percentage of all k-mers between 15-180 multiplicity:', sum(k[15:180]) / float(sum(k)) * 100
print 'Average k-mer coverage between 15-180 multiplicity:',sum(k[15:180]) / float(sum(s[15:180]))
```

```
All K-mers: 270,933,539,388
All unique K-mers: 5,230,408,736

K-mers below 15 multiplicity (sequencing errors): 3,886,617,726
Unique K-mers below 15 multiplicity (sequencing errors): 2,974,163,059

K-mers above 180 multiplicity (repetitive regions): 92,127,457,496
Unique k-mers above 180 multiplicity (repetitive regions): 123,244,950

K-mers between 15-180 multiplicity: 174,919,464,166
Unique K-mers between 15-180 multiplicity: 2,133,000,727

Percentage of all k-mers between 15-180 multiplicity: 64.5617609991
Average k-mer coverage between 15-180 multiplicity: 82.0062843635
```

This bimodal distribution could possibly indicate high heterozygosity (contigs at ~50 coverage are heterozygous alleles) or segmental duplication (contigs at ~100 coverage are recently duplicated loci in the genome).

LAST was used to align the all the contigs against each other. An alignment percentage and identity was calculated for each contig by:

- alignment % = longest alignment length out of all possible hit contigs / length of query contig
- identity = number of matches / longest alignment length out of all possible hit contigs

The following graph shows the proportion of contigs at various aignment %:

In [162]:

```
df_data[df_data['cov'] < 200]['cov'].hist(bins=150, figsize=[10,6])
df_data[df_data['cov'] < 200][df_data['aln'] >= 0.7]['cov'].hist(bins=150, figsize=[10,6])
df_data[df_data['cov'] < 200][df_data['aln'] >= 0.8]['cov'].hist(bins=150, figsize=[10,6])
df_data[df_data['cov'] < 200][df_data['aln'] >= 0.9]['cov'].hist(bins=150, figsize=[10,6])
df_data[df_data['cov'] < 200][df_data['aln'] >= 0.95]['cov'].hist(bins=150, figsize=[10,6])
df_data[df_data['cov'] < 200][df_data['aln'] == 1]['cov'].hist(bins=150, figsize=[10,6])
plt.legend(['all contigs',\
            'at least 70% alignment to another contig',\
            'at least 80% alignment to another contig',\
            'at least 90% alignment to another contig',\
            'at least 95% alignment to another contig',\
            '100% alignment to another contig'],prop={'size':14})
```

Out[162]:

```
<matplotlib.legend.Legend at 0x6a23c850>
```

In [151]:

```
df_data['aln'][df_data['aln'] > 0.5].hist(bins=50,figsize=[10,5])
```

Out[151]:

```
<matplotlib.axes.AxesSubplot at 0x7b8a8e90>
```

There is a peak at >= ~95% alignment %.

The following is the distribution of identities for contigs >= 95% alignment. There is a sharp drop off at ~97% identity. Any contigs that are >= 97% indentity and >= 95% alignment were probably collapsed into one contig during the assembly process.

In [144]:

```
df_data[df_data['aln'] >= 0.95]['ident'].hist(bins= 100,figsize=[10,6])
```

Out[144]:

```
<matplotlib.axes.AxesSubplot at 0x66781990>
```

Here are the proportions of contigs that have >= 95% alignment at various identities.

In [155]:

```
df_data[df_data['cov'] < 200]['cov'].hist(bins=150, figsize=[10,6])
df_data[df_data['ident'] >= 0.7][df_data['aln'] >= 0.95][df_data['cov'] < 200]['cov'].hist(bins=150, figsize=[10,6])
df_data[df_data['ident'] >= 0.8][df_data['aln'] >= 0.95][df_data['cov'] < 200]['cov'].hist(bins=150, figsize=[10,6])
df_data[df_data['ident'] >= 0.9][df_data['aln'] >= 0.95][df_data['cov'] < 200]['cov'].hist(bins=150, figsize=[10,6])
df_data[df_data['ident'] >= 0.95][df_data['aln'] >= 0.95][df_data['cov'] < 200]['cov'].hist(bins=150, figsize=[10,6])
plt.legend(['all contigs',\
            'at least 70% identity',\
            'at least 80% identity',\
            'at least 90% identity',\
            'at least 95% identity',],prop={'size':14})
```

Out[155]:

```
<matplotlib.legend.Legend at 0x88abfa90>
```

In [303]:

```
print 'Number of contigs total:', commas(len(df_data['length']))
print 'Sum bases of contigs:', commas(int(sum(df_data['length'])))
print
print 'Number of contigs between 10-110 (lower covered contigs):', \
commas(len(df_data[df_data['cov']>=10][df_data['cov']<=110]['length']))
print 'Sum bases of contigs between 10-110 (lower covered contigs):', \
commas(int(sum(df_data[df_data['cov']>=10][df_data['cov']<=110]['length'])))
print
print 'Number of contigs >= 95% alignment:', commas(len(df_data[df_data['aln'] >= 0.95]['length']))
print 'Sum bases of contigs >= 95% alignment:', commas(int(sum(df_data[df_data['aln'] >= 0.95]['length'])))
print 'Percentage of lower covered contigs:', \
100 * float(sum(df_data[df_data['aln'] >= 0.95]['length'])) / sum(df_data[df_data['cov']>=10][df_data['cov']<=110]['length'])
print
print 'Number of contigs >= 95% alignment and >= 97% identity:', \
commas(len(df_data[df_data['aln'] >= 0.95][df_data['ident'] >= 0.97]))
print 'Sum bases of contigs >= 95% alignment and >= 97% identity:', \
commas(int(sum(df_data[df_data['aln'] >= 0.95][df_data['ident'] >= 0.97]['length'])))
print 'Percentage of lower covered contigs:', \
100 * float(sum(df_data[df_data['aln'] >= 0.95]\
                [df_data['ident'] >= 0.97]['length'])) / sum(df_data[df_data['cov']>=10][df_data['cov']<=110]['length'])
```

```
Number of contigs total: 2,285,183
Sum bases of contigs: 2,977,829,429

Number of contigs between 10-110 (lower covered contigs): 1,882,641
Sum bases of contigs between 10-110 (lower covered contigs): 2,107,173,518

Number of contigs >= 95% alignment: 1,158,257
Sum bases of contigs >= 95% alignment: 626,331,348
Percentage of lower covered contigs: 29.7237670581

Number of contigs >= 95% alignment and >= 97% identity: 181,830
Sum bases of contigs >= 95% alignment and >= 97% identity: 84,518,128
Percentage of lower covered contigs: 4.01097144008
```

In [97]:

```
print 'Sum bases of contigs between 10-110 (lower covered contigs):', commas(int(sum(df_data[df_data['cov']<=110]['length'])))
```

```
Sum bases of contigs between 10-110 (lower covered contigs): 2,110,420,222
```

### Contig filtering

Used CD-HIT to collapse contigs that are at least 96% identity of another.

In [4]:

```
cdhitFile = open('/home/share/projects/phaw_genome/draft_3.0/assembly/contig/k120_k70.gam.format.cdhit.fa.clstr')

blocks = cdhitFile.read().strip().split('>Cluster')[1:]
```

In [5]:

```
ctgLengths = {}
for line in open('/home/share/projects/phaw_genome/draft_3.0/assembly/segmentDuplication/k120_k70.gam.format.lengths'):
    meta = line.strip().split()
    ctgLengths[meta[0]] = int(meta[1])
```

In [6]:

```
cdhit_clusters = {}
cdhit_members = []
for block in blocks:
    lines = block.strip().split('\n')
    primary = 0
    members = []
    for line in lines[1:]:
        cid = line.strip().split('>')[-1].split('...')[0]
        status = line.strip().split('...')[-1].strip()

        if status == '*':
            primary = cid
        else:
            members.append(cid)
            
    cdhit_clusters[primary] = members
    cdhit_members.extend(members)
```

In [15]:

```
print 'Number of contigs total:', commas(len(ctgLengths))
print 'Sum bases of contigs:', commas(sum(ctgLengths.values()))
print
print 'Number of contigs after CD-HIT:', commas(len(cdhit_clusters))
print 'Sum bases of contig after CD-HIT:', commas(sum([ctgLengths[k] for k in cdhit_clusters.keys()]))
print
print 'Number of contigs collapsed:', commas(sum([len(v) for v in cdhit_clusters.values()]))
print 'Sum bases of contigs collapsed:', commas(sum([ctgLengths[x] for x in cdhit_members]))
```

```
Number of contigs total: 2,294,695
Sum bases of contigs: 2,981,518,945

Number of contigs after CD-HIT: 1,710,303
Sum bases of contig after CD-HIT: 2,740,538,421

Number of contigs collapsed: 584,392
Sum bases of contigs collapsed: 240,980,524
```

In [16]:

```
cdhit_member_lengths = pd.Series([ctgLengths[x] for x in cdhit_members])
```

In [17]:

```
print 'Mean length of cdhit collapsed contigs:', cdhit_member_lengths.mean()
print 'Median length of cdhit collapsed contigs:', cdhit_member_lengths.median()
```

```
Mean length of cdhit collapsed contigs: 412.361093239
Median length of cdhit collapsed contigs: 338.0
```

In [20]:

```
df_cdhit = df_data.loc[cdhit_members]
df_data[df_data['cov'] < 200]['cov'].hist(bins=150, figsize=[10,6])
df_cdhit[df_cdhit['cov'] < 200]['cov'].hist(bins=150, figsize=[10,6])
plt.legend(['All contigs','Collapsed contigs'],prop={'size':13})
```

Out[20]:

```
<matplotlib.legend.Legend at 0x2b096350>
```

Primary sequences that were kept for each CD-HIT cluster

In [43]:

```
df_primary = df_data.loc[[k for k,v in cdhit_clusters.items() if len(v) > 0]]
df_data[df_data['cov'] < 200]['cov'].hist(bins=150, figsize=[10,6])
df_primary[df_primary['cov'] < 200]['cov'].hist(bins=150, figsize=[10,6])
```

Out[43]:

```
<matplotlib.axes.AxesSubplot at 0x3b918a50>
```

In [125]:

```
df_cdhit = df_data.loc[cdhit_members]
df_data[df_data['cov'] < 200]['cov'].hist(bins=150, figsize=[10,6])
df_cdhit[df_cdhit['cov'] < 200]['cov'].hist(bins=150, figsize=[10,6])
df_primary[df_primary['cov'] < 200]['cov'].hist(bins=150, figsize=[10,6])
plt.legend(['All contigs','Collapsed contigs','primary contigs'],prop={'size':13})
```

Out[125]:

```
<matplotlib.legend.Legend at 0x66aebe90>
```

In [349]:

```
df_filtered = df_data.loc[list(set(df_data.index) - set(cdhit_members))]
```

In [352]:

```
leg = []
for i in [0,500,1000,5000]:
    leg.append('length > ' + str(i))
    df_filtered[df_filtered['cov'] < 200][df_filtered['length'] > i]['cov'].hist(bins=150, figsize=[10,6])
    
plt.legend(leg,prop={'size':13})
```

Out[352]:

```
<matplotlib.legend.Legend at 0x1391944d0>
```

### Scaffolding

The scaffolding process starts by first remapping reads back the contig assembly as single ended reads using bowtie2:

```
bowtie2 --very-sensitive -p 32 --no-hd --no-sq --no-unal -x database -U lib.fastq.gz > lib.sam
```

Links are extracted from the resilting .sam files:

```
python extractLinks.py a.sam b.sam output
```

This script outputs two files: `output.diffRef.data`, `output.sameRef.data`

Fragment size can be estimated from the `output.sameRef.data` file. The `output.diffRef.data` file can be used for scaffolding.

A masking process is then performed to filter out reads that mapped to regions of high coverage. Even though only uniquely mapped reads are used, bowtie2 does allow for mismatches, resulting in possible pseudo-unique mappings to alternate contigs assembled at regions of high coverage.

A better method is to probably use Myer's A-statistic (log odds ratio of contig being unique vs collapsed repetitive region).

This masking process starts by first using jellyfish to get 30mers from the contig assembly. The the following scripts are used to mask the `output.diffRef.data` file:

```
jellyfish count -C -m 30 -t 30 -s 1000000000 -o out.kmer.30.jf contigs.fa
jellyfish dump -L 2 -o out.kmer.30.fa out.kmer.30.jf
python getKmerCoverage.py out.kmer.30.fa contigs.fa contigs.cov
python maskData contigs.cov output.diffRef.data > output.diffRef.masked.data
```

The links are then converted to a tab delimited format which SSPACE can accept. SSPACE was then used to the scaffold the contigs using the following config:

```
shortsgl TAB shortsgl.diffRef.masked.sspace.data 431 0.22 FR
6kbmpl TAB 6kbmpl.contig.paired.diffRef.masked.sspace.data 5544 0.1 RF
8kbmpl TAB 8kbmpl.contig.paired.diffRef.masked.sspace.data 7369 0.08 RF
9kbmpl TAB 9kbmpl.contig.paired.diffRef.masked.sspace.data 9395 0.08 RF
20kbmpl TAB 20kbmpl.contig.paired.diffRef.masked.sspace.data 13905 0.06 RF
```

### Final assembly stats

In [53]:

```
from Bio import SeqIO
from collections import defaultdict
import math

def faStats(path):
    faFile = open(path)
    lengths = []
    nCount = []
    ungapped_lengths = []
    
    for record in SeqIO.parse(faFile,'fasta'):
        seq = str(record.seq).lower()
        ns = seq.count('n')
        nCount.append(ns)
        lengths.append(len(seq))
        ungapped_lengths.append(len(seq) - ns)
    
    faFile.close()
    
    print '---with Ns----'
    print 'Sum bases: ' + str(commas(sum(lengths)))
    print 'Max length: ' + str(commas(max(lengths)))
    print 'Min length: ' + str(commas(min(lengths)))
    print 'Number of Ns total: ' + str(commas(sum(nCount)))
    print 'Avg number of Ns per scaffold: ' + str(sum(nCount) / float(len(nCount)))
    lengths.sort(reverse = True)
    scfNx = nX(lengths)
    print
    for i in range(10):
        print 'N' + str(i * 10) + ':', commas(int(scfNx[i * 10][1]))
    print
    print '---without Ns----'
    print 'Sum bases: ' + str(commas(sum(ungapped_lengths)))
    print 'Max length: ' + str(commas(max(ungapped_lengths)))
    print 'Min length: ' + str(commas(min(ungapped_lengths)))
    ungapped_lengths.sort(reverse = True)
    scfNx = nX(ungapped_lengths)
    print
    for i in range(10):
        print 'N' + str(i * 10) + ':', commas(int(scfNx[i * 10][1]))
    print
    
def plotLength(path):
    faFile = open(path)
    lengths = []
    nCount = []
    ungapped_lengths = []
    
    for record in SeqIO.parse(faFile,'fasta'):
        seq = str(record.seq).lower()
        ns = seq.count('n')
        nCount.append(ns)
        lengths.append(len(seq))
        ungapped_lengths.append(len(seq) - ns)
    
    faFile.close()
```

In [58]:

```
print 'Number of scaffolds: 133,035'
print 'Number of unplaced contigs: 259,343'
```

```
Number of scaffolds: 133,035
Number of unplaced contigs: 259,343
```

In [56]:

```
print "scaffolds"
faStats('/home/share/projects/phaw_genome/draft_3.0/assembly/phaw_30.scf.fa')
```

```
scaffolds
---with Ns----
Sum bases: 3,635,838,320
Max length: 1,285,385
Min length: 368
Number of Ns total: 1,102,583,489
Avg number of Ns per scaffold: 8287.92038937

N0: 1,285,385
N10: 289,705
N20: 192,210
N30: 142,339
N40: 107,526
N50: 81,190
N60: 59,937
N70: 41,969
N80: 26,835
N90: 14,799

---without Ns----
Sum bases: 2,533,254,831
Max length: 1,128,096
Min length: 368

N0: 1,128,096
N10: 264,499
N20: 173,954
N30: 127,684
N40: 96,443
N50: 73,743
N60: 55,316
N70: 39,746
N80: 25,187
N90: 11,065
```

In [57]:

```
print "unplaced contigs (degenerate contigs)"
faStats('/home/share/projects/phaw_genome/draft_3.0/assembly/phaw_30.degen.fa')
```

```
unplaced contigs (degenerate contigs)
---with Ns----
Sum bases: 146,994,949
Max length: 40,222
Min length: 240
Number of Ns total: 23,431
Avg number of Ns per scaffold: 0.0903475320329

N0: 40,222
N10: 1,779
N20: 1,235
N30: 943
N40: 758
N50: 627
N60: 525
N70: 442
N80: 370
N90: 304

---without Ns----
Sum bases: 146,971,518
Max length: 40,222
Min length: 240

N0: 40,222
N10: 1,779
N20: 1,235
N30: 943
N40: 758
N50: 626
N60: 525
N70: 442
N80: 370
N90: 304
```

In [91]:

```
print '15,160 Scaffolds with gene predictions'
faStats('/home/share/projects/phaw_genome/draft_3.0/assembly/phaw_30.genic.fa')
```

```
15,160 Scaffolds with gene predictions
---with Ns----
Sum bases: 1,493,864,592
Max length: 1,285,385
Min length: 242
Number of Ns total: 323,472,058
Avg number of Ns per scaffold: 21337.2069921

N0: 1,285,385
N10: 433,836
N20: 308,824
N30: 243,485
N40: 197,185
N50: 161,819
N60: 132,210
N70: 105,291
N80: 80,030
N90: 52,952

---without Ns----
Sum bases: 1,170,392,534
Max length: 1,128,096
Min length: 242

N0: 1,128,096
N10: 379,216
N20: 267,896
N30: 205,954
N40: 165,725
N50: 133,911
N60: 108,383
N70: 85,178
N80: 63,745
N90: 41,827
```

In [153]:

```
print 'removed contigs'
faStats('/home/share/projects/phaw_genome/draft_3.0/assembly/contig/k120_k70.gam.format.cdhit.collapsed.fa')
```

```
removed contigs
---with Ns----
Sum bases: 240,980,524
Max length: 24,461
Min length: 169
Number of Ns total: 627
Avg number of Ns per scaffold: 0.00107290996454

N0: 24,461
N10: 1,038
N20: 698
N30: 544
N40: 459
N50: 402
N60: 357
N70: 324
N80: 293
N90: 265

---without Ns----
Sum bases: 240,979,897
Max length: 24,461
Min length: 169

N0: 24,461
N10: 1,038
N20: 698
N30: 544
N40: 459
N50: 402
N60: 357
N70: 324
N80: 293
N90: 265
```

### CEGMA

There are 1,488 proteins representing 248 genes in the CEGMA database. I blasted the transcriptome and genome against the 1,488 proteins. For each of the found 248 genes, I plotted the coverage across the CEGMA protein and e-value.

The transcriptome shows good coverage for 247/248 CEGMA genes. The genome shows moderate coverage for 244/248 of the CEGMA genes reflecting the high level of fragmentation in the genome.

In [203]:

```
def tileCoords(coords):
	formatCoords = []
	for coord in coords:
		coord = [int(coord[0]),int(coord[1])]

		formatCoords.append(('s',min(coord)))
		formatCoords.append(('e',max(coord)))

	formatCoords.sort(key = lambda x : x[0], reverse = True)
	formatCoords.sort(key = lambda x : x[1])

	count = 0
	posA = 0
	out = []
	for pos in formatCoords:
		if count == 0:
			posA = pos[1]
		if pos[0] == 's':
			count += 1
		if pos[0] == 'e':
			count -=1

		if count == 0:
			out.append((posA, pos[1]))

	return out
```

In [219]:

```
def cegmaStat(path):
    cegmaFile = open(path)
    hits = defaultdict(list)
    
    for line in cegmaFile:
        meta = line.strip().split()
        k = (meta[0],meta[1])
        hits[k].append([int(meta[6]),int(meta[7]),float(meta[-1]),int(meta[2])])

    kogs = defaultdict(list)
    for hit, data in hits.items():
        tCoords = tileCoords(data)
        evalue = min([x[2] for x in data])
        klength = data[0][3]
        hitLength = sum([x[1] - x[0] + 1 for x in tCoords])
        
        kogs[hit[0]].append((hitLength / float(klength), evalue, hit[1]))
    
    bestKog = defaultdict(lambda : (0,0,0))
    for kog, data in kogs.items():
        data.sort(key = lambda x : x[0], reverse = True)
        kid = kog.split('_')[-1]
        if bestKog[kid][0] < data[0][0]:
            bestKog[kid] = (data[0][0], data[0][1], data[0][2])
        
    return bestKog
```

In [221]:

```
transCegma = cegmaStat('/home/share/projects/phaw_genome/draft_3.0/transcriptome/cegma/phaw_AP.cegma')
```

In [224]:

```
df_transCegma = []
for kog, data in transCegma.items():
    df_transCegma.append([kog, data[0],data[1],data[2]])
df_transCegma = pd.DataFrame(df_transCegma)
```

In [226]:

```
df_transCegma.columns = ['KogID','coverage','evalue','tid']
```

In [245]:

```
df_transCegma.plot(kind='scatter',x='coverage',y='evalue',logy=True,fontsize=50,figsize=[10,6])
```

Out[245]:

```
<matplotlib.axes.AxesSubplot at 0x6f40b3d0>
```

In [237]:

```
genomeCegma = cegmaStat('/home/share/projects/phaw_genome/draft_3.0/assembly/cegma/phaw_30.variantctg.cegma')
```

In [238]:

```
df_genomeCegma = []
for kog, data in genomeCegma.items():
    df_genomeCegma.append([kog, data[0],data[1],data[2]])
df_genomeCegma = pd.DataFrame(df_genomeCegma)
df_genomeCegma.columns = ['KogID','coverage','evalue','tid']
```

In [239]:

```
df_genomeCegma.plot(kind='scatter',x='coverage',y='evalue',logy=True,figsize=[10,6])
```

Out[239]:

```
<matplotlib.axes.AxesSubplot at 0x6cb5c8d0>
```
