# Supplementary material for: The genome of the crustacean Parhyale hawaiensis, a model for animal development, regeneration, immunity and lignocellulose digestion
Source: eLife. 2016 Nov 16;5:e20062. doi: 10.7554/eLife.20062 (PMC5111886; doi:10.7554/eLife.20062)
Supplement: Source code 2. — Includes repeat analysis of the Parhyale genome using Repeat Modeller and Repeat Masker. DOI: http://dx.doi.org/10.7554/eLife.20062.047 [file elife-20062-code2.htm]

Notebook


In [8]:

```
import gzip, os, sys
#import custom functions for displaying tables, bash commands
sys.path.append(os.path.abspath("/home/damian/"))
from dk_ipython import *
from IPython.display import HTML
import pandas as pd
import seaborn as sns
import matplotlib.pyplot as plt
import scipy.stats as ss
import scipy as sp

%matplotlib inline
HTML(addToggle())
```

Out[8]:

The raw code for this IPython notebook is by default hidden for easier reading.
To toggle on/off the raw code, click here.

## Repeats

RepeatModeler and RepeatMasker were used on the genome.

In [9]:

```
prefix = '/home/share/projects/phaw_genome/draft_2.3_final/repeatmasker/data/'
```

In [10]:

```
indexFile = open(prefix + 'db.index')
index = {}

for line in indexFile:
    meta = line.strip().split('#')
    index[meta[0]] = meta[1].split()[0]
    
#ran Dfam against unknown repeats found by repeatmodeler. 11 unknowns were classified:
additional = '''rnd-4_family-4277#SINE/AluSg4
rnd-5_family-1876#RC/Helitron2Na_Mam
rnd-2_family-234#RC/Helitron3Na_Mam
rnd-2_family-128#RC/Helitron3Na_Mam
rnd-5_family-1876#RC/Helitron3Na_Mam
rnd-4_family-493#RC/Helitron3Na_Mam
rnd-2_family-332#RC/Helitron3Na_Mam
rnd-6_family-1392#RC/Helitron3Na_Mam
rnd-4_family-337#DNA/MER44A
rnd-4_family-1409#DNA/Tigger7
rnd-4_family-54#tRNA/tRNA-Tyr-TAC'''.split('\n')
for x in additional:
    meta = x.split('#')
    index[meta[0]] = meta[1]
```

In [11]:

```
from collections import defaultdict
repeatFile = open(prefix + 'repeat.sorted')
repeats = defaultdict(int)

unknowns = []
for line in repeatFile:
    meta = line.strip().split()
    if meta[0][:3] == 'rnd':
        repeats[index[meta[0]]] += int(meta[1])
        if index[meta[0]] == "Unknown":
            unknowns.append(meta[0])
    else:
        repeats[meta[0]] = int(meta[1])
        
repeats = repeats.items()
repeats.sort(key = lambda x : x[1], reverse = True)
```

In [12]:

```
repeatCounts = open(prefix + 'repeat.element.count')

repeatTags = defaultdict(list)

for line in repeatCounts:
    meta = line.strip().split('\t')
    rid = '_'.join(meta[0].split('_')[:2])
    start,end = meta[0].split('_')[-2:]
    l = int(end) - int(start) + 1
    repeatType = index[rid]
    
    if int(meta[1]) > 0:
        repeatTags[repeatType].append((l,int(meta[1]), start, end, meta[0].split('_')[-3], rid))
```

In [13]:

```
print 'bases of repeats:', sum([x[1] for x in repeats])
print '% of genome:', sum([x[1] for x in repeats]) / 4237229237.0 * 100
print '% of genome:', sum([x[1] for x in repeats]) / 2939407201.0 * 100
```

```
bases of repeats: 1583691670
% of genome: 37.375642936
% of genome: 53.8779271365
```

In [65]:

```
repeatTable = ListTable()
repeatTable.append(['repeat type','bases','% genome','# tags','number of bases'])

for item in repeats:
    repeatTable.append([item[0],\
                        commas(item[1]),\
                        item[1] / 4237229237.0 * 100,\
                        commas(sum([x[1] for x in repeatTags[item[0]]])),\
                        commas(sum([x[0] for x in repeatTags[item[0]]]))])
repeatTable
```

Out[65]:

|  |  |  |  |  |
| --- | --- | --- | --- | --- |
| repeat type | bases | % genome | # tags | number of bases |
| Unknown | 935,233,022 | 22.0718061188 | 32,889,026 | 217,412,412 |
| RC/Helitron3Na\_Mam | 101,451,815 | 2.39429611488 | 1,408,381 | 14,843,982 |
| LINE/CR1 | 83,856,505 | 1.9790410268 | 1,163,649 | 26,574,062 |
| DNA/TcMar-Tc1 | 68,800,466 | 1.62371356733 | 1,097,485 | 19,260,914 |
| LINE/CR1-Zenon | 66,234,301 | 1.56315123151 | 913,541 | 22,571,609 |
| LINE/Penelope | 37,792,370 | 0.891912329642 | 383,764 | 8,122,950 |
| LINE/Jockey | 26,618,441 | 0.628203939677 | 1,003,441 | 10,181,920 |
| LINE/L2 | 26,279,152 | 0.620196607975 | 296,593 | 7,231,295 |
| Simple\_repeat | 20,105,314 | 0.474492005871 | 138,709 | 2,450,699 |
| DNA | 19,850,762 | 0.468484495166 | 563,621 | 4,504,002 |
| DNA/hAT | 19,004,903 | 0.448521945286 | 122,935 | 3,020,093 |
| DNA/TcMar-Mariner | 17,929,117 | 0.423133042778 | 332,566 | 4,016,488 |
| LINE/I | 17,212,473 | 0.406220009286 | 1,045,699 | 10,060,579 |
| LINE/Proto2 | 16,193,628 | 0.382174933058 | 178,252 | 4,102,078 |
| short | 14,614,446 | 0.344905719813 | 0 | 0 |
| LTR/Gypsy | 12,002,252 | 0.283257084493 | 270,335 | 5,307,125 |
| LINE/Dong-R4 | 8,950,083 | 0.21122489484 | 67,028 | 2,889,126 |
| DNA/hAT-Tip100 | 7,315,075 | 0.172638169682 | 45,153 | 1,657,740 |
| DNA/Maverick | 6,431,581 | 0.151787421455 | 33,601 | 2,554,784 |
| LINE/RTE-BovB | 6,330,019 | 0.149390524938 | 55,334 | 1,661,551 |
| DNA/hAT-Charlie | 5,638,368 | 0.133067334445 | 244,675 | 1,165,914 |
| LTR/Pao | 5,340,240 | 0.126031415845 | 24,397 | 1,496,645 |
| DNA/hAT-Tag1 | 4,452,813 | 0.105087847528 | 69,971 | 1,287,416 |
| Satellite | 4,372,776 | 0.103198948072 | 234,115 | 2,164,104 |
| DNA/MER44A | 4,084,747 | 0.0964013691856 | 37,865 | 707,090 |
| DNA/MULE-MuDR | 4,001,542 | 0.0944377038905 | 72,515 | 742,631 |
| LINE/I-Nimb | 3,722,118 | 0.0878432058265 | 114,096 | 2,022,292 |
| DNA/CMC-Chapaev-3 | 3,471,760 | 0.0819346748976 | 80,844 | 556,876 |
| DNA/TcMar-Tigger | 3,211,307 | 0.0757878986569 | 46,944 | 588,020 |
| DNA/hAT-Ac | 3,078,352 | 0.0726501170416 | 128,451 | 1,174,197 |
| LINE/RTE-X | 2,744,437 | 0.064769613502 | 22,085 | 1,137,235 |
| DNA/Ginger | 2,580,371 | 0.0608976020808 | 44,607 | 611,435 |
| DNA/CMC-EnSpm | 2,203,524 | 0.0520038892576 | 14,062 | 535,386 |
| LTR/Copia | 1,734,822 | 0.0409423683017 | 11,660 | 626,602 |
| DNA/P | 1,562,840 | 0.0368835366837 | 33,717 | 410,739 |
| LINE | 1,513,129 | 0.0357103407762 | 14,007 | 506,116 |
| LTR/DIRS | 1,446,892 | 0.0341471258474 | 445,125 | 1,017,888 |
| DNA/PIF-Harbinger | 1,298,397 | 0.0306425951342 | 28,009 | 390,000 |
| DNA/hAT-Blackjack | 1,148,525 | 0.0271055667692 | 35,677 | 261,406 |
| DNA/hAT-hAT5 | 1,141,398 | 0.0269373672312 | 76,288 | 598,844 |
| DNA/hAT-Tol2 | 1,107,684 | 0.0261417057715 | 4,743 | 163,772 |
| DNA/Tigger7 | 1,074,729 | 0.0253639569607 | 11,617 | 519,733 |
| DNA/hAT-hATm | 1,068,486 | 0.0252166201127 | 14,477 | 162,618 |
| DNA/Merlin | 936,885 | 0.0221107933415 | 12,915 | 103,981 |
| tRNA/tRNA-Tyr-TAC | 798,129 | 0.0188361062232 | 11,056 | 70,914 |
| A-rich | 784,194 | 0.0185072356518 | 0 | 0 |
| DNA/CMC-Chapaev | 630,495 | 0.0148798888315 | 10,328 | 48,023 |
| DNA/Academ | 574,063 | 0.0135480751192 | 1,046 | 81,771 |
| LINE/L1-Tx1 | 524,451 | 0.0123772156441 | 547 | 44,765 |
| DNA/Sola | 494,505 | 0.0116704802205 | 16,971 | 181,976 |
| DNA/MuLE-MuDR | 483,236 | 0.0114045281237 | 4,024 | 145,202 |
| DNA/hAT-hATx | 453,462 | 0.0107018519565 | 4,520 | 40,765 |
| DNA/TcMar-Stowaway | 453,269 | 0.0106972970932 | 22,499 | 78,386 |
| DNA/TcMar-m44 | 419,014 | 0.00988886785594 | 7,743 | 81,518 |
| DNA/hAT-hobo | 411,985 | 0.0097229811501 | 3,421 | 125,269 |
| DNA/PiggyBac | 411,256 | 0.00970577651095 | 3,826 | 120,442 |
| SINE/AluSg4 | 362,484 | 0.00855474131149 | 33,966 | 72,604 |
| LTR | 330,040 | 0.00778905226836 | 6,205 | 100,873 |
| DNA/TcMar-Fot1 | 324,492 | 0.00765811764836 | 6,739 | 103,615 |
| LINE/DRE | 319,010 | 0.00752874065001 | 1,348 | 38,563 |
| GA-rich | 225,142 | 0.00531342505697 | 0 | 0 |
| LINE/CRE-II | 209,268 | 0.00493879344956 | 1,539 | 46,786 |
| LTR/ERVL | 158,220 | 0.00373404390346 | 492 | 13,662 |
| DNA/TcMar-ISRm11 | 118,126 | 0.00278781235078 | 499 | 29,284 |
| G-rich | 64,962 | 0.00153312451053 | 0 | 0 |
