# Supplementary material for: The genome of the crustacean Parhyale hawaiensis, a model for animal development, regeneration, immunity and lignocellulose digestion
Source: eLife. 2016 Nov 16;5:e20062. doi: 10.7554/eLife.20062 (PMC5111886; doi:10.7554/eLife.20062)
Supplement: Source code 3. — Parhyale transcriptome assembly, genome annotation and generation of canonical proteome dataset. DOI: http://dx.doi.org/10.7554/eLife.20062.048 [file elife-20062-code3.htm]

Notebook


In [3]:

```
import gzip, os, sys
from dk_ipython import *
from IPython.display import HTML
import pandas as pd
import seaborn as sns
import matplotlib.pyplot as plt
import scipy.stats as ss
import scipy as sp
import numpy as np
from collections import defaultdict

#import custom functions for displaying tables, bash commands
sys.path.append(os.path.abspath("/home/damian/"))
%matplotlib inline
HTML(addToggle())
```

Out[3]:

The raw code for this IPython notebook is by default hidden for easier reading.
To toggle on/off the raw code, click here.

## Parhyale hawaiensis transcriptome assembly, genome annotation and generation of canonical proteome dataset

### Transcriptome assembly

Assembly was performed with Trinity by Dr. Nikolaos Konstantinides. Pair-end sequencing data was generated from mixed pool of regenerating animals (Michalis Averof's lab) and developmental stages (Nipam Patel's lab).

Both forward (36,604,779,561) and reverse reads (35,860,233,747) combined to a total of 72,465,013,308 (72gb) of data.

Cutadapt was ran to get rid of adapters and splice-leader sequences:

```
cutadapt -b GAATTTTCACTGTTCCCTTTACCACGTTTTACTG -b TTACCAATCACCCCTTTACCAAGCGTTTACTG -b CCCTTTACCAACTCTTAACTG -b CCCTTTACCAACTTTACTG -e 0.2 (fastq file)

cutadapt -a (adapter for each case) (fastq file)
```

Trinity was ran with the following command:

```
Trinity --JM 650G --CPU 32 --seqType fq --left (fastq files) --right (fastq files) --output /home/nkonstan/TRINITY --path_reinforcement_distance 50
```

In [2]:

```
import math
def nX(lengths):
    bpCount = 0
    totalCount = sum(lengths)
    nx = {}

    for l in lengths:
        bpCount += l
        xPercent = math.floor(float(bpCount) / totalCount * 100)
        
        if not nx.has_key(xPercent):
            nx[xPercent] = l
            
    nx = nx.items()
    nx.sort(key = lambda x : x[0])
    return nx

from Bio import SeqIO
tran_lengths = []
comps = set()
faFile = open('/home/share/transcriptome/phaw/nikos.v2/Trinity_AverofPatel_Feb2015.fasta')
for record in SeqIO.parse(faFile,'fasta'):
    tran_lengths.append(len(str(record.seq)))
    comps.add(record.id.split('_')[0])

tran_nx = nX(tran_lengths)
```

In [6]:

```
print 'Transcriptome stats:'
print 'Number of transcripts:', commas(len(tran_lengths))
print 'Number of trinity components:', commas(len(comps))
print
for i in range(10):
    print 'N' + str(i * 10) + ':', commas(int(tran_nx[i * 10][1]))
```

```
Transcriptome stats:
Number of transcripts: 292,924
Number of trinity components: 276,734

N0: 1,003
N10: 1,831
N20: 3,472
N30: 5,760
N40: 1,740
N50: 1,996
N60: 2,479
N70: 1,683
N80: 4,572
N90: 8,436
```

#### Blast to Uniprot and selected proteomes/transcriptomes

Blasted the transcriptome to Uniprot and the following proteomes/transcriptomes:

```
lvan.transcriptome.fa    Litopenaeus vannamei (decapoda)
dpul.proteome.fa    Daphnia pulex (branchiopoda)
smar.proteome.fa    Strigamia maritima (myriapoda)
even.proteome.fa    Echinogammarus veneris (amphipoda)
eser.proteome.fa    Eucyclops serrulatus (copepoda)
cfin.transcriptome.fa    Calanus finmarchicus (copepoda)
cypridininae.transcriptome.fa    cypridininae (ostracoda)
stul.transcriptome.fa    Speleonectes tulumensis (remipedia)
mmar.proteome.fa    Mesobuthus martensii (chelicerata)
smim.proteome.fa    Stegodyphus mimosarum (chelicerata)
isca.proteome.fa    Ixodes scapularis (chelicerata)
bflo.proteome.fa    Branchiostoma floridae (chordata)
cele.proteome.fa    Caenorhabditis  elegans (nematoda)
dmel.proteome.fa    Drosophila melanogaster (hexapoda)
mmus.proteome.fa    Mus musculus (vertebrata)
hsap.proteome.fa    Homo sapiens (vertebrata)
dd_smed_v4.fa    Schmidtea mediterranea (lophotrochozoa)
```

In [9]:

```
data = [[x.strip().split()[1], int(x.strip().split()[0])] for x in '''   1574 bflo
   1114 cele
   1848 cfin
   1729 cypri
   1515 dmel
   2067 dpul
   1375 eser
   6788 even
    917 hsap
   1221 isca
   5110 lvan
   1414 mmar
    885 mmus
   2005 smar
   1250 smed
   1619 smim
   2341 stul'''.split('\n')]

data.sort(key = lambda x : x[1], reverse = True)
```

In [11]:

```
hitTable = ListTable()
hitTable.append(['species','number of tophits'])
for item in data:
    hitTable.append([item[0],commas(item[1])])
hitTable
```

Out[11]:

|  |  |
| --- | --- |
| species | number of tophits |
| even | 6,788 |
| lvan | 5,110 |
| stul | 2,341 |
| dpul | 2,067 |
| smar | 2,005 |
| cfin | 1,848 |
| cypri | 1,729 |
| smim | 1,619 |
| bflo | 1,574 |
| dmel | 1,515 |
| mmar | 1,414 |
| eser | 1,375 |
| smed | 1,250 |
| isca | 1,221 |
| cele | 1,114 |
| hsap | 917 |
| mmus | 885 |

### Annotation process

Three de novo transcriptome assemblies and one reference assembly are available: Averof-Patel transcriptome, Extavour transcriptome, DeepSeq transcriptome, Averof-Patel cufflinks transcriptome.

The three de novo assembled transcriptome were mapped to the genome with GMAP. These mappings along with the Cufflinks annotations were fed into PASA to consolidate the transcriptome data into a gene set (141,285).

Proteomes from selected non-Arthropods and all Arthropod uniprot entries were aligned to the genome with exonerate.

The PASA gene set, genemark gene predictions, proteome mapping, and known parhyale genes were fed into evidence modeler with the following weights to produce final gene predictions (156,800).

```
ABINITIO_PREDICTION     genemark        1
TRANSCRIPT      PASA    5
PROTEIN exonerate       3
```

847 EVM transcript models that are supported by all three sources were extracted and used for training Augustus. Augustus hint files were created from the PASA models (partial exon, intron)m exonerate alignments (partial exon), repeat masked regions (non-genic).

Augustus was then ran on the masked genome with hints from RepeatMasker, Exonerate, and PASA

The following extrinsic.cfg setting:

```
[SOURCES]
PASA E RM

[GENERAL]
start 1 1 PASA 1 1 E 1 1 RM 1 1
stop 1 1 PASA 1 1 E 1 1 RM 1 1
tss 1 1 PASA 1 1 E 1 1 RM 1 1
tts 1 1 PASA 1 1 E 1 1 RM 1 1
ass 1 1 PASA 1 1 E 1 1 RM 1 1
dss 1 1 PASA 1 1 E 1 1 RM 1 1
exonpart 1 1 PASA 1 1e3 E 1 1 RM 1 1
exon 1 1 PASA 1 1 E 1 1 RM 1 1
intronpart 1 1 PASA 1 5e3 E 1 1e20 RM 1 1
intron 1 1 PASA 1 1 E 1 1 RM 1 1
CDSpart 1 1 PASA 1 1 E 1 1e50 RM 1 1
CDS 1 1 PASA 1 1 E 1 1 RM 1 1
UTRpart 1 1 PASA 1 1 E 1 1 RM 1 1
UTR 1 1 PASA 1 1 E 1 1 RM 1 1
irpart 1 1 PASA 1 1 E 1 1 RM 1 1
nonexonpart 1 1 PASA 1 1 E 1 1 RM 1 1.15
genicpart 1 1 PASA 1 1 E 1 1 RM 1 1
```

In [2]:

```
prefix = "/home/share/projects/phaw_genome/draft_3.0/augustus/"
```

In [559]:

```
protLengths = {}
from Bio import SeqIO
protFile = open(prefix + 'phaw_30.augustus.prot.fa')
for record in SeqIO.parse(protFile,'fasta'):
    protLengths[record.id] = len(str(record.seq))
```

In [555]:

```
from collections import defaultdict
metaFile = open(prefix + 'augustus.meta')

metas = defaultdict(lambda : defaultdict(int))
supported = {}

for line in metaFile:
    meta = line.strip().split('\t')
    if meta[1] == 'hint percentage':
        supported[meta[0]] = float(meta[2])
    elif meta[1] == 'groups':
        metas[meta[0]][meta[2]] += int(meta[3])
```

In [560]:

```
uniprotHits = set(open(prefix + 'phaw_30.augustus.cds.uniprot.tids').read().strip().split())
```

In [561]:

```
print 'Number of Augustus predicted genes:', commas(len(supported))
print 'Number of Augustus predicted genes with exonerate evidence:', \
commas(len([g for g,d in metas.items() if d.has_key('E')]))
print 'Number of Augustus predicted genes with PASA evidence:', \
commas(len([g for g,d in metas.items() if d.has_key('PASA')]))
print 'Number of Augustus predicted genes with Uniprot hit:', \
commas(len(uniprotHits))
print 'Number of Augustus predicted genes with PASA/Exonerate evidence:', \
commas(len([g for g,d in metas.items() if d.has_key('E') or d.has_key('PASA')]))
print 'Number of Augustus predicted genes with Uniprot/PASA/Exonerate evidence and AA length >= 50:', \
commas(len([g for g,d in metas.items() if (g in uniprotHits or d.has_key('E') or d.has_key('PASA')) and (protLengths[g + '.transcript'] >= 50)]))
```

```
Number of Augustus predicted genes: 49,135
Number of Augustus predicted genes with exonerate evidence: 6,384
Number of Augustus predicted genes with PASA evidence: 18,982
Number of Augustus predicted genes with Uniprot hit: 25,870
Number of Augustus predicted genes with PASA/Exonerate evidence: 22,601
Number of Augustus predicted genes with Uniprot/PASA/Exonerate evidence and AA length >= 50: 28,224
```

### Filter for low complexity genes

Filter genes with mostly Ns or low complexity.

In [168]:

```
#Remove any sequence containing a k-mer (k-length 3 - 11) that occurs at a frequency > 50% of the sequence
def kmers(seq):
    for k in range(3,11):
        kfreq = defaultdict(int)
        for i in range(0,len(seq) - k,k):
            kmer = seq[i:i + 3]
            kfreq[kmer] += 1
        kfreq = kfreq.items()
        kfreq.sort(key = lambda x : x[-1], reverse = True)
        repeatPercent = kfreq[0][1] * k / float(len(seq))
        if repeatPercent >= 0.5:
            return True
        
    return False
```

In [564]:

```
cdsFile = open('/home/share/projects/phaw_genome/draft_3.0/augustus/phaw_30.augustus.keep.cds.fa')
from Bio import SeqIO
lowcomplex = []
for record in SeqIO.parse(cdsFile,'fasta'):
    seq = str(record.seq).upper()
    if kmers(seq):
        lowcomplex.append(record.id)
print 'Low complexity genes removed:', len(lowcomplex)
```

```
Low complexity genes removed: 69
```

### Final list of gene predictions

In [582]:

```
finalgids = [g for g,d in metas.items() if (g in uniprotHits or d.has_key('E') or d.has_key('PASA')) and (protLengths[g + '.transcript'] >= 50)]
```

In [584]:

```
finalgids = [x for x in finalgids if x + '.transcript' not in lowcomplex]
```

In [586]:

```
print 'Number of genes:', len(finalgids)
```

```
Number of genes: 28155
```

### Annotation stats based on gene prediction

In [158]:

```
genomeFile = open('/home/share/projects/phaw_genome/draft_3.0/assembly/phaw_30.fa')
total = 0
gcs = 0
for header in genomeFile:
    seq = genomeFile.next().strip().upper()
    GC = 0
    GC += seq.count('G')
    GC += seq.count('C')
    l = len(seq)
    l -= seq.count('N')
    total += l
    gcs += GC
```

In [159]:

```
print 'GC% of genome:', str(float(gcs) / total)
```

```
GC% of genome: 0.407909607488
```

In [589]:

```
cdsFile = open('/home/share/projects/phaw_genome/draft_3.0/anno/phaw_30.augustus.cds.fa')
total = 0
gcs = 0
cdsLengths = []
for record in SeqIO.parse(cdsFile,'fasta'):
    seq = str(record.seq).strip().upper()
    GC = 0
    GC += seq.count('G')
    GC += seq.count('C')
    l = len(seq)
    cdsLengths.append(l)
    l -= seq.count('N')
    total += l
    gcs += GC
```

In [590]:

```
print 'Total coding region bases:', commas(total)
print 'Percentage of genome coding:', float(total) / 3793719075 * 100
print 'GC% of coding region:', str(float(gcs) / total)
print 'Avg CDS length:', float(sum(cdsLengths)) / len(cdsLengths)
```

```
Total coding region bases: 19,335,382
Percentage of genome coding: 0.509668260031
GC% of coding region: 0.536932396784
Avg CDS length: 687.895364944
```

In [591]:

```
gffFile = open('/home/share/projects/phaw_genome/draft_3.0/augustus/phaw_30.augustus.final.gff')

geneLength = defaultdict(int)
introns = defaultdict(int)
cds = defaultdict(list)
for line in gffFile:
    meta = line.strip().split('\t')
    if meta[2] == 'gene':
        start = int(meta[3])
        end = int(meta[4])
        tid = meta[-1].split(';')[-1].split('=')[-1]
        geneLength[tid] = end - start + 1
    elif meta[2] == 'intron':
        start = int(meta[3])
        end = int(meta[4])
        tid = meta[-1].split(';')[-1].split('=')[-1]
        introns[tid] += end - start + 1
    elif meta[2] == 'CDS':
        start = int(meta[3])
        end = int(meta[4])
        tid = meta[-1].split(';')[-1].split('=')[-1]
        cds[tid].append(end - start + 1)
```

In [592]:

```
print 'avg gene length:', float(sum(geneLength.values())) / len(geneLength)
print 'avg CDS length:', float(sum([sum(v) for k,v in cds.items()])) / len(cds)
print 'avg exons per gene:', float(sum([len(v) for k,v in cds.items()])) / len(cds)
print 'avg exon length:', float(sum([sum(v) for k,v in cds.items()])) / float(sum([len(v) for k,v in cds.items()]))
```

```
avg gene length: 5070.18643225
avg CDS length: 687.977836974
avg exons per gene: 3.30001775884
avg exon length: 208.477010505
```

### Coverage

In [596]:

```
covFile = open('/home/share/projects/phaw_genome/draft_3.0/coverage/phaw.fixmate.sorted.realigned.duplicates.cov.categories.histo')

categories = defaultdict(list)
for line in covFile:
    meta = line.strip().split('\t')
    categories[meta[0]].append([int(meta[1]),int(meta[2])])
```

In [864]:

```
def plotCov(l, lower, upper, size, ax):
    total = sum([x[1] for x in l])
    df = pd.DataFrame([[x[0],x[1] / float(total)] for x in l])
    df.columns = ['coverage','bases']
    df[df['coverage'] >= lower][df['coverage'] <= upper]\
    .plot(x = 'coverage', y = 'bases',figsize=size,fontsize=16,ax=ax,lw=2)
```

In [598]:

```
uniprot_gids = set(open('/home/share/projects/phaw_genome/draft_3.0/anno/blast/phaw_30.uniprot.gid').read().strip().split())
```

In [648]:

```
geneCovs = []
for c, data in categories.items():
    if len(c.split('-')) == 2:
        if c.split('-')[1] == 'CDS':
            sCov = 0
            totalBases = 0
            for item in data:
                sCov += item[0] * item[1]
                totalBases += item[1]
            avgCov = sCov / float(totalBases)
            geneCovs.append((avgCov,c.split('-')[0]))
knownCovs = pd.Series([x[0] for x in geneCovs if x[1] in uniprot_gids])
allCovs = pd.Series([x[0] for x in geneCovs])
```

In [600]:

```
intronCovs = []
intronLengths = {}
for c, data in categories.items():
    if len(c.split('-')) == 2:
        if c.split('-')[1] == 'intron':
            sCov = 0
            totalBases = 0
            for item in data:
                sCov += item[0] * item[1]
                totalBases += item[1]
            avgCov = sCov / float(totalBases)
            intronCovs.append((avgCov,c.split('-')[0]))
            intronLengths[c.split('-')[0]] = totalBases
intron_knownCovs = pd.Series([x[0] for x in intronCovs if x[1] in uniprot_gids])
intron_allCovs = pd.Series([x[0] for x in intronCovs])
```

In [652]:

```
collapsed_gids = set(open('/home/share/projects/phaw_genome/draft_3.0/assembly/contig/blast_collapsed/collapsed.gids').read().strip().split())
```

In [653]:

```
collapsedCovs = pd.Series([x[0] for x in geneCovs if x[1] in collapsed_gids])
```

In [691]:

```
allCovs[allCovs <= 200].hist(bins=100,figsize=[14,7],color='#feb24c')
knownCovs[knownCovs <= 200].hist(bins=100,color='#c994c7').legend(['All genes','Genes with \nUniprot hit'],loc=2,prop={'size':14})
```

Out[691]:

```
<matplotlib.legend.Legend at 0x8e8dd2d0>
```

In [751]:

```
fig = plt.figure() # Create matplotlib figure
ax = fig.add_subplot(111) # Create matplotlib axes

plot_all = plotCov(categories['all'],0,200,[14,7],ax)
plot_intron = plotCov(categories['intron'],0,200,[14,7],ax)
plot_CDS = plotCov(categories['CDS'],0,200,[14,7],ax)
ax.legend(['All bases','Intronic bases','Coding bases'],prop={'size':14})
```

Out[751]:

```
<matplotlib.legend.Legend at 0xa1918b10>
```

In [760]:

```
print 'Sum bases less than 94 coverage:', commas(sum(x[1] for x in categories['all'][:94]))
print 'Sum bases more than or equal to 94 coverage:', commas(sum(x[1] for x in categories['all'][94:]))
```

```
Sum bases less than 94 coverage: 1,785,061,203
Sum bases more than or equal to 94 coverage: 885,881,248
```

In [601]:

```
fig = plt.figure() # Create matplotlib figure
ax = fig.add_subplot(111) # Create matplotlib axes
ax2 = ax.twinx() # Create another axes that shares the same x-axis as ax.

cov_all = allCovs[allCovs <= 200].hist(bins=100,figsize=[14,7],ax=ax,color='#feb24c')
cov_uniprot = knownCovs[knownCovs <= 200].hist(bins=100,ax=ax,color='#c994c7')

plot_all = plotCov(categories['all'],0,200,[14,7],ax2)
plot_intron = plotCov(categories['intron'],0,200,[14,7],ax2)
plot_CDS = plotCov(categories['CDS'],0,200,[14,7],ax2)

ax.legend(['All genes','Genes with \nUniprot hit'],loc=2,prop={'size':14})
ax2.legend(['All bases','Intronic bases','Coding bases'],prop={'size':14})

ax.set_xlabel('Coverage',fontsize=14)
ax.set_ylabel('Number of genes',fontsize=14)
ax2.set_ylabel('Density',fontsize=14)
plt.title('Coverage distribution of bases and genes',fontsize=20)
plt.show()
```

In [1138]:

```
print 'Number of genes <= 75 (heterozygous):', commas(len([x[0] for x in geneCovs if x[0] <= 75]))
print 'Number of genes > 75 (homozygous):', commas(len([x[0] for x in geneCovs if x[0] > 75]))
```

```
Number of genes <= 75 (heterozygous): 12,945
Number of genes > 75 (homozygous): 15,210
```

### Generate a canonical proteome for down-stream analysis

The gene predictions are very fragmented due to the fragmented genome assembly. There is also an over-prediction of genes because of alternate alleles. To generate a final list of genes for down-stream analysis, both the transcriptome and gene predictions are used:

TransDecoder was first ran on the transcriptome to get likely coding proteins. TransDecoder can predict multiple ORFs on the same transcript using hexamer frequency, PFAM annotations, ORF length.

There are cases where multiple ORFs were predicted on the same transcript, possibly in different frames or coordinates. These cases can be:

```
1. Assembler falsely fused two separate transcripts together
2. False ORFs due to hexamer frequency or abnormally long coding frame
```

All ORFs were blasted to proteomes used in the orthology analysis. For transcripts with multiple ORFs:

```
1. If only a single ORF has a hit, then that hit is kept, the rest are discarded
2. If multiple ORFs have a hit:
    A. If the ORFs with blast hit do not intersect on the transcript, all ORFs are kept
    B. If the ORFs with blast hit do intersect, the ORF with the highest bitscore is kept
```

In []:

```
prefix = '/home/share/projects/phaw_genome/draft_3.0/finalGeneset/'
```

In [103]:

```
def intersectCoord(coords):
	formatCoords = []
	for coord in coords:
		coord = [int(coord[0]),int(coord[1])]

		formatCoords.append(('s',min(coord)))
		formatCoords.append(('e',max(coord)))

	formatCoords.sort(key = lambda x : x[0], reverse = True)
	formatCoords.sort(key = lambda x : x[1])
	
	count = 0
	posA = 0

	level = 1
	out = []
	for pos in formatCoords:
		if pos[0] == 's':
			count = 1
			level += 1
			posA = pos[1]
		if pos[0] == 'e':
			level -=1
			count -=1

		if count == 0:
			if level > 1:
				out.append((posA, pos[1], level))

	return out
```

In [175]:

```
pepFile = open(prefix + 'Trinity_AverofPatel_Feb2015.fasta.transdecoder.pep')
blastFile = open(prefix + 'phaw_tra.blastp')
tids = defaultdict(list)
pepLengths = {}

for record in SeqIO.parse(pepFile,'fasta'):
    meta = record.description.split()[-5:]
    name = meta[0]
    t = meta[1].split(':')[-1]
    l = int(meta[2].split(':')[-1])
    strand = meta[3][1:-1]
    tid = meta[-1].split(':')[0]
    coords = map(int,meta[-1].split(':')[-1].split('(')[0].split('-'))

    tids[tid].append((name,t,l,strand,coords))
    pepLengths[name] = l

pepFile.close()

topHit = defaultdict(float)
topHitID = defaultdict(str)

for line in blastFile:
    meta = line.strip().split('\t')
    if float(meta[-1]) > topHit[meta[0]]:
        topHit[meta[0]] = float(meta[-1])
        topHitID[meta[0]] = meta[1]
blastFile.close()
```

In [129]:

```
discards = []
multiORF = 0
multiORFSingleHit = 0
multiORFNoOv = 0
multiORFOV= 0
for tid, orfs in tids.items():
    if len(orfs) > 1:
        multiORF += 1
        withHit = [orf for orf in orfs if topHit.has_key(orf[0])]
        if len(withHit) > 1:
            ovs = intersectCoord([c[-1] for c in withHit])
            if len(ovs) > 0:
                multiORFOV += 1
                ovORFs = [(orf,topHit[orf[0]]) for orf in orfs]
                ovORFs.sort(key = lambda x : x[1], reverse = True)
                discards.extend([x[0][0] for x in ovORFs[1:]])
            else:
                multiORFNoOv += 1
        else:
            multiORFSingleHit += 1
            discards.extend([orf[0] for orf in orfs if not topHit.has_key(orf[0])])
```

In [131]:

```
print 'Number of transcripts with coding potential (TransDecoder):', len(tids)
print 'Number of ORFs total predicted:', sum([len(v) for v in tids.values()])
print
print 'Number of transcripts with multiple ORFs predicted:', multiORF
print 'Number of transcripts with multiple ORFs where only one ORF has a blast hit:', multiORFSingleHit
print 'Number of transcripts with multiple ORFs where ORFs with blast hits do not overlap:', multiORFNoOv
print 'Number of transcripts with multiple ORFs where ORFs with blast hits do overlap:', multiORFOV
print
print 'Number of ORFs discarded:', len(discards)
```

```
Number of transcripts with coding potential (TransDecoder): 28522
Number of ORFs total predicted: 31157

Number of transcripts with multiple ORFs predicted: 2199
Number of transcripts with multiple ORFs where only one ORF has a blast hit: 1334
Number of transcripts with multiple ORFs where ORFs with blast hits do not overlap: 621
Number of transcripts with multiple ORFs where ORFs with blast hits do overlap: 244

Number of ORFs discarded: 2037
```

The remaining TransDecoder predicted ORFs were concatenated with 3,455 Augustus gene predictions that do not have transcriptome evidence. CD-HIT was ran on the resulting set of peptides to reduce redundancy.

```
cd-hit -i in.fasta -c 0.99 -o out.cdhit.fasta
```

GFF annotations of 30,930 peptides after CD-HIT was parsed for overlapping CDS features.

Rules for discarding peptides:

```
"TR" = peptide from transcriptome source
"AU" = peptide from gene prediction source

If multiple peptides overlap on the genome:
    If TRs overlap with AUs, keep only TRs
    else:
        if multiple TR overlaps:
            if only a single TR has a hit, keep that one
            else if multiple TRs has hits:
                if TRs all come from the same Trinity gene, keep the longest isoform
                else:
                    If TRs all have the exact same top hit, keep the longest TR
                    else:
                        keep all TRs
```

In [162]:

```
cdsBed = open(prefix + 'phaw_30.aug.tra.cds.merged.bed')

ovCDS = set()

for line in cdsBed:
    meta = line.strip().split()
    tids = meta[-1].split(';')
    tids = list(set(tids))
    tids.sort()
    tids = tuple(tids)
    if len(tids) > 1:
        ovCDS.add(tids)

cdsBed.close()
```

In [179]:

```
discards2 = []
for ov in ovCDS:
    if len(ov) > 1:
        tra = [x for x in ov if x[:4] != 'phaw']
        pha = [x for x in ov if x[:4] == 'phaw']
        
        if len(tra) > 0 and len(pha) > 0:
            discards2.extend(pha)
        if len(tra) > 1:
            ovHits = [x for x in tra if topHit.has_key(x)]
            noHits = [x for x in ovHits if not topHit.has_key(x)]
            if len(ovHits) == 1:
                discards2.extend(noHits)
            if len(ovHits) > 1:
                trinGene = set(['_'.join(x.split('_')[:-1]) for x in ovHits])
                if len(trinGene) == 1:
                    isoLengths = [(pepLengths[x],x) for x in ovHits]
                    isoLengths.sort(key = lambda x : x[0], reverse = True)
                    discards2.extend([x[1] for x in isoLengths[1:]])
                else:
                    th = set([topHitID[x] for x in ovHits])
                    if len(th) == 1:
                        tLengths = [(pepLengths[x],x) for x in ovHits]
                        tLengths.sort(key = lambda x : x[0], reverse = True)
                        discards2.extend([x[1] for x in tLengths[1:]])
```

In [186]:

```
print "Number of peptides discarded due to genomic coordinate overlap:", len(set(discards2))
print 'Number of total sequences: 28,666'
print
print 'Number of sequences from transcriptome source: 26,715'
print 'Number of sequences from gene prediction source: 1,951'
print 'Number of transcriptome source sequences mapped: 25,339'
```

```
Number of peptides discarded due to genomic coordinate overlap: 2264
Number of total sequences: 28,666

Number of sequences from transcriptome source: 26,715
Number of sequences from gene prediction source: 1,951
Number of transcriptome source sequences mapped: 25,339
```

### Cannonical proteome length stats

P. hawaiensis genes contains genes with large introns, similar to humans. The following shows the ratio of gene lengths to another species based on best BLAST hit. On average, P. hawaiensis genes have a ratio of >1 with humans. whereas D.pulex, D.melanogaster, and C.elegans contain genes that are around half as short as P.hawaiensis

In [157]:

```
def plotGeneLength(path):
    f = open(path)
    gLengths = []
    for line in f:
        meta = line.strip().split()
        gLengths.append(int(meta[1]))
    f.close()
    
    gLengths = pd.Series(gLengths)
    print 'Mean length:', gLengths.mean()
    print 'Median length:', gLengths.median()
    gLengths[gLengths < 50000].hist(bins=50)
    
def plotIntronProp(path):
    f = open(path)
    prop = []
    for line in f:
        meta = line.strip().split()
        prop.append(int(meta[2]) / float(meta[1]))
    f.close()
    
    prop = pd.Series(prop)
    prop.plot(kind='kde',xlim=[0,1],figsize=[10,6])
    
def plotOrthoRatio(path):
    f = open(path)
    ratio = []
    for line in f:
        if line[0] != '#':
            meta = line.strip().split()
            ratio.append(float(meta[2]) / float(meta[0]))
    ratio = pd.Series(ratio)
    return ratio
```

In [106]:

```
hsapRatio = plotOrthoRatio('/home/share/projects/phaw_genome/draft_3.0/finalGeneset/comp/phaw_hsap.mean.data')
dpulRatio = plotOrthoRatio('/home/share/projects/phaw_genome/draft_3.0/finalGeneset/comp/phaw_dpul.mean.data')
dmelRatio = plotOrthoRatio('/home/share/projects/phaw_genome/draft_3.0/finalGeneset/comp/phaw_dmel.mean.data')
celeRatio = plotOrthoRatio('/home/share/projects/phaw_genome/draft_3.0/finalGeneset/comp/phaw_cele.mean.data')
```

In [111]:

```
print 'Mean gene length ratio to h. sapiens top hit:', hsapRatio.mean()
print 'Mean gene length ratio to d. pulex top hit:', dpulRatio.mean()
print 'Mean gene length ratio to d. melanogaster top hit:', dmelRatio.mean()
print 'Mean gene length ratio to c. eleganstop hit:', celeRatio.mean()
```

```
Mean gene length ratio to h. sapiens top hit: 8.49287205033
Mean gene length ratio to d. pulex top hit: 0.437407172321
Mean gene length ratio to d. melanogaster top hit: 0.605835171517
Mean gene length ratio to c. eleganstop hit: 0.491961419939
```

In [138]:

```
orthoRatio = pd.concat([hsapRatio,dpulRatio,dmelRatio,celeRatio], ignore_index=True, axis=1)
orthoRatio.columns = ['H.sapiens','D.pulex','D.melanogaster','C.elegans']
```

In [167]:

```
print 'Tophit gene length ratio compared to P. hawaiensis'
plt.figure(figsize=(10, 6))
ax = sns.boxplot(data = orthoRatio)
ax.set_yscale('log')
```

```
Tophit gene length ratio compared to P. hawaiensis
```

In [152]:

```
hsapIntron = pd.Series(map(int,open('/home/share/projects/phaw_genome/draft_3.0/finalGeneset/comp/hsap.intronLengths')\
.read().strip().split('\n')))
dpulIntron = pd.Series(map(int,open('/home/share/projects/phaw_genome/draft_3.0/finalGeneset/comp/dpul.intronLengths')\
.read().strip().split('\n')))
dmelIntron = pd.Series(map(int,open('/home/share/projects/phaw_genome/draft_3.0/finalGeneset/comp/dmel.intronLengths')\
.read().strip().split('\n')))
celeIntron = pd.Series(map(int,open('/home/share/projects/phaw_genome/draft_3.0/finalGeneset/comp/cele.intronLengths')\
.read().strip().split('\n')))
phawIntron = pd.Series(map(int,open('/home/share/projects/phaw_genome/draft_3.0/finalGeneset/comp/phaw.intronLengths')\
.read().strip().split('\n')))

intronLengths = pd.concat([hsapIntron,dpulIntron,celeIntron,dmelIntron,phawIntron], ignore_index=True, axis=1)
intronLengths.columns = ['H.sapiens','D.pulex','D.melanogaster','C.elegans','P.hawaiensis']
```

In [164]:

```
for sp in ['H.sapiens','D.pulex','D.melanogaster','C.elegans','P.hawaiensis']:
    print sp + ' mean intron size:', intronLengths[sp].mean()
    print sp + ' median intron size:', intronLengths[sp].median()
    print
```

```
H.sapiens mean intron size: 5905.59127597
H.sapiens median intron size: 1542.0

D.pulex mean intron size: 285.859626795
D.pulex median intron size: 77.0

D.melanogaster mean intron size: 317.977964928
D.melanogaster median intron size: 66.0

C.elegans mean intron size: 1073.5559806
C.elegans median intron size: 71.0

P.hawaiensis mean intron size: 5478.85799714
P.hawaiensis median intron size: 1843.0
```

In [166]:

```
print 'Intron Length distributions'
plt.figure(figsize=(10, 6))
ax = sns.boxplot(data = intronLengths)
ax.set_yscale('log')
```

```
Intron Length distributions
```

In [158]:

```
print 'Histogram of p.hawaiensis gene lengths'
plotGeneLength('/home/share/projects/phaw_genome/draft_3.0/finalGeneset/comp/phaw.lengths.data')
```

```
Histogram of p.hawaiensis gene lengths
Mean length: 20005.3370836
Median length: 7221.0
```

In [159]:

```
print 'Histogram of h.sapiens gene lengths'
plotGeneLength('/home/share/projects/phaw_genome/draft_3.0/finalGeneset/comp/hsap.lengths.data')
```

```
Histogram of h.sapiens gene lengths
Mean length: 52943.0267925
Median length: 18537.0
```

In [160]:

```
print 'Histogram of d.pulex gene lengths'
plotGeneLength('/home/share/projects/phaw_genome/draft_3.0/finalGeneset/comp/dpul.lengths.data')
```

```
Histogram of d.pulex gene lengths
Mean length: 2016.73396535
Median length: 1173.0
```

In [161]:

```
print 'Histogram of c.elegans gene lengths'
plotGeneLength('/home/share/projects/phaw_genome/draft_3.0/finalGeneset/comp/cele.lengths.data')
```

```
Histogram of c.elegans gene lengths
Mean length: 2829.15263853
Median length: 1791.0
```

In [162]:

```
print 'Histogram of d.melanogaster gene lengths'
plotGeneLength('/home/share/projects/phaw_genome/draft_3.0/finalGeneset/comp/dmel.lengths.data')
```

```
Histogram of d.melanogaster gene lengths
Mean length: 4737.00804713
Median length: 1557.0
```
