# Supplementary material for: The genome of the crustacean Parhyale hawaiensis, a model for animal development, regeneration, immunity and lignocellulose digestion
Source: eLife. 2016 Nov 16;5:e20062. doi: 10.7554/eLife.20062 (PMC5111886; doi:10.7554/eLife.20062)
Supplement: Source code 4. — Analysis of polymorphism in Parhyale using genome reads, transcriptome data and sanger sequenced BACs. DOI: http://dx.doi.org/10.7554/eLife.20062.049 [file elife-20062-code4.htm]

Notebook


In [1]:

```
import gzip, os, sys
from IPython.display import HTML
import pandas as pd
import seaborn as sns
import matplotlib.pyplot as plt
import scipy.stats as ss
import scipy as sp
import numpy as np

#import custom functions for displaying tables, bash commands
sys.path.append(os.path.abspath("/home/damian/"))
from dk_ipython import *
%matplotlib inline
HTML(addToggle())
```

Out[1]:

The raw code for this IPython notebook is by default hidden for easier reading.
To toggle on/off the raw code, click here.

# Variant analysis on genes

The goal of this analysis is to try to identify alternate alleles of heterozygous genic regions. This is done by mapping the proteome dataset to the genome and attempting to identify the multiple mapping loci as heterozygous alleles. Some caveats about this analysis:

- The genome is fragmented, meaning genes can potentially map to more than one reference scaffold because they span multiple scaffolds.
- If an alternate alellic scaffold does exist, it does not necessarily have to contain the entire gene. There are many cases where part of the gene is homozygous and another part is heterozygous, therefore mapping to multiple scaffolds.
- Genes that map to multiple loci can represent genome fragmentation, repetitive sequence, conserved domain, or heterozygosity.
- The decision for the genome assembler to assemble alleles separately is ultimately NOT based on the amount of heterozygosity. It is a function of the density of the heterozygosity. However, higher rate of heterozygosity will have some correlation with higher density, so there should be some relationship between heterozygosity rate and assembly of alternate allele.

There is no perfect method of teasing apart whether multiple mapping is heterozygous, conserved domain, fragmentation, or repetitive. There will likely be a false heterozygous bias for transcripts containing a very conserved domain or transcripts within a large gene family.

The strategy for trying to find variants of genic regions is to first align the transcripts to the genome assembly. For each transcript sequence, stacks of reference sequence alignment are mapped below it producing an alignment file:

```
>transcript
tccaacgaccactgctggcagccgaggatgccgtcagtaccgaaagagttcaccatcctctttgtacttatgaaag
tccaacgaccactgctggcagccgaggatgccgtcagtaccgaaagagttcaccatcctctttgtacttatg                          
                          gatgccgtcagtaccgaaagagttcaccatcctct-----cttatga
```

Where the first sequence is the transcript sequence. The rest of the sequences are reference alignments to the transcript, each representing one reference scaffold. The order of the alignments are sorted by number of matching bases. Alignments to reference scaffolds less than 30 bases are discarded. The top reference aligments theoretically should be the best alignment (let's call this the primary allele). If an alternate allele exist, there should be secondary alignments (secondary allele).

In the cases where the transcript maps across multiple scaffolds, the top N alignments represents the primary allele, and there may also be N alignments representing the secondary allele. In cases where there is a conserved domain or repetitive region, we will get many alignments apart from the primary or secondary alignments. There isn't a perfect way to distinguishing this scenario. We make the assumption that heterozygosity will be less variant than paralogs/conserved domain.

For each transcript base position, we can get a list of possible reference bases, if any exist at all. If there are at least 2 bases within this list, we compare the top two bases (alignments are sorted by number of matching bases, meaning top alignment has the most number of matching bases). If a position has more than 5 possible reference bases, then this position is ignored as potentially part of a conserved domain. If the bases don't match, we record this as a potential heterozygous bases. If the bases don't match the transcript, we record this as a possible population variant. For the sake of simplicity, we are ignoring indels.

Use Blat to align transcripts to the genome assembly:

```
blat -out=maf assembly.fa transcriptome.fa output.maf

example maf format entry:
a score=1396.000000
s phaw_30.0224235 401 17 +  530 cttggtccatttgtaaa
s c0_g1_i1        859 17 - 1003 cttggtccattggtaaa

sequence name, alignment start, alignment size, strand, sequence length, sequence alignment
```

Use the `simplify.py` script to format the .maf output:

```
python simplify.py output.maf > output.format.data

converts each alignment to:

phaw_30.0224235 530     401,418,+       c0_g1_i1        1003    859,876,-       17      cttggtccatttgtaaa       cttggtccattggtaaa       16      1       0       0       0

columns: reference, reference size, reference coordinates, transcript name, transcript size, transcript coordinates, alignment length, reference sequence, transcript sequence, matches, mismatches, reference deletions, reference insertions, number of Ns
```

Use the `process.py` script to convert the data into stacks of aligned sequences:

```
python process.py output.format.data > output.stacks
```

Analyze the stacks data for potential heterozygosity:

```
python analyzeHet.py output.stacks > output.hetRate
```

The output of this analysis is a tab delimited file showing:

```
geneID                  Length     Covered Bases   variant codes
phaw_30_tra_m.000004    5234       5075            2805:010,4518:010,4746:100

- Variant codes indicate the position and type of variant. 
- First number before the colon is the base position in the gene.
- The three numbers after are true/false flags indicating whether the variant is:
    heterozygous, different from transcript, is an indel
- 010 would indicate a homozygous variant that is different from the transcript sequence, and is a SNP
```

In [2]:

```
prefix = '/home/share/projects/phaw_genome/draft_3.0/heterozygosity/'
```

In [3]:

```
rateFile = open(prefix + 'phaw_30.genes.final.cds.blat.het_var.rate2.cov')
tids = []

for line in rateFile:
    meta = line.strip().split()

    tid = meta[0]
    length = float(meta[1])
    covLength = float(meta[2])
    cov = float(meta[-1])
    if covLength >= 200:
        hets = int(meta[3])
        varis = int(meta[4])
        hets_vars = int(meta[5])

        tids.append([tid, hets,varis, hets_vars, hets/covLength * 100,varis/covLength * 100,cov])

rateFile.close()
```

In [4]:

```
tids = pd.DataFrame(tids)
tids.columns = ['id','hets','vars','hets_vars','hetProp','varProp','coverage']
```

In [7]:

```
print 'Coverage vs heterozygosity for each gene'
tids[tids['coverage'] < 250]\
[tids['hetProp'] < 10]\
.plot(kind='scatter',x='coverage',y='hetProp',s=1,figsize=[10,6],alpha=0.5,xlim=[0,250],ylim=[0,10])
```

```
Coverage vs heterozygosity for each gene
```

Out[7]:

```
<matplotlib.axes.AxesSubplot at 0x53d0550>
```

In [17]:

```
unassigned = set(open('/home/share/projects/phaw_genome/draft_3.0/comp/blast2/phaw.specific.ids').read().strip().split('\n'))
```

In [20]:

```
tids[tids['coverage'] < 250][tids['id'].isin(unassigned)]['coverage'].hist(bins=100,figsize=[10,4])
```

Out[20]:

```
<matplotlib.axes.AxesSubplot at 0x7197150>
```

In [6]:

```
print 'Coverage distribution for each gene'
tids[tids['coverage'] < 250]['coverage'].hist(bins=100,figsize=[10,4])
```

```
Coverage distribution for each gene
```

Out[6]:

```
<matplotlib.axes.AxesSubplot at 0x595f390>
```

In [8]:

```
cov_het = pd.concat([tids[tids['hetProp'] <= 1]\
[tids['coverage'] < 250]\
['coverage'],
tids[tids['hetProp'] > 1]\
[tids['coverage'] < 250]\
['coverage']],ignore_index=True, axis=1)
cov_het.columns = ['Genes with het. rate <= 1.0%','Genes with het. rate > 1.0%']
cov_het.plot.hist(stacked = True,bins=100,figsize=[10,4])
```

Out[8]:

```
<matplotlib.axes.AxesSubplot at 0x5a490d0>
```

In [9]:

```
print 'Heterozygosity rate for genes segemented into two coverage ranges'
tids[tids['coverage'] < 250]\
[tids['coverage'] >= 105]\
['hetProp'].plot(kind='kde',xlim=[0,10])
tids[tids['coverage'] < 105]\
['hetProp'].plot(kind='kde', figsize=[10,6])
plt.legend(['Genes with coverage between 105-250','Genes with coverage below 105'])
```

```
Heterozygosity rate for genes segemented into two coverage ranges
```

Out[9]:

```
<matplotlib.legend.Legend at 0x6005490>
```

In [12]:

```
print 'Population variant rate for genes segemented into two coverage ranges'
tids[tids['coverage'] < 250]\
[tids['coverage'] >= 105]\
['varProp'].plot(kind='kde',xlim=[0,5])
tids[tids['coverage'] < 105]\
['varProp'].plot(kind='kde', figsize=[10,4])
plt.legend(['Genes with coverage between 105-250','Genes with coverage below 105'])
```

```
Population variant rate for genes segemented into two coverage ranges
```

Out[12]:

```
<matplotlib.legend.Legend at 0x6cbf190>
```

In [134]:

```
print 'Coverage vs population variance for each gene'
tids[tids['coverage'] < 250]\
.plot(kind='scatter',x='coverage',y='varProp',s=1,figsize=[10,6],alpha=0.5,xlim=[0,250],ylim=[0,5])
```

```
Coverage vs population variance for each gene
```

Out[134]:

```
<matplotlib.axes.AxesSubplot at 0xe4e7250>
```

In [184]:

```
print 'Rate = Number of SNPs divided by total genic bases assessed x 100' 
print
print 'Heterozygosity ratein genic regions (%):', sum(allhets) / totalCov * 100
print 'Population variant rate from transcript in genic regions (%):', sum(allvars) / totalCov * 100
print 'Heterozygosity + Pop. variant rate genic regions (%):', sum(allhets_vars) / totalCov * 100
print
print 'Heterozygosity rate of genes between 105 and 250 coverage:', \
tids[tids['coverage'] < 250][tids['coverage'] >= 105]['hetProp'].mean()
print 'Heterozygosity rate of genes under 105 coverage:', \
tids[tids['coverage'] < 105]['hetProp'].mean()
print
print 'Heterozygosity rate of genes between 105 and 250 coverage:', \
tids[tids['coverage'] < 250][tids['coverage'] >= 105]['varProp'].mean()
print 'Heterozygosity rate of genes under 105 coverage:', \
tids[tids['coverage'] < 105]['varProp'].mean()
```

```
Rate = Number of SNPs divided by total genic bases assessed x 100

Heterozygosity ratein genic regions (%): 1.51325481755
Population variant rate from transcript in genic regions (%): 0.661967883827
Heterozygosity + Pop. variant rate genic regions (%): 0.0102653592214

Heterozygosity rate of genes between 105 and 250 coverage: 1.09453811623
Heterozygosity rate of genes under 105 coverage: 2.68429591739

Heterozygosity rate of genes between 105 and 250 coverage: 0.875769993576
Heterozygosity rate of genes under 105 coverage: 0.731853832037
```

# Heterozygosity in BAC clones

ShortSGL libraries were mapped back to the BAC sequences individually. Called SNPs with a coverage greater than 250 were discarded as potentially conserved mapped regions (for example HOX domains).

The following shows SNP variant rates for heterozygosity and population variation.

In [206]:

```
bacTable = ListTable()
bacTable.append(['bac','length','heterozygosity','population variants','heterozygosity + population variants'])
bacFile = open(prefix + 'bac.intergenic.rate')

for line in bacFile:
    meta = line.strip().split()

    bid = meta[0]
    length = float(meta[1])
    covLength = float(meta[2])
    cov = int(meta[2]) / length
    hets = int(meta[3])
    varis = int(meta[4])
    hets_vars = int(meta[5])
    
    bacTable.append([bid,length,hets / length * 100, varis / length * 100, hets_vars / length * 100])
    

bacFile.close()
```

In [207]:

```
bacTable
```

Out[207]:

|  |  |  |  |  |
| --- | --- | --- | --- | --- |
| bac | length | heterozygosity | population variants | heterozygosity + population variants |
| PA81-D11 | 140264.0 | 1.65473678207 | 0.568214224605 | 0.000712941310671 |
| PA40-O15 | 129957.0 | 2.4469632263 | 0.647906615265 | 0.0207761028648 |
| PA76-H18 | 141844.0 | 1.82453963509 | 0.199514960097 | 0.002819999436 |
| PA120-H17 | 126766.0 | 2.67342978401 | 1.12096303425 | 0.0197213763943 |
| PA222-D11 | 128542.0 | 1.3443076971 | 1.40421029702 | 0.017115028551 |
| PA31-H15 | 140143.0 | 2.79357513397 | 0.0513760944178 | 0.00285422746766 |
| PA284-I07 | 141390.0 | 2.04611358653 | 0.45052691138 | 0.0127307447486 |
| PA221-A05 | 148703.0 | 1.86277344774 | 1.42700550762 | 0.0235368486177 |
| PA93-L04 | 139955.0 | 2.17712836269 | 0.742381479761 | 0.0135757922189 |
| PA272-M04 | 134744.0 | 1.92587425043 | 0.98260404916 | 0.0141008133943 |
| PA179-K23 | 137239.0 | 2.67198099666 | 0.990971954036 | 0.0298748897908 |
| PA92-D22 | 126848.0 | 2.65041624622 | 0.802535317861 | 0.0268037336024 |
| PA268-E13 | 135334.0 | 1.67807055138 | 1.32265358299 | 0.0221673784858 |
| PA264-B19 | 108571.0 | 1.5759272734 | 0.157500621713 | 0.00552633760396 |
| PA24-C06 | 141446.0 | 1.94632580631 | 1.48820044399 | 0.0113117373415 |

### Contiguous regions of overlapping BAC sequences

SNPs called from the previous mapping shortSGL libraries to individual BACs was used to create multiple variant BACs. No phasing information is not required since we are just interested in the potential variants at each position. The possible variant BACs according to SNPs called by reads and the BAC sequences were aligned with BLAT to get coordinates of the overlapping regions.

The overlapping regions were then extracted out of each BAC and the corresponding read variant BACs for multiple sequence alignment with clustalw. The alignment file was then parsed for SNPs at each position. SNPs were categorized by being BAC supported, reads supported, BAC and reads supported, and presence of an insertion/deletion.

In [28]:

```
from collections import defaultdict
inFile = open('/home/share/projects/phaw_genome/draft_3.0/bac/ov/clustal2/ov.data','r')

inFile.next()

ovCount = defaultdict(lambda : [0,0,0,0])

for line in inFile:
    ov,pos,bac,bac_reads,reads,indel = line.strip().split()
    pos = int(pos)
    bac = int(bac)
    bac_reads = int(bac_reads)
    reads = int(reads)
    indel = int(indel)


    if indel == 0:
        if bac == bac_reads == reads:
            ovCount[ov][1] += 1

        if bac > bac_reads:
            ovCount[ov][0] += 1

        if reads > bac_reads:
            ovCount[ov][2] += 1
    else:
        ovCount[ov][3] += 1

ovTable = ListTable()
ovTable.append(['Overlaps','BAC only SNPs','BAC and read SNPs','read only SNPs','INDELs'])
for ov, count in ovCount.items():
    meta = ov.split('.')
    name = meta[0] + ' - ' + meta[1].split('_')[1]
    ovTable.append([name] + count)
ovTable
```

Out[28]:

|  |  |  |  |  |
| --- | --- | --- | --- | --- |
| Overlaps | BAC only SNPs | BAC and read SNPs | read only SNPs | INDELs |
| PA81-D11 - PA272-M04 | 1 | 841 | 13 | 733 |
| PA272-M04 - PA92-D22 | 8 | 0 | 1269 | 745 |
| PA92-D22 - PA284-I07 | 2 | 0 | 206 | 61 |
| PA264-B19 - PA179-K23 | 1 | 0 | 425 | 484 |
| PA40-O15 - PA81-D11 | 5 | 539 | 365 | 2056 |
| PA284-I07 - PA221-A05 | 2 | 120 | 513 | 322 |
| PA221-A05 - PA76-H18 | 0 | 395 | 146 | 1828 |
| PA179-K23 - PA40-O15 | 1 | 88 | 33 | 286 |

### Position and length of Indels in BACs

In [55]:

```
from itertools import groupby
from operator import itemgetter

inFile = open('/home/share/projects/phaw_genome/draft_3.0/bac/ov/clustal2/ov.data','r')

indels = defaultdict(list)
inFile.next()
for line in inFile:
        meta = line.strip().split()
        if meta[5] == '1':
                indels[meta[0]].append(int(meta[1]))

for ov, pos in indels.items():
        pos.sort()

        ranges = []
        for key, group in groupby(enumerate(pos), lambda (index, item): index - item):
                group = map(itemgetter(1), group)
                if len(group) > 1:
                        ranges.append(range(group[0], group[-1]))
                else:
                        ranges.append([group[0]])

        d = [(x[0],len(x)) for x in ranges if len(x) > 1]

        d = pd.DataFrame(d)
        d.columns = ['position','indel length']
        
        meta = ov.split('.')
        
        d.plot(kind='scatter',x='position',y='indel length',s=10,title=meta[0] + ' - ' + meta[1].split('_')[1],figsize=[6,2])
        plt.ylim(ymin=0)
        plt.xlim(xmin=0)
```
