# Supplementary material for: The genome of the crustacean Parhyale hawaiensis, a model for animal development, regeneration, immunity and lignocellulose digestion
Source: eLife. 2016 Nov 16;5:e20062. doi: 10.7554/eLife.20062 (PMC5111886; doi:10.7554/eLife.20062)
Supplement: Source code 5. — Protein orthology analysis between Parhyale and other species DOI: http://dx.doi.org/10.7554/eLife.20062.050 [file elife-20062-code5.htm]

Notebook


In [70]:

```
import gzip, os, sys
from IPython.display import HTML
import pandas as pd
import seaborn as sns
import matplotlib.pyplot as plt
import scipy.stats as ss
import scipy as sp

#import custom functions for displaying tables, bash commands
sys.path.append(os.path.abspath("/home/damian/"))
from dk_ipython import *
%matplotlib inline
HTML(addToggle())
```

Out[70]:

The raw code for this IPython notebook is by default hidden for easier reading.
To toggle on/off the raw code, click here.

# Orthology analysis

20 species were used to perform orthology analysis with Parhyale Hawaiensis (amphipod crustacean). Proteomes were downloaded from Uniprot. TransDecoder was ran on transcriptomes to get likely protein sequences. Calanus finmarchicus seems to have a lot of redundancies in the transcriptome which might result in artificially larger number of paralogs.

```
Parhyale hawaiensis (amphipod) (transcriptome)
Litopenaeus vannamei (decapod) (transcriptome)
Daphnia pulex (branchiopod)
Echinogammarus veneris (amphipod) (transcriptome)
Eucyclops serrulatus (copepod) (transcriptome)
Calanus finmarchicus (copepod) (transcriptome)
Lepeophtheirus salmonis (copepod)
Speleonectes tulumensis (remipedia) (transcriptome)

Drosophila melanogaster (hexapod)
Aedes gambiae (hexapod)

Strigamia maritima (Myriapod)
Mesobuthus martensii (Chelicerata)
Stegodyphus mimosarum (Chelicerata)
Ixodes scapularis (Chelicerata)

Hypsibius dujardini (tardigrada)

Caenoerhabiditis elegans
Brugia malayi
Trichinella spiralis

Mus musculus
Homo sapiens
Branchiostoma floridae
```

OrthoFinder (https://github.com/davidemms/OrthoFinder) was used for the orthology analysis. Formatting of the files and blast were done manually so we can run the blasts on cluster.

Each fasta file had to be renamed to Species#.fa and a SpeciesID.txt index file generated to indicate the number and species. My SpeciesID.txt file:

```
0: agam.proteome.fa
1: dmel.proteome.fa
2: stul.transdecoder.fa
3: dpul.proteome.fa
4: phaw.transdecoder.fa
5: even.transdecoder.fa
6: lvan.transdecoder.fa
7: eser.transdecoder.fa
8: cfin.transdecoder.fa
9: smar.proteome.fa
10: smim.proteome.fa
11: mmar.proteome.fa
12: isca.proteome.fa
13: hdju.proteome.fa
14: bmal.proteome.fa
15: cele.proteome.fa
16: hsap.proteome.fa
17: mmus.proteome.fa
18: bflo.proteome.fa

`stul.transcriptome.fa.transdecoder.pep` would be renamed to `Species0.fa'
`smar.proteome.fa` would be renamed to `Species1.fa`, etc
```

Each entry in the fasta file needs to be renamed according to the species number and entry number. For example the first entry in `Species0.fa` would be renamed to:

```
>0_0
AGATAGACCCC....
>0_1
AGATAGAG...
```

A `SequenceIDs.txt` index file needs to be generated for each newly generated header name and the old header name. For example the first few lines of my `SequenceIDs.txt`:

```
0_0: gi|333236460|gb|JL099667.1||m.22240
0_1: gi|333236463|gb|JL099670.1||m.22239
0_2: gi|333236488|gb|JL099695.1||m.22237
0_3: gi|333236490|gb|JL099697.1||m.22235
0_4: gi|333236493|gb|JL099700.1||m.22233
```

Used the following script to do all the renaming:

```
import sys,os
from Bio import SeqIO
fs = [x for x in os.listdir(sys.argv[1])]

seqID = open(sys.argv[2],'w')
speID = open(sys.argv[3],'w')

for i,f in enumerate(fs):
        fName = 'Species' + str(i) + '.fa'

        inFile = open(sys.argv[1] + '/' + f)
        outFile = open(fName,'w')

        speID.write(str(i) + ': ' + f + '\n')
        seqCount = 0
        for record in SeqIO.parse(inFile,'fasta'):
                sid = str(i) + '_' + str(seqCount)
                rid = record.id

                seqID.write(sid + ': ' + rid + '\n')
                outFile.write('>' + sid + '\n' + str(record.seq) + '\n')

                seqCount += 1

        outFile.close()
        inFile.close()

seqID.close()
speID.close()
```

Move all your fasta files into an empty folder. Run the script by `python formatOrthoFinder.py fastaFolder SequenceIDs.txt SpeciesID.txt`. The script will generate a `Species#.fa` for each fasta file and write the corresponding indices files.

OrthoFinder wants pair-wise blasts for each of the Species. The instructions says you don't have to do all permutations, just combinations. Then for each combination switch the query and target columns to generate the reciprocal blast result.

For each pair-wise blast, OrthoFinder wants a `Blastx_y.txt` file. For example `Blast0_1.txt` would be Species0.fa blast to Species01.

It might also be possible to do an All vs All blast with all the sequences at once and then parse the results into individual pair-wise blast files. Since OrthoFinder uses bitscores instead of e-values, the size of alignment space do not matter.

We decided to run a series of pair-wise blasts. To generate the pair-wise blast commands (combinations, not permutations):

```
import sys
import itertools

# 17 species
r = range(17)

# combinations
for c in itertools.combinations(r,2):
    print 'blastp -query Species' + str(c[0]) + '.fa -db Species' + str(c[1]) + ' -outfmt 6 -evalue 0.0001 -out Blast' + str(c[0]) + '_' + str(c[1]) + '.txt'

# self blasts
for i in range(17):
    print 'blastp -query Species' + str(i) + '.fa -db Species' + str(i) + ' -outfmt 6 -evalue 0.0001 -out Blast' + str(i) + '_' + str(i) + '.txt'
```

After the combination are done running, move all the `Blastx_y.txt` results into a empty folder and use the following script to generate reciprocal blasts (the script just swaps the first two columns around):

```
import sys,os

fs = set([f.split('.')[0] for f in os.listdir(sys.argv[1])])

for f in fs:
    a, b = f[5:].split('_')
    recip = 'Blast' + str(b) + '_' + str(a)

    if not recip in fs:
        inFile = open(sys.argv[1] + '/' + f + '.txt')
        outFile = open(recip + '.txt','w')

        for line in inFile:
            meta = line.strip().split()
            swapped = [meta[1],meta[0]] + meta[2:]
            outFile.write('\t'.join(swapped) + '\n')

        inFile.close()
        outFile.close()
```

Use by `python genRecip.py folderName`

Move all blast reslts, SequenceIDs.txt, SpeciesIDs.txt, and Species#.fa files into one folder and run OrthoFinder:

```
OrthoFinder.py -b dataFolder
```

OrthoFinder ouputs a OrthologousGroups.txt file where each line is one group.

In [72]:

```
def plotCombo(cCount, spNames, c, t, atLeast = -1):
    a = getCombo(cCount, c, atLeast)
    if len(a) > 0:
        fig, ax = plt.subplots(figsize=[10,len(a)])
        ax.set_title(t)
        sns.heatmap(pd.DataFrame([x[0] for x in a], index = [x[1] for x in a],columns=spNames),linewidth=0.5,ax=ax)

def getCombo(cCount, c, atLeast):
    res = []
    for combo, count in cCount:
        check = True
        atlCount = 0
        for i in range(len(combo)):
            if c[i] == 0:
                if combo[i] == 1:
                    check = False
                    break
            if c[i] == 2:
                if combo[i] != 1:
                    check = False
                    break
            if c[i] == 1:
                if combo[i] == 1:
                    atlCount += 1
                    
        if atlCount < atLeast:
            check = False
            
        if check:
            res.append((combo,count))
            
    return res

def getInclusiveCombo(cCount, c):
    res = 0
    for combo, count in cCount:
        check = True
        for i in range(len(c)):
            if combo[i] == 1 and c[i] == 0:
                check = False
                break
        if check:
            res += count
            
    return res

def getPhawCount(pCount, c, atLeast):
    res = 0
    for combo, count in pCount.items():
        check = True
        atlCount = 0
        for i in range(len(combo)):
            if c[i] == 0:
                if combo[i] == 1:
                    check = False
                    break
            if c[i] == 2:
                if combo[i] != 1:
                    check = False
                    break
            if c[i] == 1:
                if combo[i] == 1:
                    atlCount += 1
                    
        if atlCount < atLeast:
            check = False
            
        if check:
            res += count
            
    return res

def getPhawIDs(pIndex, c, atLeast):
    res = []
    for combo, pids in pIndex.items():
        check = True
        atlCount = 0
        for i in range(len(combo)):
            if c[i] == 0:
                if combo[i] == 1:
                    check = False
                    break
            if c[i] == 2:
                if combo[i] != 1:
                    check = False
                    break
            if c[i] == 1:
                if combo[i] == 1:
                    atlCount += 1
                    
        if atlCount < atLeast:
            check = False
            
        if check:
            res.extend(pids)
            
    return res


def loadOrthoResult(prefix,spNames,refSpec):
    seqFile = open(prefix + 'SequenceIDs.txt','r')
    speFile = open(prefix + 'SpeciesIDs.txt','r')

    seqIndex = {}
    speIndex = {}

    for line in seqFile:
        meta = line.strip().split(':')
        seqIndex[meta[1].strip()] = meta[0].strip()

    refNum = -1
    for line in speFile:
        meta = line.strip().split(':')
        sp = meta[1].strip().split('.')[0]
        if sp == refSpec:
            refNum = meta[0].strip()
            
        speIndex[meta[0].strip()] = sp

    seqFile.close()
    speFile.close()
    
    orthoFile = open(prefix + 'OrthologousGroups.txt','r')
    speLength = len(speIndex)
    groups = []
    paralogCount = 0
    for line in orthoFile:
        meta = line.strip().split(':')
        tids = meta[1].strip().split()
        sids = [seqIndex[tid] for tid in tids]
        phawIDs = [tid for tid in tids if seqIndex[tid].split('_')[0] == refNum]
        spes = set([x.split('_')[0] for x in sids])
        if len(spes) > 1:
            groups.append((sids,phawIDs))
        else:
            paralogCount += 1

    orthoFile.close()

    combos = []
    for group, phawIDs in groups:
        spes = [x.split('_')[0] for x in group]
        combo = [(i,spes.count(str(i))) for i in range(speLength)]
        total = sum([x[1] for x in combo])
        phawTotal = spes.count(refNum)
        phawTids = []
        combos.append(([(x[0],x[1],float(x[1]) / total * 100) for x in combo],phawTotal,phawIDs))
        
    speOrder = range(speLength)
    
    comboData = []
    for combo, phawTotal,phawIDs in combos:
        comboString = []
        for i in speOrder:
            c = 0
            if combo[i][1] > 0:
                c = 1
            comboString.append(c)
        comboData.append((comboString, combo,phawTotal,phawIDs))

    from collections import defaultdict
    comboCount = defaultdict(int)
    phawCount = defaultdict(int)
    phawIDIndex = defaultdict(list)
    for combo in comboData:
        comboCount[tuple(combo[0])] += 1
        phawCount[tuple(combo[0])] += combo[2]
        phawIDIndex[tuple(combo[0])].extend(combo[3])
    comboCount = comboCount.items()
    comboCount.sort(key = lambda x : x[1],reverse = True)
    
    comboList = [(x[0],x[1]) for x in comboCount[:100]]
    comboList.sort(key = lambda x : ''.join(map(str,x[0])))
    
    orthoGrid = pd.DataFrame([x[0] for x in comboList])
    orthoGrid.columns = spNames
    
    return (spNames, comboCount, phawCount, phawIDIndex)

def getInfo(cCount,sps, pCount, combo, title, atLeast = -1):
    c = getCombo(cCount,combo,atLeast)
    print title
    print 'Number of total groups:', sum([x[1] for x in c])
    print 'Number of inclusive groups:', getInclusiveCombo(cCount, combo)
    print 'Number of P.haw genes:', getPhawCount(pCount,combo,atLeast)
    print
    plotCombo(cCount,sps,combo,title,atLeast)
```

In [85]:

```
specNames = ['A.gam','D.mel','S.tul','D.pul','P.haw',\
                         'E.ven','L.van','E.ser','C.fin','L.sal','S.mar','S.mim',\
                         'M.mar','I.sca','H.dju','B.mal','T.spi','C.ele','H.sap','M.mus','B.flo']
sps, cCount, pCount, pIndex = loadOrthoResult('/home/share/projects/phaw_genome/draft_3.0/comp/blast/',specNames,'phaw_30')
```

In [373]:

```
groupings = {'arthropod':[(2,2,1,2,2,1,1,1,1,1,2,2,2,2,0,0,0,0,0,0,0),4,'arthro.tids'],\
             'pan-arthropod':[(2,2,1,2,2,1,1,1,1,1,2,2,2,2,2,0,0,0,0,0,0),4,'panart.tids'],\
             'pan-crustacea':[(2,2,1,2,2,1,1,1,1,1,0,0,0,0,0,0,0,0,0,0,0), 4,'pancrus.tids'],\
             'crustacea':[(0,0,1,2,2,1,1,1,1,1,0,0,0,0,0,0,0,0,0,0,0), 4,'crust.tids'],\
             'multicrustacea':[(0,0,0,0,2,1,1,1,1,1,0,0,0,0,0,0,0,0,0,0,0),3,'multicrus.tids'],\
             'allotriocarida':[(2,2,1,2,0,0,0,0,0,0,0,0,0,0,0,0,0,0,0,0,0),-1,'allotrio.tids'],\
             'malacostraca':[(0,0,0,0,2,1,1,0,0,0,0,0,0,0,0,0,0,0,0,0,0),2,'malacos.tids'],\
             'amphipod':[(0,0,0,0,2,2,0,0,0,0,0,0,0,0,0,0,0,0,0,0,0),-1,'amphipod.tids'],\
             'copepod':[(0,0,0,0,0,0,0,2,2,2,0,0,0,0,0,0,0,0,0,0,0),-1,'copepod.tids'],\
             'remipedia + hexapod':[(2,2,2,0,0,0,0,0,0,0,0,0,0,0,0,0,0,0,0,0,0),-1,'remi_hexa.tids'],\
             'branchiopod + copepod':[(0,0,0,2,0,0,0,2,2,2,0,0,0,0,0,0,0,0,0,0,0),2,'bran_copepod.tids'],\
             'branchiopod + malacostraca':[(0,0,0,2,2,1,1,0,0,0,0,0,0,0,0,0,0,0,0,0,0),2,'bran_mala.tids'],\
             'branchiopod + multicrustacea':[(0,0,0,2,2,1,1,1,1,1,0,0,0,0,0,0,0,0,0,0,0),2,'bran_mult.tids'],\
             'mandibulata':[(2,2,1,2,2,1,1,1,1,1,2,0,0,0,0,0,0,0,0,0,0), 4,'mandib.tids'],\
             'myriapod + chelicerata':[(0,0,0,0,0,0,0,0,0,0,2,2,2,2,0,0,0,0,0,0,0),-1,'myria_cheli.tids'],\
             'chelicerata':[(0,0,0,0,0,0,0,0,0,0,0,2,2,2,0,0,0,0,0,0,0),-1,'chelic.tids']}
for title,data in groupings.items():
    getInfo(cCount,sps,pCount,data[0],title,data[1])
```

```
pan-crustacea
Number of total groups: 23
Number of inclusive groups: 3269
Number of P.haw genes: 40

multicrustacea
Number of total groups: 40
Number of inclusive groups: 2347
Number of P.haw genes: 137

malacostraca
Number of total groups: 107
Number of inclusive groups: 824
Number of P.haw genes: 427

branchiopod + malacostraca
Number of total groups: 4
Number of inclusive groups: 881
Number of P.haw genes: 4

amphipod
Number of total groups: 180
Number of inclusive groups: 180
Number of P.haw genes: 409

pan-arthropod
Number of total groups: 15
Number of inclusive groups: 5523
Number of P.haw genes: 79

branchiopod + multicrustacea
Number of total groups: 26
Number of inclusive groups: 2551
Number of P.haw genes: 62

mandibulata
Number of total groups: 7
Number of inclusive groups: 3381
Number of P.haw genes: 12

crustacea
Number of total groups: 11
Number of inclusive groups: 2668
Number of P.haw genes: 48

copepod
Number of total groups: 332
Number of inclusive groups: 1148
Number of P.haw genes: 0

arthropod
Number of total groups: 16
Number of inclusive groups: 5101
Number of P.haw genes: 115

remipedia + hexapod
Number of total groups: 2
Number of inclusive groups: 410
Number of P.haw genes: 0

branchiopod + copepod
Number of total groups: 0
Number of inclusive groups: 1252
Number of P.haw genes: 0

allotriocarida
Number of total groups: 13
Number of inclusive groups: 444
Number of P.haw genes: 0

chelicerata
Number of total groups: 74
Number of inclusive groups: 510
Number of P.haw genes: 0

myriapod + chelicerata
Number of total groups: 16
Number of inclusive groups: 596
Number of P.haw genes: 0
```

### Orthology analysis for complete proteomes

In [73]:

```
specNames = ['A.gam','D.mel','D.pul','P.haw',\
                         'L.sal','S.mar','S.mim',\
                         'M.mar','I.sca','H.dju','B.mal','T.spi','C.ele','H.sap','M.mus','B.flo']
sps, cCount, pCount, pIndex = loadOrthoResult('/home/share/projects/phaw_genome/draft_3.0/comp/blast2/',specNames,'phaw_30')
```

In [376]:

```
groupings = {'arthropod':                 [(2,2,2,2,2,2,2,2,2,0,0,0,0,0,0,0),-1,'arthro.tids'],\
             'pan-arthropod':             [(2,2,2,2,2,2,2,2,2,2,0,0,0,0,0,0),-1,'panart.tids'],\
             'pan-crustacea':             [(2,2,2,2,2,0,0,0,0,0,0,0,0,0,0,0), -1,'pancrus.tids'],\
             'crustacea':                 [(0,0,2,2,2,0,0,0,0,0,0,0,0,0,0,0), -1,'crust.tids'],\
             'multicrustacea':            [(0,0,0,2,2,0,0,0,0,0,0,0,0,0,0,0),-1,'multicrus.tids'],\
             'allotriocarida':            [(2,2,2,0,0,0,0,0,0,0,0,0,0,0,0,0),-1,'allotrio.tids'],\
             'branchiopod + copepod':     [(0,0,2,0,2,0,0,0,0,0,0,0,0,0,0,0),-1,'bran_copepod.tids'],\
             'branchiopod + malacostraca':[(0,0,2,2,0,0,0,0,0,0,0,0,0,0,0,0),-1,'bran_mala.tids'],\
             'branchiopod + multicrustacea':[(0,0,2,2,2,0,0,0,0,0,0,0,0,0,0,0),-1,'bran_mult.tids'],\
             'mandibulata':               [(2,2,2,2,2,2,0,0,0,0,0,0,0,0,0,0), -1,'mandib.tids'],\
             'myriapod + chelicerata':    [(0,0,0,0,0,2,2,2,2,0,0,0,0,0,0,0),-1,'myria_cheli.tids'],\
             'chelicerata':               [(0,0,0,0,0,0,2,2,2,0,0,0,0,0,0,0),-1,'chelic.tids']}
for title,data in groupings.items():
    getInfo(cCount,sps,pCount,data[0],title,data[1])
    
    outFile = open('/home/share/projects/phaw_genome/draft_3.0/comp/blast2/' + data[2],'w')
    outFile.write('\n'.join(getPhawIDs(pIndex, data[0],data[1])))

    outFile.close()
```

```
pan-crustacea
Number of total groups: 36
Number of inclusive groups: 772
Number of P.haw genes: 67

branchiopod + malacostraca
Number of total groups: 49
Number of inclusive groups: 49
Number of P.haw genes: 115

mandibulata
Number of total groups: 7
Number of inclusive groups: 855
Number of P.haw genes: 13

crustacea
Number of total groups: 16
Number of inclusive groups: 135
Number of P.haw genes: 51

chelicerata
Number of total groups: 104
Number of inclusive groups: 694
Number of P.haw genes: 0

multicrustacea
Number of total groups: 44
Number of inclusive groups: 44
Number of P.haw genes: 100

myriapod + chelicerata
Number of total groups: 23
Number of inclusive groups: 818
Number of P.haw genes: 0

pan-arthropod
Number of total groups: 16
Number of inclusive groups: 2900
Number of P.haw genes: 41

branchiopod + multicrustacea
Number of total groups: 16
Number of inclusive groups: 135
Number of P.haw genes: 51

arthropod
Number of total groups: 22
Number of inclusive groups: 2532
Number of P.haw genes: 105

branchiopod + copepod
Number of total groups: 26
Number of inclusive groups: 26
Number of P.haw genes: 0

allotriocarida
Number of total groups: 21
Number of inclusive groups: 506
Number of P.haw genes: 0
```

### Orthologous group expansion

For each orthologous group, a mean number of paralogs was calculated. Z-score for P.hawaiensis was calculated by:

```
(# of p.hawaiensis paralogs - mean paralogs) / std deviation
```

In [74]:

```
zFile = open('/home/share/projects/phaw_genome/draft_3.0/comp/inclusiveAnalysis/ortho.data')

groups = []
spStrings = {}
for line in zFile:
    meta = line.strip().split()
    gid = meta[0]
    
    if meta[-1] != 'nan':
        zscore = float(meta[-1])
        mean = float(meta[-3]) 
        std = float(meta[-2])
        taxa = int(meta[-4])
        spStrings[gid] = meta[-5]
        
        groups.append([gid,mean,std,zscore,taxa])

zFile.close()

speFile = open('/home/share/projects/phaw_genome/draft_3.0/comp/inclusiveAnalysis/SpeciesIDs.txt','r')
speIndex = {}
for line in speFile:
    meta = line.strip().split(':')
    sp = meta[1].strip().split('.')[0].split('_')[0]
    speIndex[meta[0].strip()] = sp
```

In [75]:

```
groups = pd.DataFrame(groups)
groups.columns = ['gid','mean','stdv','zscore','taxa']
```

In [76]:

```
print 'Number of orthologous groups with Parhyale hawaiensis:', commas(len(groups))
```

```
Number of orthologous groups with Parhyale hawaiensis: 6,394
```

In [457]:

```
print 'z-score distribution of all groups'
groups['zscore'].hist(bins=100,figsize=[10,6])
```

```
z-score distribution of all groups
```

Out[457]:

```
<matplotlib.axes.AxesSubplot at 0x27226c10>
```

In [389]:

```
print 'Number of groups with mean paralogs >= 5 and zscore >=2'
groups[groups['mean'] >= 5][groups['zscore'] >= 2]
```

```
Number of groups with mean paralogs >= 5 and zscore >=2
```

Out[389]:

|  | gid | mean | stdv | zscore | taxa |
| --- | --- | --- | --- | --- | --- |
| 6 | OG0000007 | 59.714286 | 115.804286 | 3.387489 | 14 |
| 16 | OG0000017 | 40.923077 | 52.166098 | 2.570193 | 13 |
| 41 | OG0000045 | 24.000000 | 23.425824 | 2.305148 | 13 |
| 53 | OG0000059 | 15.750000 | 12.745097 | 2.295000 | 16 |
| 55 | OG0000061 | 20.750000 | 24.413538 | 2.959424 | 12 |
| 59 | OG0000065 | 15.125000 | 10.215644 | 2.728658 | 16 |
| 73 | OG0000082 | 16.153846 | 19.417859 | 2.464028 | 13 |
| 78 | OG0000087 | 12.437500 | 7.968444 | 2.705986 | 16 |
| 79 | OG0000088 | 12.375000 | 8.313205 | 2.480993 | 16 |
| 92 | OG0000105 | 12.923077 | 13.504766 | 2.079038 | 13 |
| 109 | OG0000123 | 9.437500 | 9.307045 | 2.854021 | 16 |
| 130 | OG0000146 | 17.000000 | 32.341923 | 2.628168 | 8 |
| 137 | OG0000153 | 8.187500 | 3.728585 | 2.363497 | 16 |
| 159 | OG0000179 | 7.437500 | 4.729809 | 3.290302 | 16 |
| 165 | OG0000185 | 10.727273 | 11.584401 | 2.181617 | 11 |
| 171 | OG0000193 | 7.000000 | 2.806243 | 2.138090 | 16 |
| 177 | OG0000201 | 6.750000 | 3.326034 | 2.480432 | 16 |
| 188 | OG0000212 | 8.750000 | 10.001042 | 3.024685 | 12 |
| 235 | OG0000265 | 8.090909 | 11.196221 | 3.028619 | 11 |
| 245 | OG0000277 | 7.166667 | 6.504272 | 3.049278 | 12 |
| 255 | OG0000289 | 5.250000 | 1.713914 | 2.187975 | 16 |
| 272 | OG0000307 | 6.750000 | 8.535465 | 3.192562 | 12 |
| 325 | OG0000371 | 10.285714 | 18.382556 | 2.432430 | 7 |
| 360 | OG0000412 | 5.500000 | 6.934215 | 2.235293 | 12 |
| 375 | OG0000433 | 7.875000 | 9.033791 | 2.227747 | 8 |
| 425 | OG0000485 | 5.363636 | 7.865607 | 2.877892 | 11 |
| 438 | OG0000501 | 8.142857 | 8.424939 | 2.356948 | 7 |
| 454 | OG0000517 | 5.181818 | 6.478190 | 2.441760 | 11 |
| 685 | OG0000762 | 5.000000 | 5.312459 | 2.635314 | 9 |

In [91]:

```
spIndex = {'agam':'A.gambiae',\
           'dmel':'D.melanogaster',\
           'dpul':'D.pulex',\
           'phaw':'P.hawaiensis',\
           'lsal':'L.salmonis',\
           'smar':'S.maritima',\
           'smim':'S.mimosarum',\
           'mmar':'M.martensii',\
           'isca':'I.scapularis',\
           'hdju':'H.dujardini',\
           'bmal':'B.malayi',\
           'tspi':'T.spirialis',\
           'cele':'C.elegans',\
           'hsap':'H.sapiens',\
           'mmus':'M.musculus',\
           'bflo':'B.floridae',\
           }

order = ['agam','dmel','dpul','phaw','lsal','smar','smim',\
         'mmar','isca','hdju','bmal','tspi','cele','hsap','mmus','bflo']

def plotPara(grid):
    counts = dict([(speIndex[x.split(':')[0]],int(x.split(':')[1])) for x in spStrings[grid].split(';')])
    data = []
    for k in order:
        if counts.has_key(k):
            data.append([spIndex[k],counts[k]])
        else:
            data.append([spIndex[k],0])
            
    s = pd.Series([x[1] for x in data],[x[0] for x in data])
    s.plot(kind='bar',figsize=[8,4])
```

### OG0000045 - expansion

78 P.hawaiensis genes in this ortho-group containing the following domains:

```
Ig_2    105
Ig_3    59
C2-set_2        37
I-set   33
ig      14
V-set   9
fn3     5
```

For each of the 78 P.hawaiensis genes, the following hits can be found:

```
Sidestep protein        55
Nephrin         33
Protein turtle-like protein A   32
Neural cell adhesion molecule   21
Protein turtle-like protein B   17
Kin of IRRE-like protein 3      14
Neuronal growth regulator 1     13
```

In [92]:

```
plotPara('OG0000045')
```

### OG0000059 - expansion

45 P.hawaiensis genes contains following domains:

```
Ig_2    75
I-set   67
Ig_3    47
ig      39
V-set   27
fn3     3
```

Hits:

```
Lachesin        42
Putative neural cell adhesion molecule l1       10
Limbic system-associated membrane protein       8
Protein CEPU-1  7
Klingon         6
```

In [99]:

```
plotPara('OG0000059')
```

### OG0000061 - expansion

93 Genes with following domains:

```
Ig_2    102
V-set   95
I-set   87
Ig_3    50
ig      37
Lectin_C        1
Sod_Cu  1
```

Hits:

```
Neurotrimin     44
Defective proboscis extension response  34
Lachesin        21
Dpr6    20
Limbic system-associated membrane protein       19
Hemicentin-2    17
Putative defective proboscis extension response         17
Immunoglobulin domain-containing protein-3      12
Transmembrane and immunoglobulin domain-containing protein      11
Putative limbic system-associated membrane protein      11
Leucine-rich repeats and immunoglobulin-like domains protein 1  11
```

In [100]:

```
plotPara('OG0000061')
```

### OG0000065 - expansion

43 P.hawaiensis genes with domains:

```
Peptidase_M1    27
ERAP1_C 24
Peptidase_MA_2  23
```

Hits:

```
Aminopeptidase N        38
Aminopeptidase N        28
Protease m1 zinc metalloprotease        19
Putative puromycin-sensitive aminopeptidase     14
Aminopeptidase N-like protein   11
Peptidase family M1     9
Alanyl aminopeptidase   5
```

In [101]:

```
plotPara('OG0000065')
```

### OG0000087 - expansion

34 P.hawaiensis genes with domains:

```
adh_short       26
KR      19
adh_short_C2    7
Epimerase       6
NAD_binding_10  4
WW      1
```

Hits:

```
Retinol dehydrogenase 12        21
Dehydrogenase   5
Short-chain dehydrogenase       4
Dehydrogenase/reductase SDR family member 12    3
```

In [104]:

```
plotPara('OG0000087')
```

### OG0000088 - expansion

33 P.hawaiensis genes with domains:

```
Peptidase_C1    30
Inhibitor_I29   14
Peptidase_C1_2  3
```

Hits:

```
Cathepsin L     30
Cathepsin L-like cysteine proteinase    15
Cathepsin L-like protease       14
Cathepsin L1    14
Cathepsin l     13
Cysteine proteinase     12
Putative cathepsin l-like cysteine proteinase b         11
Cathepsin L-like proteinase     9
Cathepsin L-1   9
```

In [102]:

```
plotPara('OG0000088')
```

### OG0000153 - expansion

17 genes with domains:

```
PAX     18
Homeobox        4
HTH_23  3
Homeobox_KN     2
```

Hits:

```
Paired box protein Pax-6        5
Paired box protein      3
Gooseberry      3
Putative twin of eyeless        3
Paired box protein Pax-7        3
Paired box protein Pax-6        3
Eyeless         2
Paired  2
Eyegone 2
```

In [103]:

```
plotPara('OG0000153')
```

### Top bitscore hit for P. hawaiensis

Using the species blast results from OrthoFinder:

```
1. Concatenate all Blast4_*.txt files (P.hawaiensis blast to other species)
2. For each P.haw protein, find the top hit among all species by highest bitscore
3. If there are multiple top hit with equal bitscores, take the species with the most number of top hits
```

In [33]:

```
import sys
from collections import defaultdict
from collections import Counter

inFile = open('/home/share/projects/phaw_genome/draft_3.0/comp/blast/phaw.otherSpecies.blastp','r')
species = '''0: agam.proteome.fa
1: dmel.proteome.fa
2: stul.transdecoder.fa
3: dpul.proteome.fa
4: phaw_30.genes.final.prot.fa
5: even.transdecoder.fa
6: lvan.transdecoder.fa
7: eser.transdecoder.fa
8: cfin.transdecoder.fa
9: lsal.proteome.fa
10: smar.proteome.fa
11: smim.proteome.fa
12: mmar.proteome.fa
13: isca.proteome.fa
14: hdju.proteome.fa
15: bmal.proteome.fa
16: tspi.proteome.fa
17: cele.proteome.fa
18: hsap.proteome.fa
19: mmus.proteome.fa
20: bflo.proteome.fa'''.split('\n')
species = [x.split(':') for x in species]
species = dict([(x[0],x[1].split('.')[0].strip()) for x in species])

pids = defaultdict(lambda : defaultdict(lambda : 100))
groups = {'Malacostraca':set(['6','5']),\
          'Copepoda':set(['7','8','9']),\
          'Branchipoda':set(['3']),\
          'Remipedia':set(['2']),\
          'Hexapoda':set(['0','1']),\
          'Myriapoda':set(['10']),\
          'Chelicerata':set(['12','11','13']),\
          'Tardigrada':set(['14']),\
          'Nematoda':set(['15','16','17']),\
          'Chordata':set(['18','19','20'])}

for line in inFile:
    meta = line.strip().split()
    pid = meta[0]
    hid = meta[1]
    if pid.split('_')[0] != hid.split('_')[0]:
        ev = float(meta[-2])
        bscore = float(meta[-1])
    
        if ev < pids[pid][hid]:
            pids[pid][hid] = ev
```

In [34]:

```
hitCount = defaultdict(lambda : defaultdict(int))
for pid, data in pids.items():
    hits = data.items()
    hits.sort(key = lambda x : x[1])
    topHit = hits[0][0].split('_')[0]
    topHit_group = [k for k,v in groups.items() if topHit in v][0]
    hitCount['top'][topHit_group] += 1
    for ethresh in [1e-5,1e-10,1e-25,1e-50,1e-100]:
        sps = list(set([k.split('_')[0] for k,v in data.items() if v <= ethresh]))
        threshGroups = set()
        for sp in sps:
            threshGroups.add([k for k,v in groups.items() if sp in v][0])
        for g in threshGroups:
            hitCount[ethresh][g] += 1
```

In [56]:

```
hitTable = ListTable()
hitData = []
for group in ['Malacostraca',\
          'Copepoda',\
          'Branchipoda',\
          'Remipedia',\
          'Hexapoda',\
          'Myriapoda',\
          'Chelicerata',\
          'Tardigrada',\
          'Nematoda',\
          'Chordata']:
    row = []
    row.append(group)
    for ethresh in ['top',1e-5,1e-10,1e-25,1e-50,1e-100]:
        row.append(hitCount[ethresh][group])
    hitData.append(row)
    hitTable.append(row)
hitTable.insert(0,['Clade','Top hit','1e-5','1e-10','1e-25','1e-50','1e-100'])
hitTable
```

Out[56]:

|  |  |  |  |  |  |  |
| --- | --- | --- | --- | --- | --- | --- |
| Clade | Top hit | 1e-5 | 1e-10 | 1e-25 | 1e-50 | 1e-100 |
| Malacostraca | 13060 | 19124 | 17618 | 13820 | 9932 | 5819 |
| Copepoda | 1511 | 17099 | 15114 | 10969 | 7084 | 3642 |
| Branchipoda | 580 | 15333 | 13339 | 9779 | 6498 | 3421 |
| Remipedia | 296 | 9177 | 6980 | 3467 | 1305 | 155 |
| Hexapoda | 765 | 14480 | 12855 | 9604 | 6367 | 3305 |
| Myriapoda | 656 | 15616 | 13739 | 9914 | 6353 | 3305 |
| Chelicerata | 1751 | 16696 | 14787 | 10794 | 6959 | 3554 |
| Tardigrada | 154 | 13346 | 11278 | 7503 | 4376 | 1776 |
| Nematoda | 425 | 13791 | 11824 | 7903 | 4794 | 2195 |
| Chordata | 1756 | 15612 | 13607 | 10006 | 6469 | 3267 |

In [57]:

```
hitData = pd.DataFrame([x[1:] for x in hitData],index = [x[0] for x in hitData])
hitData.columns = ['Top Hit','less than 1e-5','less than 1e-10','less than 1e-25','less than 1e-50','less than 1e-100']
```

In [69]:

```
hitData.plot(kind='bar',figsize=[20,6])
```

Out[69]:

```
<matplotlib.axes.AxesSubplot at 0x1fa23550>
```
