# Supplementary material for: The genome of the crustacean Parhyale hawaiensis, a model for animal development, regeneration, immunity and lignocellulose digestion
Source: eLife. 2016 Nov 16;5:e20062. doi: 10.7554/eLife.20062 (PMC5111886; doi:10.7554/eLife.20062)
Supplement: Source code 6. — Analysis of microRNAs and putative lncRNAs in Parhyale. DOI: http://dx.doi.org/10.7554/eLife.20062.051 [file elife-20062-code6.htm]

Notebook


In [1]:

```
import gzip, os, sys
#import custom functions for displaying tables, bash commands
sys.path.append(os.path.abspath("/home/damian/"))
from dk_ipython import *
from IPython.display import HTML
import pandas as pd
import seaborn as sns
import matplotlib.pyplot as plt
import scipy.stats as ss
import scipy as sp
import numpy as np

%matplotlib inline
HTML(addToggle())
```

Out[1]:

The raw code for this IPython notebook is by default hidden for easier reading.
To toggle on/off the raw code, click here.

### RNA analysis

Infernal 1.1.1 was used to scan the genome using RFAM 12.0 database:

```
cmscan --tblout out.tbl --cut_ga --cpu 5 RFAM.cms input.fa > out.cmscan
```

Hits above the gathering threshold of each covariance model (CM) were kept.

The following RNA classifications were found:

```
     83 Cis-reg;
      1 Cis-reg; frameshift_element;
      1 Cis-reg; IRES;
      7 Gene;
      1 Gene; antisense;
      1 Gene; lncRNA;
    155 Gene; miRNA;
     44 Gene; ribozyme;
    250 Gene; rRNA;
     52 Gene; snRNA; snoRNA; CD-box;
      3 Gene; snRNA; snoRNA; HACA-box;
      4 Gene; snRNA; snoRNA; scaRNA;
    199 Gene; snRNA; splicing;
      4 Gene; sRNA;
   1032 Gene; tRNA;
      1 Intron;
```

These 6 Cis-reg classifications were found in 83 Cis-reg loci:

```
    RF00032,Histone3,Cis-reg;
    RF00222,IRES_Bag1,Cis-reg; IRES;
    RF00383,IS1222_FSE,Cis-reg; frameshift_element;
    RF00485,K_chan_RES,Cis-reg;
    RF01068,mini-ykkC,Cis-reg;
    RF02253,IRE_II,Cis-reg;
```

### Long non-coding RNAs

A single conserved lncRNA was identified with RFAM:

```
phaw_30.0077363 490     573     RF02046,Sphinx_1,Gene; lncRNA;
```

The assembled transcriptome was used as a starting point to identify more potential long non-coding RNAs. A series of filters were used to discard potentially coding transcripts:

```
- Discard any TransDecoder predicted coding transcripts. This is a general filter which should take care of transcripts with genic hexamer frequency and possible PFAM domains.
- Discard any transcript with a PFAM domain or hit to Unprot. In case TransDecoder missed out on transcript with mutations, we dsicard any transcript with possible coding homology.
- Discard any transcript that, when mapped to the genome, overlaps with an Augustus predicted gene.
- Discard any transcript that has less than 90% mapping coverage to the genome
```

From the leftover transcripts, the longest Trinity designated isoform was kept for each Trinity gene.

From an initial 292,924 transcripts:

- 188,061 single exon transcripts were left over
- 32,223 multi-exon transcripts were leftover

Merging the genomic loci of all potential long-non-coding RNA by coordinate results in 163,670 genomic loci.

By blasting the 220,284 putative long non-coding RNAs to the genomes of selected arthropod species (D.melanogaster, D. pulex, L. salmonis, D. magna, S. maritima, Z. nevadensis), we find 4 putative long non-coding RNA with a hit in one or more of the genomes with an alignment length at least 50% of the RNA length:

```
ID               exons    Length     Species:alignment%
c103103_g1_i1    1        304        dmag:0.57
c105813_g1_i1    2        269        dpul:0.39,dmag:0.19,znev:0.59    
c180715_g1_i1    1        300        dmag:0.63
c296232_g1_i1    2        233        dmel:1.0
```

### miRNA

There are 155 loci classified as miRNA. Some loci were counted twice due to hits on both positive and minus strand. The following lists the 26 metazoan miRNA families classfied from 42 loci after filtering for strand redundancy:

```
  * designates conserved bilateran micro-RNA (18 found)

  1 bantam (protostomia)
  2 let-7*
  1 mir-1*
  4 mir-10
     mir-10 (eumetazoa)
     mir-10 (eumetazoa)
     mir-100*
     mir-993 (ecdysozoa)
  1 mir-124*
  2 mir-133*
  1 mir-137*
  4 mir-184*
  2 mir-190*
  3 mir-2 (protostomia)
  1 mir-210*
  2 mir-219*
  2 mir-282 (mandibulata)
  2 mir-263*
  1 mir-276 (arthropoda)
  1 mir-285*
  2 mir-33* (eumetazoa also)
  1 mir-34/449*
  2 mir-46*
      mir-281
      mir-281
  2 mir-307 (protostomia)
  1 mir-71*
  1 mir-8*
  2 mir-9*
  1 mir-iab-4 (arthropoda)
```

The following groups of mirnas are on the same contigs:

```
mir-10, let-7
mir-210, mir-263
mir-71, mir-2, mir-2
```

### Potential novel mirnas

To find all possible potential micro-RNA precursor loci on the genome, we used miRPARA software to scan the genome.

```
perl miRPARA.pl sequences.fasta
```

miRPARA outputs predicted mirnas in the following format:

```
mloci50464:239-306      acUCGUGAAGCAGGAGAUCCGCUGUCcgauguagaagcgaucguggaugcagcggauuaccuccacgg    mloci50464:239-306:3_24 UCGUGAAGCAGGAGAUCCGCUGUC        5P      0.924832        

Sequence name and coordinates, precursor sequence, mature sequence coordinates, 5p/3p, and a SVM probability score
```

A predicted precursor sequence from miRPARA can possibly have multiple good mature miRNA coordinates that differ by just a few bases according to the folding energy.

For each sequence, the possible mature miRNA coordinates were consolidated (union of all coordinates) to produce a single region where the mature miRNA might be located.

**Small RNA sequencing reads**

A previous publication investigated the expression of small RNAs during P. hawaiensis embryogensis from stage 1-4 and stage 4-14:

```
Blythe MJ, Malla S, Everall R, Shih Y-h, Lemay V, Moreton J, et al. (2012) High Through-Put Sequencing of the Parhyale hawaiensis mRNAs and microRNAs to Aid Comparative Developmental Studies. PLoS ONE 7(3): e33784. doi:10.1371/journal.pone.0033784
```

ABI SOLiD 4 was used to sequence the small RNA libraries. The raw reads are deposited at:

```
http://www.ebi.ac.uk/ena/data/view/ERP001160
```

The small RNA reads were mapped to the genome to produce a bed file. For each potential micro-RNA precursors found with miRPARA, we generate a tag count based intersection of the precursor loci with small RNA read mapping location.

We then classified these potential precursors based on known mature miRNA from mirbase and also small RNA reads mapping from a previous publication:

**mirbase classification**

For each family in mirbase, there are a list of 5P and 3P mature miRNA sequences available. The 5P and 3P for each family was aligned and a HMM was created. These HMMs were then used to scan the potential precursors sites for a match:

```
mafft --auto mir-5p.fa > mir-5p.mafft.fa
hmmbuild --rna mir-5p.mafft.hmm mir-5p.mafft.fa
cat *.hmm > mirbase.hmm
hmmprcess mirbase.hmm
hmmscan --domtblout --cpu 32 predicted.dom mirbase.hmm predicted.fa > predicted.hmmscan
```

Only mirbase families where there are multiple 5p or 3p sequeuces (paralogs in a single species or orthologs in multiple species) were used to construct the HMM. In total there are 733 HMMs constructed from 5p or 3p mature sequences representing 443 families.

In [2]:

```
prefix = '/home/share/projects/phaw_genome/draft_3.0/smallrna/'
```

In [3]:

```
precFile = open(prefix + 'mirpara.tile.coords.hmm.renamed.smallRNA.genomeFeature.cleaned.hox.consolidated.bed')
prec = []

def ov(a,b):
    a.sort()
    b.sort()
    return min(a[1],b[1]) - max(a[0],b[0])

for line in precFile:
    ref, start,end, pid, seq, five, three, hmm, count, feature, featureType, multi, distance, hox = line.strip().split('\t')
    five = map(int, five.split(','))
    three = map(int, three.split(','))
    hmmFam = 'NA'
    hmmScore = -100
    
    if hmm != 'NA':
        hmm = hmm.split(',')
        hmm[0] = hmm[0].split('_')[0]
        hmm[1] = float(hmm[1])
        hmm[2] = int(hmm[2])
        hmm[3] = int(hmm[3])
        
        if max(ov([hmm[2],hmm[3]],(five)),ov([hmm[2],hmm[3]],(three))) >= 8:
            hmmFam = hmm[0]
            hmmScore = hmm[1]
    coords = ref + ':' + start + ',' + end
    count = int(count)
    prec.append([pid,hmmFam,hmmScore,count,coords, feature, featureType, multi, int(distance), hox])
    
precFile.close()
```

In [4]:

```
prec = pd.DataFrame(prec)
prec.columns = ['pid','fam','bscore','count','coords','feature','feature type','exons','distance','hox']
```

In [6]:

```
print 'Number of potential precursors:', commas(len(prec))
print 'Number of mirbase precursors identified by miRPARA: 69'
print 'Avg bitscore of HMMs found on mirbase precursors indentified by miRPAR: 13.316'
print
print 'Number of mirbase families scanned: 733'
print 'Log2 of # of mirbase families: 9.5'
print
print 'Number of potential precursors with HMM hit (any score):', commas(len(prec[prec['fam'] != 'NA']))
print 'Number of potential precursors with HMM hit (>=5 bitscore):', \
commas(len(prec[prec['fam'] != 'NA'][prec['bscore'] >= 5]))
print 'Number of potential precursors with HMM hit (>=10 bitscore):', \
commas(len(prec[prec['fam'] != 'NA'][prec['bscore'] >= 10]))
print 'Number of potential precursors with HMM hit (>=13.316 bitscore):', \
commas(len(prec[prec['fam'] != 'NA'][prec['bscore'] >= 13]))
print 'Number of potential precursors with HMM hit (>=15 bitscore):', \
commas(len(prec[prec['fam'] != 'NA'][prec['bscore'] >= 15]))
print
print 'Number of potential precursors with at least 1 small RNA read mapping:', commas(len(prec[prec['count'] > 0]))
print 'Number of potential precursors with at least 5 small RNA read mapping:', commas(len(prec[prec['count'] >= 5]))
print 'Number of potential precursors with at least 10 small RNA read mapping:', commas(len(prec[prec['count'] >= 10]))
print 'Number of potential precursors with at least 100 small RNA read mapping:', commas(len(prec[prec['count'] >= 100]))
print
print 'Number of potential precursors within a lncrna or gene region:', commas(len(prec[prec['distance'] == 0]))
print 'Number of potential precursors within 100bp a lncrna or gene region:', commas(len(prec[prec['distance'] <= 100]))
print 'Number of potential precursors within 1000bp a lncrna or gene region:', commas(len(prec[prec['distance'] <= 1000]))
print
print 'Number of potential precursors within a lncrna region:', \
commas(len(prec[prec['feature'] == 'lncrna'][prec['distance'] == 0]))
print 'Number of potential precursors within a lncrna exonic region:', \
commas(len(prec[prec['feature'] == 'lncrna'][prec['distance'] == 0][prec['feature type'] == 'exon']))
print 'Number of potential precursors within a lncrna intronic region:', \
commas(len(prec[prec['feature'] == 'lncrna'][prec['distance'] == 0][prec['feature type'] == 'intron']))
print
print 'Number of potential precursors within a gene region:', \
commas(len(prec[prec['feature'] == 'gene'][prec['distance'] == 0]))
print 'Number of potential precursors within a gene exonic region:', \
commas(len(prec[prec['feature'] == 'gene'][prec['distance'] == 0][prec['feature type'] == 'exon']))
print 'Number of potential precursors within a gene intronic region:', \
commas(len(prec[prec['feature'] == 'gene'][prec['distance'] == 0][prec['feature type'] == 'intron']))
print
print 'Number of potential precursors within 1kb of a feature, more than 0 tag counts, and bitscore >= 10',\
commas(len(prec[prec['distance'] <= 1000][prec['count'] > 0][prec['bscore'] >= 10]))
print 'Number of potential precursors with at least 1 tag count and within 100bp of a feature:',\
commas(len(prec[prec['distance'] <= 100][prec['count'] > 0]))
```

```
Number of potential precursors: 1,345,483
Number of mirbase precursors identified by miRPARA: 69
Avg bitscore of HMMs found on mirbase precursors indentified by miRPAR: 13.316

Number of mirbase families scanned: 733
Log2 of # of mirbase families: 9.5

Number of potential precursors with HMM hit (any score): 36,159
Number of potential precursors with HMM hit (>=5 bitscore): 27,353
Number of potential precursors with HMM hit (>=10 bitscore): 5,960
Number of potential precursors with HMM hit (>=13.316 bitscore): 994
Number of potential precursors with HMM hit (>=15 bitscore): 214

Number of potential precursors with at least 1 small RNA read mapping: 419,575
Number of potential precursors with at least 5 small RNA read mapping: 32,699
Number of potential precursors with at least 10 small RNA read mapping: 14,956
Number of potential precursors with at least 100 small RNA read mapping: 1,403

Number of potential precursors within a lncrna or gene region: 330,074
Number of potential precursors within 100bp a lncrna or gene region: 825,387
Number of potential precursors within 1000bp a lncrna or gene region: 880,588

Number of potential precursors within a lncrna region: 102,315
Number of potential precursors within a lncrna exonic region: 32,223
Number of potential precursors within a lncrna intronic region: 70,092

Number of potential precursors within a gene region: 227,759
Number of potential precursors within a gene exonic region: 40,019
Number of potential precursors within a gene intronic region: 187,740

Number of potential precursors within 1kb of a feature, more than 0 tag counts, and bitscore >= 10 1,196
Number of potential precursors with at least 1 tag count and within 100bp of a feature: 259,989
```

In [7]:

```
print 'Bitscore distribution of all precursors with HMM hits'
prec[prec['fam'] != 'NA'][prec['bscore'] >= 0]['bscore'].hist(bins=50,figsize=[10,5])
```

```
Bitscore distribution of all precursors with HMM hits
```

Out[7]:

```
<matplotlib.axes.AxesSubplot at 0x59c2650>
```

In [8]:

```
print 'Bitscore distribution of all precursors with HMM hits and within a lncrna or gene feature'
prec[prec['fam'] != 'NA'][prec['distance'] == 0]['bscore'].hist(bins=50,figsize=[10,5])
```

```
Bitscore distribution of all precursors with HMM hits and within a lncrna or gene feature
```

Out[8]:

```
<matplotlib.axes.AxesSubplot at 0x7b912d0>
```
